# Identifying suicide-related language in smartphone keyboard entries among high-risk adolescents

**DOI:** 10.1038/s41746-026-02921-x

**Published:** 2026-06-25

**Authors:** Paul Alexander Bloom, Isaac N. Treves, David Pagliaccio, Isabella Nadel, Emma Wool, Hayley Quinones, Julia Greenblatt, Natalia Parjane, Katherine Durham, Samantha Salem, Esha Trivedi, Hanga Galfalvy, Nicholas B. Allen, Deanna M. Barch, Ashley Blanchard, David Brent, Lauren S. Chernick, Peter S. Dayan, Caroline Hoyniak, Karla Joyce, Jaclyn S. Kirshenbaum, Lilian Y. Li, Joan Luby, Simryn Molina, Giovanna Porta, Zoe Price, Eva Purvin, Alexander Saul Rosenberg, Koustuv Saha, Stewart A. Shankman, Adela Schwartz, Soorya Ram Shimgekar, Jamie Zelazny, Randy P. Auerbach

**Affiliations:** 1 Department of Psychiatry, Columbia University, New York, NY USA; 2 Washington University School of Medicine, Washington University, St. Louis, MO USA; 3 Department of Psychology, University of Oregon, Eugene, OR USA; 4 Ksana Health Inc., Eugene, OR USA; 5 Department of Emergency Medicine, Columbia University Medical Center, New York, NY USA; 6 Department of Psychiatry, University of Pittsburgh Medical Center, Pittsburgh, PA USA; 7 Department of Psychiatry and Behavioral Sciences, Northwestern University, Chicago, IL USA; 8 Western Psychiatric Hospital, University of Pittsburgh Medical Center, Pittsburgh, PA USA; 9 University of Illinois Urbana-Champaign, Urbana, IL USA; 10 School of Nursing, University of Pittsburgh, Pittsburgh, PA USA

**Keywords:** Health care, Medical research, Psychology, Psychology

## Abstract

Adolescent suicide rates have risen over the past two decades, underscoring the need for improved risk detection strategies. Although natural language processing (NLP) tools are increasingly used to flag suicide-related content, little is known about how such approaches perform on adolescents’ smartphone communications. Addressing this gap, this study leverages passively collected smartphone data to identify suicide-related language in adolescents’ keyboard usage via NLP. We developed a lexicon of suicide-related adolescent language and validated it with labeled data (*N* = 171,468 text entries; e.g., messages, web searches), demonstrating higher performance in identifying suicide-related text than few-shot prediction with large language models (LLMs) and lexicons not designed for youth. Across two independent cohorts at elevated suicide risk (Ns = 208 & 257; >6 million text entries), lifetime suicidal thoughts and behaviors (STB) and current suicidal ideation were associated with increased frequency of smartphone suicide-related language. Human coding indicated varied language, including authentic first-person current suicidal ideation (14.5%) and jokes or hyperbole (20.2%). Compared with the lexicon alone, human coding of suicide-related entries with first-person language showed stronger associations with STB history. These findings highlight that effective NLP-based tools for suicide prevention will require more nuanced and context-specific approaches to better distinguish suicidal intent.

## Introduction

Despite decades of research, suicide remains a leading cause of death among adolescents in the United States^1,2^. A range of risk factors, including psychiatric diagnoses, sociodemographic factors, prior suicidal behavior, and life stress, are linked to suicide prevalence at a population level^3^. However, these factors often lack the temporal precision needed to anticipate suicidal behavior in the hours, days, or weeks before suicide occurs^4^. Thus, a key challenge in suicide prevention is the identification of short-term risk.

Risk for suicidal thoughts and behaviors (STB) is dynamic, often fluctuating over minutes, hours, or days^5^. This motivates the use of real-time assessment tools, including passive data collection methods via personal smartphones, enabling continuous, unobtrusive monitoring of digital behaviors that may reflect psychological risk^6,7^. One especially promising data source is the text typed by adolescents on their smartphones. Typing via smartphone, whether for messaging, social media posts, or web searches, is a near-constant part of adolescents’ lives and often reflects internal experiences^8^. Natural language processing (NLP) studies have shown that language used online can precede mental health changes, including emerging suicidal ideation^9–14^. Certain linguistic markers, including increased use of first-person pronouns^15^, negative sentiment^16,17^, and anger^18^ have been linked to depressive episodes or suicide risk. The continuous timestamps in passively collected text data also open a new avenue for testing whether language markers of psychopathology and suicidal ideation vary by time of day^19,20^, with possible implications for treatment and improving the short-term prediction of STB.

Despite the promise of NLP for youth suicide prevention, existing approaches share several key limitations. Prior work has largely fallen into three categories: proprietary monitoring tools operated by technology companies^21^, public social media (e.g., Twitter, Reddit, Facebook), and formal clinical text such as electronic health records or crisis texting services. Each has meaningful shortcomings that limit the development of accurate, generalizable tools. First, technology companies increasingly deploy monitoring for suicide-related text content, for example, on social media platforms or via partnerships with school systems to flag high-risk messages on school-based platforms or devices^22,23^. However, these are proprietary systems^24^ and thus have not been subject to external peer review, such that little information is publicly available about their mechanisms. Second, extant research has largely focused on language in public language^25–30^ (e.g., public Twitter, Reddit, or Facebook posts^31–37^). However, most social media datasets lack rigorous assessments of clinical STB outcomes^12,18^, limiting the direct clinical relevance of work focused on these platforms. In addition, as disclosures of STB may be markedly different in private contexts^38^, more information on suicide-related language in private domains (e.g., direct messaging, private social media, web searches) is crucial for developing informed and generalizable tools.

Further, though methods to identify suicide-related text have been validated in adult populations or clinical data, such as electronic health records or crisis texting services^13,39–43^, little is known about how well these tools apply to adolescent smartphone communication. Mental health and suicide-related communications are likely much less frequent in everyday smartphone usage compared to dedicated health-related service platforms, or even compared to social media spaces (e.g., subreddits) focused on depression or suicide^26^. Thus, more work is needed to address unique challenges in detecting low base rate events (suicide-related text) in large datasets, including testing which NLP architectures perform best (e.g., rule-based lexicons, transformer-based models, large language models [LLMs]) in these settings.

An additional challenge is that adolescent smartphone language is often informal and idiosyncratic^44^, including slang, abbreviations, suicide-related jokes, emojis, and platform-specific jargon, which may not be captured by approaches developed in adult samples or clinical contexts^16,45^. Determining to what extent adolescent language reflects current first-person and clinically concerning suicidal ideation, as opposed to references to others (e.g., celebrity suicides, concern for peers) or non-serious expressions (e.g., jokes, hyperbole) will be crucial to the clinical utility of new tools. Research on Instagram direct messages indicates that adolescents reference suicide topics in varied ways^46^ (e.g., personal STB experiences, gossip, jokes), however, more work is needed to determine whether findings generalize to broader smartphone communications, and which types of suicide references are truly associated with suicidal thoughts and behaviors.

In the current study, we address these gaps by leveraging data from the Effortless Assessment Research System (EARS) smartphone platform^47^, which allows for passive collection of typed text entries (e.g., messages, web searches) across applications on adolescents’ personal smartphones. We analyze EARS data from two adolescent cohorts at elevated suicide risk (MAPS, PREDICT; 13–18 years old) and a younger adolescent cohort (PEDSI; 8–16 years old) with somewhat lower clinical acuity (see “Methods” for details). First, we developed a youth suicide lexicon tailored for adolescent smartphone data based on previously validated suicide lexicons. We evaluated the lexicon performance in identifying suicide-related language compared to two existing lexicons and a large language model (LLM; GPT-4o mini). The lexicon was validated against a subset of data labeled by trained human coders (MAPS & PREDICT; *N* = 171,468 entries and PEDSI; *N* = 54,221 entries). Second, across the adolescent cohorts (MAPS *N* = 208 and PREDICT *N* = 257; >6 million total text entries), we tested whether individuals with a STB history used suicide-related language more frequently than youth with no STB history. We also examined the frequency of suicidal language by time of day and by smartphone app type. Third, we hand-coded all 8247 suicide-flagged entries from a high-risk cohort (MAPS) to parse the type of content, including authentic expressions of suicidal thoughts versus jokes or hyperbole, timing (past versus present), and referent (first-person versus others) of suicide-related language. Last, we evaluated the performance of an LLM in discriminating current, first-person, and authentic expressions of STB, and tested whether a combined lexicon + LLM approach more strongly differentiated youth with STB history than the lexicon alone.

## Results

### A youth suicide lexicon detects adolescent suicidal language with high sensitivity

**Fig. 1:**
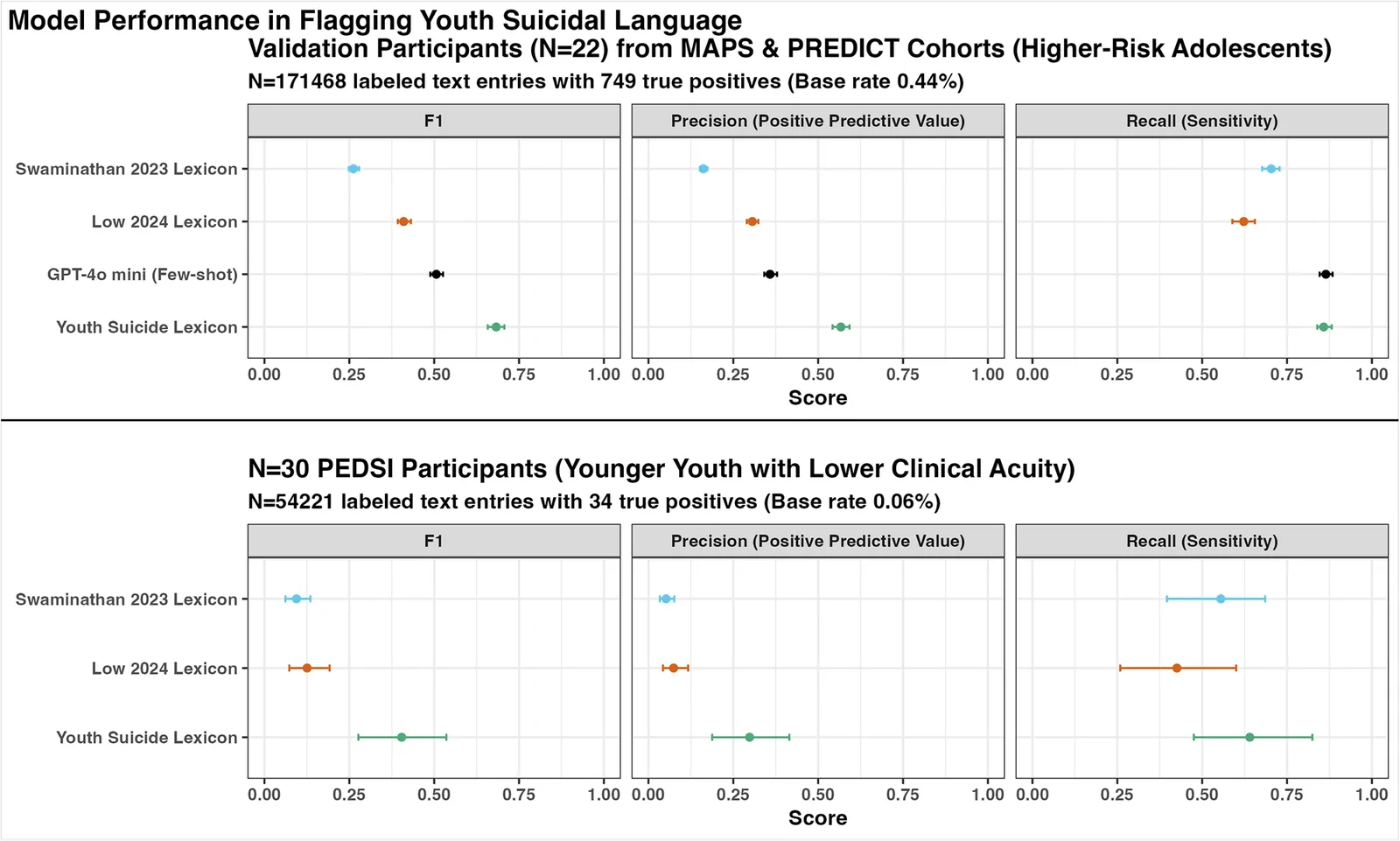
Model performance in detecting youth suicidal language. We compared our youth suicide lexicon and an LLM (GPT-4o mini) to existing approaches for detecting youth suicidal language within human-labeled entries from 22 participants in the MAPS and PREDICT (higher-risk adolescents, top panel) cohorts and 30 participants in the PEDSI (lower-acuity younger youth, bottom panel) cohort. The PEDSI cohort is referred to as lower-acuity due to lower rates of active SI and STB during the study period compared to the MAPS cohort. F1 (left; harmonic mean of precision and recall), precision (e.g., positive predictive value; the proportion of predicted positives that are true positives; center), and recall (e.g., sensitivity; the proportion of suicidal entries correctly labeled as suicidal; right) scores with bootstrapped 95% confidence intervals are shown on the x-axis for each model on the y-axis. GPT-4o mini was not evaluated in the PEDSI data. The base rate of suicide-related text in the labeled MAPS and PREDICT data was much higher (0.44%) than in the PEDSI data (0.06%). Model performance broken down separately in the MAPS and PREDICT validation participants is shown in Supplementary Fig. 2.

We first developed a youth suicide lexicon via making adaptations and additions to several existing lexicons (developed for crisis textline or tele-mental health systems)^13,39^ to better capture language used in adolescent smartphone communications (see “Methods”). We then compared the performance of our youth suicide lexicon to a LLM (GPT-4o mini, using few-shot prompting) and two of the existing lexicons used to adapt it^13,39^ (Fig. 1). In a subset of MAPS and PREDICT participants (*N* = 22; high-risk youth ages 13–18), *N* = 171,468 entries (not included in the data used to develop the youth suicide lexicon) were labeled by trained raters to test the lexicon, which included 749 entries human-labeled as suicide-related (base rate = 0.44%). Overall, the youth lexicon showed high recall (i.e., sensitivity, the proportion of suicidal entries correctly labeled as suicidal; 0.86, 95% CI = [0.84, 0.88]), but lower precision (i.e., positive predictive value; the proportion of predicted positives that are true positives; 0.57, 95% CI = [0.53, 0.59]) with an overall F1 = 0.68 (harmonic mean of precision and recall; 95% CI = [0.66, 0.71], Fig. 1). The lexicon overall outperformed GPT-4o mini (F1 = 0.51, 95% CI = [0.49, 0.53]), which had comparable recall (0.86, 95% CI = [0.85, 0.88]) but produced substantially more false positives, yielding lower precision (0.36, 95% CI = [0.34, 0.38]). The youth suicide lexicon and GPT-4o mini both outperformed existing lexicons not tailored for youth smartphone text. All models showed high (>0.98) specificity (e.g., correctly labeling non-suicidal entries as non-suicidal), though precision (i.e., positive predictive value; the proportion of predicted positives that are true positives) was consistently lower, likely driven by the low base rate (Supplementary Table 6).

### All lexicons showed lower performance in younger youth with lower suicide acuity

For the younger, lower-acuity participants of the PEDSI study (*N* = 30, ages 8–16), all *N* = 54,221 entries were labelled; 34 entries were identified as suicide-related (base rate=0.06%, Fig. 1*bottom panel*). Although the youth suicide lexicon (F1 = 0.40, 95% CI = [0.28, 0.54]) performed better than existing lexicon models in PEDSI data, all lexicons performed more poorly, likely because the low base rate of positive entries with suicidal language resulted in lower precision (e.g., a higher proportion of what was flagged were false positives).

### Youth with STB history and current SI use suicide-related language more frequently

**Fig. 2:**
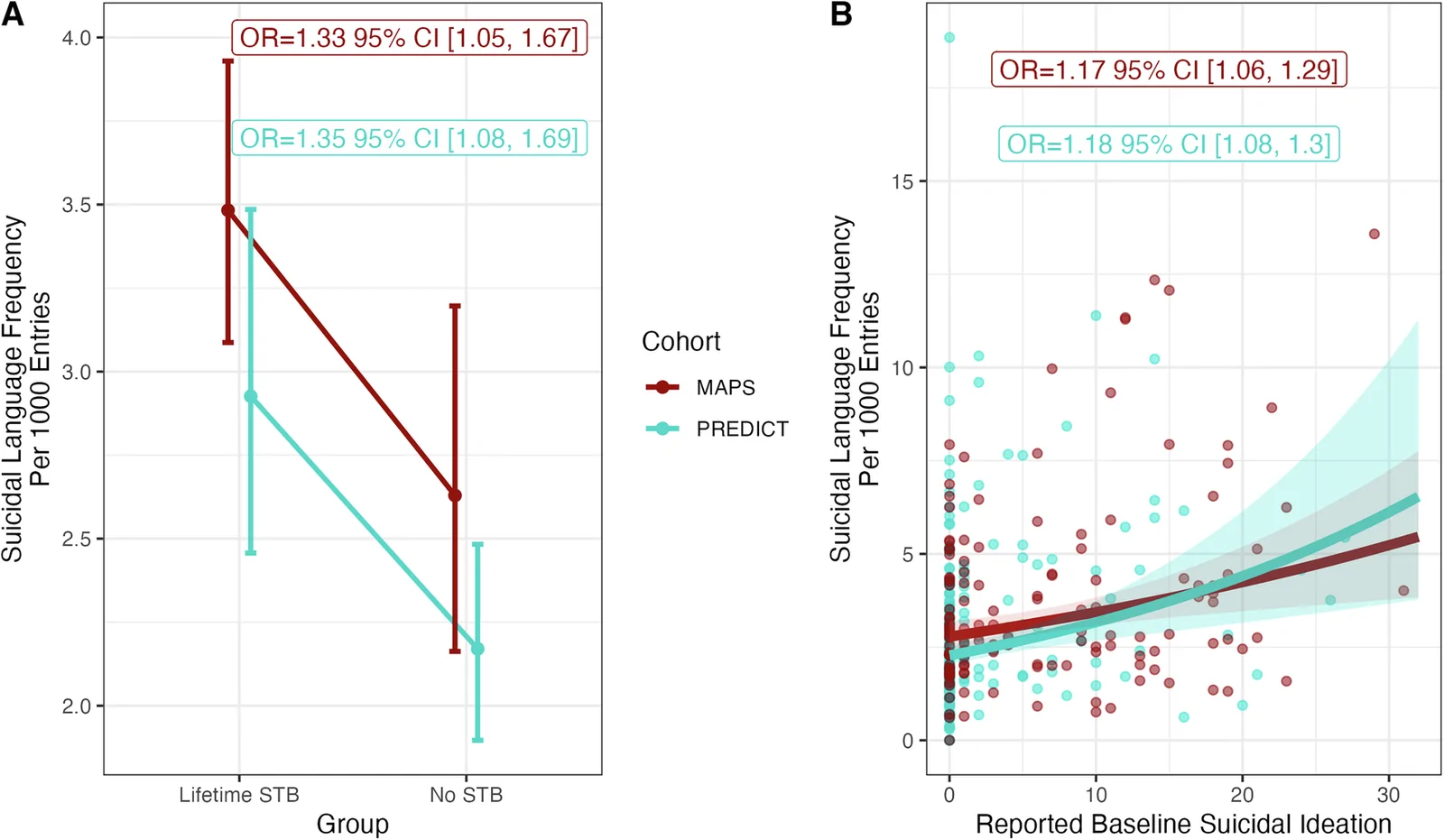
Suicidal language frequency as a function of suicidal thoughts and behavior (STB) history. **A** Frequency of suicidal language as a function of lifetime STB history (x-axis). Estimated rates of suicidal language flagged via our youth suicide lexicon per 1000 entries (e.g., multilevel logistic regression marginal predictions multiplied by 1000) are plotted on the y-axis as a function of lifetime STB history in the MAPS (red) and PREDICT (light blue) cohorts. Points and confidence intervals show estimates and 95% confidence intervals. **B** Frequency of suicidal language as a function of individual differences in self-reported baseline suicidal ideation. Rates of suicidal language flagged via our youth suicide lexicon per 1000 entries are plotted on the y-axis as a function of suicide ideation summary scores on the self-reported Beck Scale for Suicidal Ideation (maximum possible score is 38). Odds ratios indicate the change in odds of flagged suicidal language associated with a 1 SD increase above cohort mean suicidal ideation. Points show individual MAPS (red) and PREDICT (light blue) participants, and lines and shaded regions show model estimates and 95% confidence intervals. Odds ratios and 95% confidence intervals are from respective multilevel logistic regression models.

As further validation, we tested whether youth with STB (lifetime and/or current) expressed suicide-related language more frequently in their smartphone keyboard inputs compared to youth with no STB history. Specifically, we examined language flagged by the youth suicide lexicon only, as this approach had the highest F1 in identifying suicide-related text (Fig. 1). Overall, when using the lexicon to make predictions in the full MAPS and PREDICT datasets (>6 million total entries), only a small fraction of total entries was flagged as suicide-related language (MAPS = 0.39% [*N* = 140], PREDICT = 0.31% [*N* = 184]). Multilevel logistic regressions indicated that participants with lifetime STB history showed a significantly higher odds of flagged suicidal language compared to participants with no STB history (MAPS odds ratio [OR] = 1.33, 95% CI = [1.05, 1.67], PREDICT OR = 1.35, 95% CI = [1.08, 1.69]; Fig. 2A). Participants reporting more severe suicidal ideation at baseline showed a significantly higher frequency of suicidal language (MAPS OR = 1.17, 95% CI = [1.06, 1.29], PREDICT OR = 1.18, 95% CI = [1.08, 1.30]; odds ratios correspond to a 1 SD increase in self-reported suicidal ideation; Fig. 2B). Sensitivity analyses aggregating text on a daily or participant level, using negative binomial regression models, testing alternate inclusion criteria, and examining robustness to potential confounds yielded similar results (see Supplementary Results). Analyses also examined differences in rates of language flagged by the lexicon as a function of demographic factors (Supplementary Fig. 6).

### Suicide related language is flagged disproportionately at night and on social apps

**Fig. 3:**
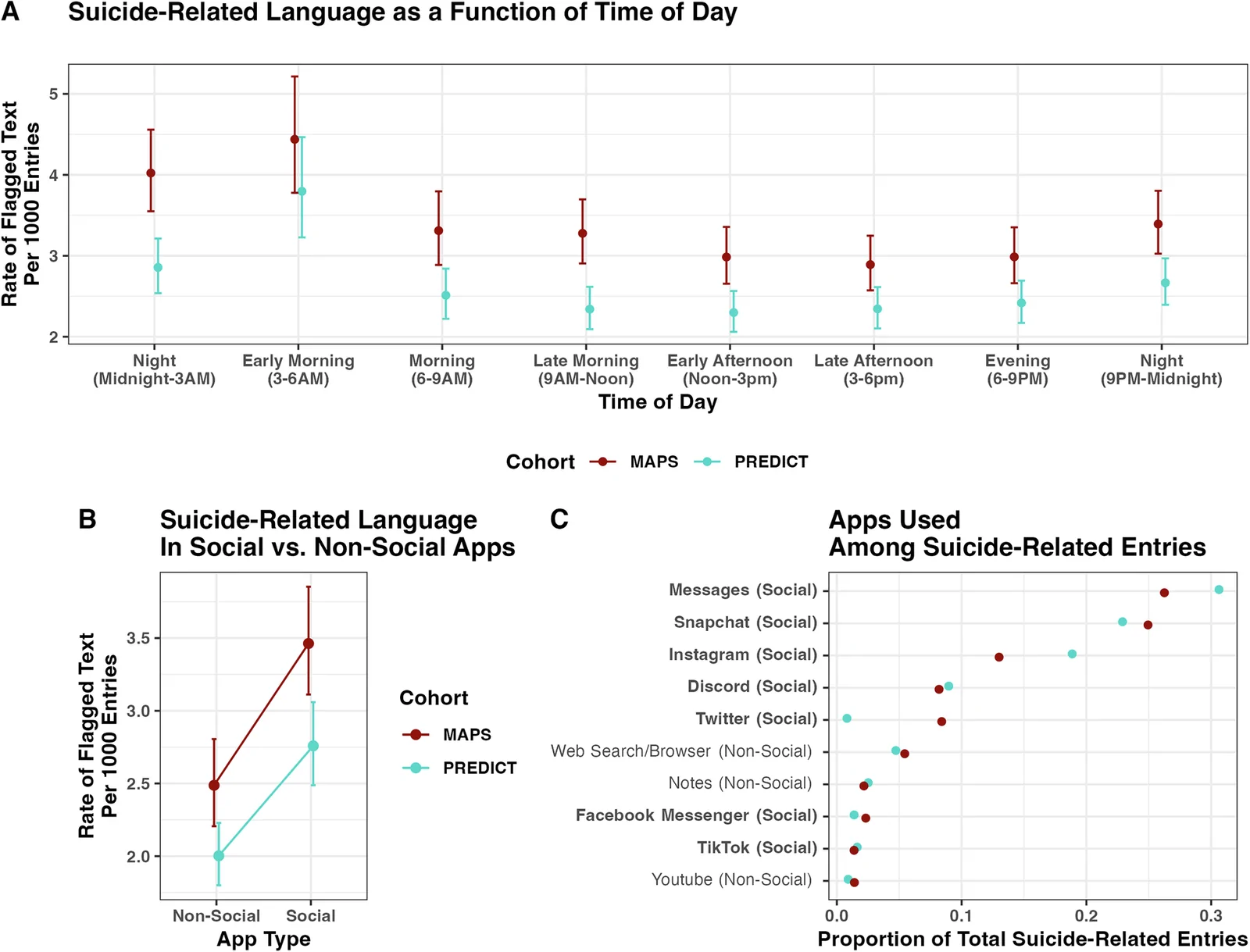
Suicide-related language as a function of time of day and app type. **A** Estimated rates of suicidal language flagged via our youth suicide lexicon per 1000 entries (e.g., multilevel logistic regression marginal predictions multiplied by 1000) are plotted on the y-axis as a function of time of day in the MAPS (red) and PREDICT (light blue) cohorts. Time of day of text entries was binned into discrete 3-h windows. **B** Estimated rates of suicidal language flagged via our youth suicide lexicon per 1000 entries are plotted on the y-axis as a function of app type (social versus non-social). For **A**, **B** points show multilevel logistic regression model predictions (multiplied by 1000) with 95% confidence intervals. **C** Overall distributions of suicide-related entries across apps in the MAPS (red) and PREDICT (light blue) cohorts. For each of the apps in which ≥1% of the total suicide-related entries were typed in either cohort (y-axis), the proportion of entries in that app out of all suicide-related entries is shown (x-axis). Apps are labeled as social (bold) versus non-social on the y-axis.

The probability that a given smartphone entry contained suicide-related text was highest between midnight-6 AM in both the MAPS and PREDICT cohorts, compared to other times of day (Fig. 3A and Supplementary Table 8). Suicide-related text was also more frequent in social than non-social apps in both cohorts (MAPS OR = 1.39, 95% CI = [1.30, 1.50], PREDICT OR = 1.38, 95% CI = [1.32, 1.44]). However, a non-negligible proportion of suicide-related entries (MAPS = 12.4%, PREDICT = 11.5%) occurred in apps not likely involving social communication (e.g., browser search, notes app, YouTube; Fig. 3C).

### Content of youth suicidal language is varied, and most often does not communicate current first-person suicidal thoughts and behaviors

**Fig. 4:**
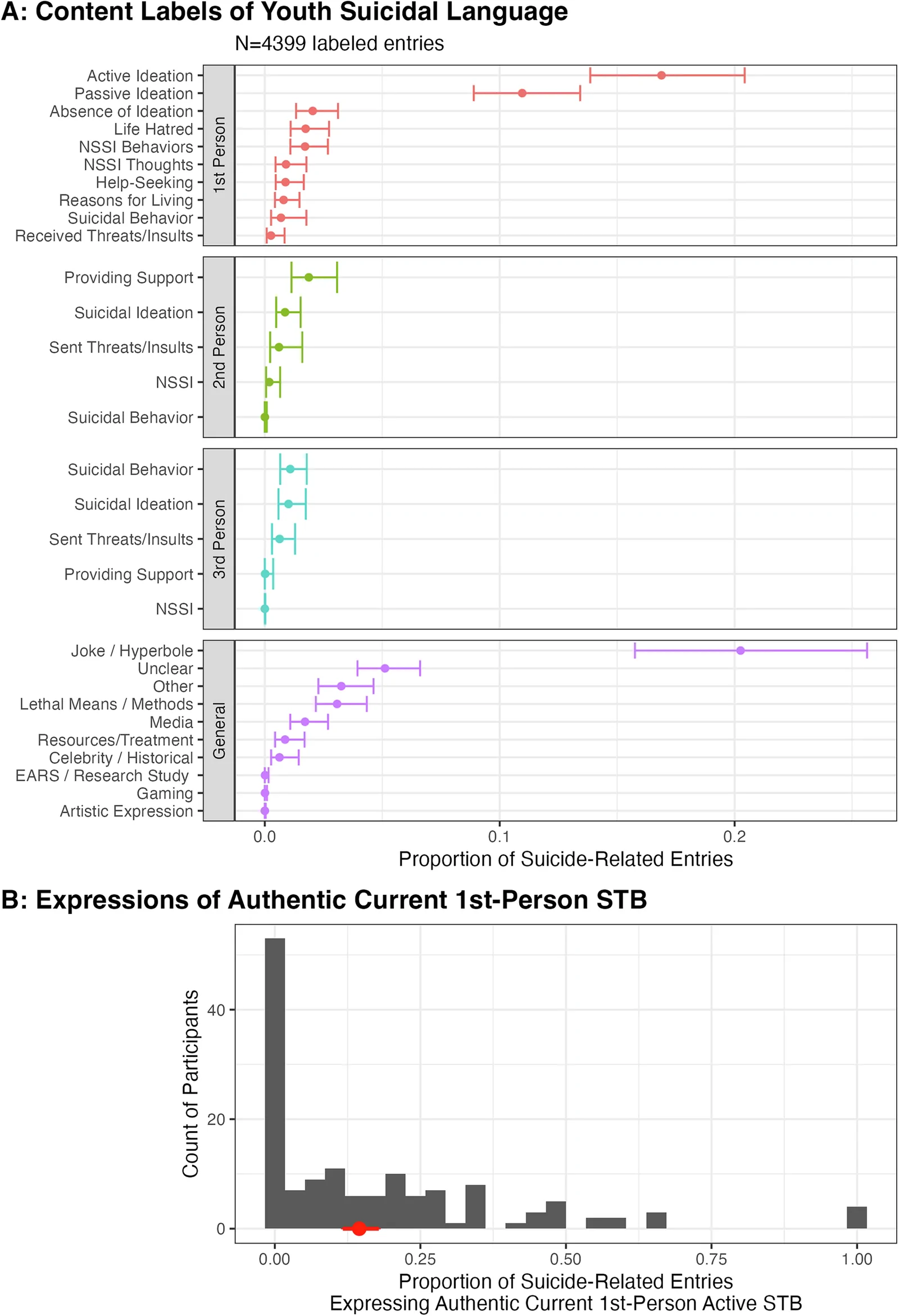
Content of suicide-related language. In **A**, the estimated proportion of suicide-related entries (x-axis) assigned to each content label (y-axis) by research staff in the MAPS cohort is shown. Out of 8247 flagged entries in the MAPS cohort, 4399 were coded as suicide-related (Precision = 0.53). Points and error bars show estimates and 95% confidence intervals from separate multilevel logistic regression models. From top to bottom, panels show 1st person (the participant’s own thoughts/behaviors), 2nd person (person the participant is communicating with), 3rd person (a third party different from who the participant is communicating with), and general labels. Labels were not mutually exclusive. In **B**, the estimated proportion of suicide-related entries expressing authentic current 1st person active suicidal thoughts is shown. The estimated group average proportion (and 95% CI) from a multilevel logistic regression model is shown in red on the x-axis, and the histogram shows the distribution of individual participants. Note: our coding of “authentic” is not a clinical coding referring to suicide risk, but rather indicates that expressions of STB were not judged to be jokes or hyperbole.

Trained staff coded the content of all 8247 entries flagged by the lexicon as suicide related in the full MAPS dataset into more specific categories (Table 1). Staff found 4399 entries (53%) to be suicide-related (consistent with the Precision = 0.57 within the smaller validation dataset; Fig. 1). Using multilevel logistic regressions (see “Methods”), we estimated the proportion of suicide-related text entries in each coding category (Fig. 4A). Overall, 74.6% of entries referenced current (within 24 h) or future STB, while the remaining entries referenced the past (10.0%) or had no temporal reference (15.4%). The most common individual content labels were jokes/hyperbole (20.2%, 95% CI = [15.8%, 25.6%]), first-person active suicidal ideation (16.9%, 95% CI = [13.9%, 20.4%]), and first-person passive suicidal ideation (11.0%, 95% CI = [8.89%, 13.4%]). Notably, only an estimated 14.5% (95% CI = [11.6%, 17.8%]) of all suicide-related entries were coded as communicating authentic (not jokes/hyperbole) first-person, active, and current suicidal ideation or behavior (including help-seeking; Fig. 4B), although there was significant heterogeneity between participants.

**Table 1:** Labels used by trained staff to code the content of suicide-related entries in the MAPS data

Category	Description	Exemplars (modified/paraphrased)
1st Person	Passive suicidal ideation/indifference to death	*“id be better off dead”,* *“wanna be dead”,* *“dying would be my only escape*”
	Active suicidal ideation/plans	“*I want to kill myself*”, “*gonna kms*”, “*gonna take all the pills*”, “*feeling suicidal*”, “*I wanted to kms yesterday*”
	NSSI thoughts	“*thinking of cutting*”, “*wanna slam my head on a wall*”
	NSSI behavior	“*I’m cutting myself*”, “*she saw my cuts on my arm*”
	Absence of suicidal thoughts (often a reassurance of safety)	“*Don’t worry I can keep myself safe now*”, “*was actually feeling better yesterday, not suicidal*”
	Accounts of being victimized	“*he told me to kms*”, “*stop telling me to shoot myself!*”
	Suicidal behavior	“*I tried to kill myself*”, “*I’m taking a whole bottle of pills now*”, “*I attempted but wound up in hospital instead*”
	Expressions of hatred of life/giving up on life	“*I hate life*”, “*I’m so done with life*”
	Describing disclosure or help-seeking for STB (therapy, meds, hotlines, social support)	“*thinking about kms please help me*”, “*I was feeling sewer so I called my therapist*”, “*self-help resources selfharm thoughts*”, “*told dad I was feeling suicidal*”
	Reasons for living	“*The only reason I’m staying alive is my IG friends*”, “*Not gonna kms, I need to be there for my mom*”
2nd Person	Checking in/offering support	“*please don’t kys*”, “*you have so many reasons to live*”
	Victimizing language/Threats/Insults	“*stfu go kys*”, “*go jump off a bridge*”
	Suicidal ideation (active or passive)	“*you always want to kill yourself on weeknights*”, “*I know you were feeling sewercidal last month*”
	Suicidal behavior	“*your suicide attempt last month*”
	NSSI thoughts/Behaviors	“*how did u keep her from seeing ur cut scars?*”
3rd Person	Concern/seeking support for a 3rd party	“*my friend might slit his wrists…pls help*”
	Victimizing language/Threats/Insults	*'fuck those yt football jocks, honestly just go kysss'*
	Suicidal ideation (active or passive)	*“hes been sewerslidal since the breakup”*
	Suicidal behavior	*“did you know she tried to kill herself?“*
	NSSI thoughts/Behaviors	*“shes thinking about making cuts in her arm”*
General	Jokes/hyperbole about suicide (including in response to stress/frustration)	*“missed the bus kms”, “hate history so much, kill me”, “my finger is bruised it wants to kms”*
	Suicide-related gaming language	*“blew himself up”, “lets play suicide mission”*
	Searching for/discussing information on lethal means	“*how many aspirin will kill you?*”, “*a rope that is strong enough to hang myself*”
	Searching/discussing therapy, meds, hotlines	“*suicide hotline*”, “*does increasing your ssri dose make you suicidal*”, “*dbt for suicidal ideation*”, “*trevor project suicide line*”, “*988*”
	References to EARS / being in a study about suicide	“*they’re studying my phone if I want to kms*”, “*talked to the Columbia doctor lady about my suicidal thoughts*”
	References to celebrity/public/historical suicides	*“cobain suicide”,* “*hitler death”, “robin williams killed”*
	Media references (e.g., bands, movies), art with suicidal themes	*“you see 13 reasons why?”, “when romeo kills himself”*
	Participant’s own poetry, lyrics, or other artistic expression with suicidal themes	*“the cliff was never so clear in my mind’s eye as when I talked you down from jumping off of it”*
	Meaning/suicidal intent unclear	*“kms”*
	Other suicide-relevant text not captured by other categories	*“ur bitmoji looks like it wants to commit suicide”,* *“doctors have a high suicide rate”*
Time Period	future => 24 h ahead, current = within 24 h, recent = 24 h–1 week past, past = 1 week–3 months, lifetime = >3 months, none = no temporal reference	*“if i don’t graduate i will just kms” (future), “she tried suicide last night” (current), “I was thinkin about kms a few days ago” (recent), “In March I was feeling suicide-y” (past), “2 years since my last suicide attempt!” (lifetime), “13 reasons why suicide” (none)*

Labels were not mutually exclusive, such that a given entry could have multiple labels. All labels were binary (presence vs. absence) except for Time Period. Exemplars are modified or paraphrased to preserve participant anonymity.

### Human labels of lexicon-flagged entries differentiate youth with STB history

**Fig. 5:**
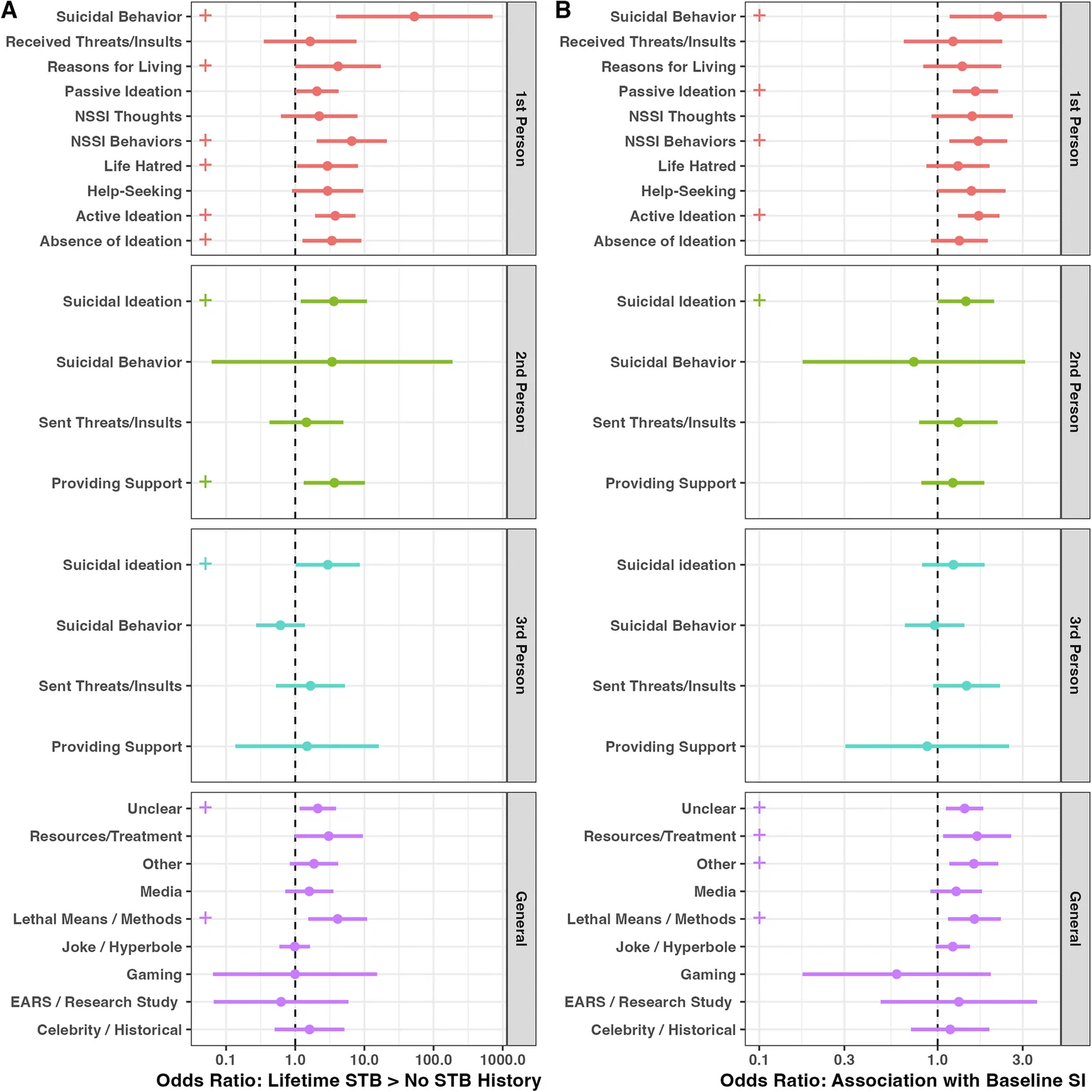
Associations between STB and specific coding labels for suicide-related text. **A** Odds ratios for differences between youth with lifetime STB history compared to youth with no lifetime STB history (Psychiatric Control) in the MAPS cohort are plotted on the x-axis. The large odds ratio for references to suicidal behavior among youth (**A**) was likely driven by the fact that only one participant (out of 41, 2.4%) without STB history had any such entries, compared to 31 participants (out of 99, 31.3%) with lifetime STB history (Supplementary Figs. 13–16). **B** Odds ratios for associations between baseline self-reported suicidal ideation (standardized, such that the odds ratio represents the change in odds associated with a 1 SD increase above the cohort mean suicidal ideation) are plotted on the x-axis. In both panels, odds ratio estimates and 95% confidence intervals are from multilevel logistic regression models where the outcome is whether or not a given text entry contained each language coding category (if a given entry text entry was not flagged by the youth suicide lexicon, the value was set to 0). Plus signs for a given row indicate significance at the *α* = 0.05 level.

Compared to the youth suicide lexicon results (Fig. 2), text entries identified as suicide-related by trained raters more strongly differentiated youth with and without an STB history (MAPS cohort: OR_Human_/OR_Lexicon_ = 1.50, 95% CI = [1.13, 1.83]; Supplementary Fig. 13). Youth with STB history showed a higher frequency of language human-coded as suicide-related in general (OR = 1.93, 95% CI = [1.28, 2.94]), as well as authentic first-person current active STB (OR = 3.27, 95% CI = [1.63, 6.55]), first-person more broadly (OR = 3.17, 95% CI = [1.79, 5.63]), second-person suicidal ideation (OR = 2.92, 95% CI = [1.41, 5.96]), and expressions of support for others experiencing STB (OR = 3.06, 95% CI = [1.15, 8.07]). Examination of individual language coding labels indicated that authentic (not jokes/hyperbole) first-person expressions most strongly distinguished youth with STB history (Fig. 5), in particular language referencing suicidal behavior (OR = 52.84, 95% CI = [3.91, 714.92], Supplementary Fig. 13–16). Only 1 participant without STB history used any language referencing suicidal behavior compared to about 31% of participants with STB history (Supplementary Fig. 15). However, the frequency of suicide-related jokes/hyperbole was not different as a function of STB history (OR = 0.98, 95% CI = [0.59, 1.64]; Fig. 5A and Supplementary Fig. 13A).

### LLM performance is limited in identifying first-person, current, and authentic suicidal language

GPT-4o mini was used with few-shot prompting to classify lexicon-flagged entries as referencing first-person, current, and authentic STB. This was compared to human coder labels from the MAPS cohort. Among the *N* = 8247 entries flagged by the lexicon (53% validated as suicide-related by human coders), GPT-4o mini performance was low in classifying first-person, current, and authentic STB references (F1 = 0.45, Recall = 0.65, Precision = 0.34), including both false positives and false negatives.

### A two-stage lexicon + LLM approach better differentiates youth with STB history than a lexicon alone

We used multilevel logistic regressions to test whether text flagged by first the youth suicide lexicon as suicide-related and then by few-shot prompting of GPT-4o mini as first-person, current, and authentic would more strongly differentiate youth with and without STB history. Compared to flagging text via our youth suicide lexicon alone (Fig. 2), the frequency of entries flagged by the two-stage lexicon+GPT-4o mini approach more strongly differentiated youth with STB history from psychiatric controls in both the MAPS (OR_Lexicon+LLM_/OR_Lexicon_ = 2.03, 95% CI = [1.34, 2.86]) and PREDICT (OR_Lexicon+LLM_/OR_Lexicon_ = 1.63, 95% CI = [1.12, 2.31]) cohorts (Supplementary Fig. 19). Youth with STB history showed a higher frequency of entries flagged by the lexicon+GPT-4o mini in both the MAPS (OR = 2.70, 95% CI = [1.61, 4.51]) and PREDICT cohort (OR = 2.38, 95% CI = [1.47, 3.83]). Similarly, the frequency of entries flagged by the lexicon+GPT-4o mini was more strongly associated with baseline self-reported suicidal ideation in the MAPS cohort **(**OR_Lexicon+LLM_/OR_Lexicon_ = 1.30, 95% CI = [1.12, 1.56]), although this difference was not significant in PREDICT **(**OR_Lexicon+LLM_/OR_Lexicon_ = 1.12, 95% CI = [0.96, 1.27]).

## Discussion

There is an urgent need for better strategies to identify suicide risk and guide prevention^48–50^. Day-to-day smartphone text inputs may offer a valuable window into how youth think and communicate about suicide, potentially informing timely and targeted interventions. However, existing research in this area remains limited, often focusing on specific platforms or contexts that fail to capture the informal and idiosyncratic nature of everyday adolescent smartphone inputs. The present study addresses these gaps by evaluating several approaches for identifying suicide-related language in passively collected smartphone text data from adolescents. Although suicide-related language was rare across all datasets (range = 0.06%-0.39% of entries), the newly developed youth suicide lexicon outperformed a few-shot LLM as well as lexicon models not tailored for adolescents in identifying suicide-related text (Fig. 1). Further, both lifetime STB history and current suicidal ideation were related to increased frequency of suicide-related language. Suicidal language occurred mostly in social communication apps and in entries between midnight and 6 am (Fig. 3)^19^. Critically, most suicide-related language did not communicate authentic expression of current active first-person STB that would be clinically concerning (Fig. 4). As it would be difficult to develop a lexicon to parse the nuance of authentic intent, we examined an LLM approach to understand this context. Yet, a few-shot LLM approach showed limited accuracy in identifying such authentic expressions of current active first-person STB. Nevertheless, both a two-stage lexicon+LLM approach and human coding of flagged entries better differentiated lifetime STB history compared to the lexicon alone (Fig. 5 and Supplementary Fig. 19). Taken together, this work highlights both the need for more nuanced domain-adapted approaches and broader challenges in identifying suicide risk via youth’s natural language on smartphones, which may provide a promising avenue to track risk and intervene in timely ways.

Encouragingly, both a youth suicide lexicon and GPT-4o mini showed high sensitivity (0.86; few false negatives) in detecting suicide-related language, and the youth suicide lexicon showed higher performance in two independent youth datasets compared to approaches not adapted to youth smartphone data (Fig. 1). Yet, false negatives (i.e., missed suicide-related text) are still a challenge, as some communications around suicide are context-specific or idiosyncratic (e.g., references to prior suicide attempts without explicitly suicide-related terms), such that accurate interpretation would require knowledge of the individual’s prior text entries or clinical history. Another major issue for risk detection applications is false positives, which were flagged particularly often among younger participants with lower STB acuity and participants with lower base rates of true suicide-related language (Fig. 1, *bottom panel* and Supplementary Figs. 2–4). GPT-4o mini with few-shot prompting also showed more frequent false positives relative to our youth suicide lexicon. These issues highlight that NLP approaches lacked the nuance to distinguish suicide-related language in this context, for example, from general death-related language, underscoring the need for further adaptation to this domain^51,52^.

Nevertheless, our youth suicide lexicon demonstrated construct validity, as adolescents with STB history more frequently used suicide-related language across two independent cohorts (Fig. 2). This was evident in two ways: both a history of STB and more severe self-reported baseline suicide ideation related to higher levels of suicide-related language. Flagged text was also most frequent between midnight and 6AM and in social apps (Fig. 3). These results have implications not only for risk detection but also for intervention strategies, particularly given the limited availability of support services during late-night hours.

A major challenge in translating extant research to clinical applications is a lack of data validating NLP performance in identifying suicide-related text^53,54^. The current study aimed to fill this gap by evaluating both lexicon and LLM approaches within ecologically valid text typed by youth in their day-to-day smartphone use. The weakness of the LLM approach differs from some recent work demonstrating that transformer-based models and LLMs have greater capabilities for detecting nuanced linguistic features compared to simpler, rule-based models^52,55^. However, there are several potential explanations. First, we chose to apply only out-of-the-box LLMs with few-shot prompting to ensure reproducibility (e.g., a version of GPT open to the public), rather than training a domain-specific model via fine-tuning. This stands in contrast to our youth suicide lexicon, which was developed specifically for adolescent smartphone communications, including iterative adaptation on a training set of youth smartphone entries. As recent work indicates that fine-tuning for specific instructions can improve classification performance beyond out-of-the-box approaches, including in mental health domains^56,57^, testing fine-tuned LLMs is therefore a key goal for future work. Second, the specific model (GPT-4o mini) or few-shot prompts used here may not have been optimal for detecting suicide-related language^58^. Third, prior work highlights that the classification of suicide-related text is a difficult task even for recently developed LLMs^59–61^. As new NLP tools emerge, continued testing combined with context-specific adaptation may yield more effective classification performance.

Importantly, identifying suicide risk in daily smartphone language may be fundamentally different from NLP work in other contexts. Crisis chat line transcripts, electronic health records, and mental health-focused social media spaces such as the R/SuicideWatch subreddit include more frequent and often explicit suicide related language^13,26,62^. Daily smartphone text entries, by contrast, are private and often informal, and suicide-related content occurs at an extremely low base rate amid vast amounts of everyday communication. These features make passive detection from smartphone data a distinct classification challenge. Context-specific adaptation to youth smartphone text, rather than off-the-shelf application of existing tools, is likely essential for progress in this space.

As a framework for informing future approaches, our findings also begin to identify subcategories of suicide-related language that may be most clinically salient. First-person references to current suicidal ideation and suicidal behaviors most consistently differentiated STB youth. The strongest effects were observed for language referencing suicidal behaviors, and active ideation language showed somewhat more consistent associations than passive ideation, though this distinction warrants replication (Fig. 5). However, language reflecting second- and third-person suicidal ideation was also more common among youth with STB history, suggesting that references to others’ suicidal experiences should not be dismissed in theoretical or clinical frameworks. For example, the linguistic expression of others’ suicidal experiences may suggest that youth with STB history are disproportionately embedded in social networks where suicidal behavior has occurred^63^. Exposures to others’ STB may increase the tolerance for potentially lethal behaviors, a pattern consistent with acquired capability within ideation-to-action theories of suicide holding that exposure to painful or provocative experiences, including engagement with self-harm methods, lowers the threshold for suicidal behavior^64–66^. Consistent with the role of acquired capability in STB risk, both language reflecting lethal means and non-suicidal self-injury (NSSI) behaviors were more commonly expressed by youth with STB history. Translating these group-level differences into a generalizable predictive model will ultimately require longitudinal analyses examining how these linguistic features evolve over time and precede clinical outcomes^67,68^, which are beyond the scope of the current manuscript. We therefore frame these findings as a foundation for subsequent predictive work.

However, our findings also caution against over-interpreting the presence of suicide-related language as a direct proxy for acute risk. Trained staff’s coding revealed that only an approximate 14.5% of suicide-related language (Fig. 4) indicated authentic expression of current active first-person suicidal ideation or behaviors. Further, a few-shot LLM could not classify such authentic, current, and first-person STB references with high accuracy. For example, the LLM struggled to distinguish hyperbolic versus authentic expressions of “kms” [“kill myself”], like “missed the bus kms” vs. “thinking about kms”. Thus, most flagged entries, even if containing suicide-related terms, might be clinical “false positives” as they do not explicitly communicate imminent suicidal intent. Furthermore, false positives may be even more common among cohorts at lower risk for suicide. Although prior work has also highlighted such varied content of youth suicidal language in other settings (e.g., Instagram direct messages)^46^, understanding the frequencies of different types of suicide-related language across all youth smartphone inputs may particularly help inform approaches for smartphone-based real-time monitoring of suicide risk. Posing a further challenge to using such approaches in clinical monitoring settings, clinician coders reported that even among serious expressions of STB, it was difficult to distinguish attention-seeking or suicidal gestures from true suicidal intent without broader context^69,70^. Further, youth differed in their use of suicidal language, with some more likely to express genuine intent than jokes or hyperbole, and model performance in identifying even the presence of any suicidal language varied widely between individuals (Supplementary Figs. 3–4).

Critically, it remains unknown which types of suicide-related language, if any, predict short-term risk. Some acute suicidal episodes may unfold without youth entering any suicide-related text into their smartphones^71^, while in other cases, even suicidal jokes or attention-seeking may signal vulnerability to STB. Understanding the short-term associations between specific forms of suicidal language and acute suicidal events is, therefore, a crucial step towards NLP-informed intervention strategies.

This work has several limitations that could be addressed in future research. Analyses are based solely on participants’ typed text, which lacks information on received text or the context (1:1 message versus group chat or public post). Donated social media data^72,73^, digital data sources capturing two-way communications^14,74^, or linked experience sampling data could provide complementary insight. Further, some participants needed to be excluded from primary analyses due to insufficient quantities of total text entries, although sensitivity analyses indicated that inclusion of these participants did not impact findings (Supplementary Figs. 9, 11, and12). Importantly, LLMs were not fine-tuned to adolescent smartphone communications in the current work. As fine-tuning to specific instructions has been shown to improve model performance in other mental health contexts^52,55^, such task-specific adaptation is a key next step for accurate detection of youths’ suicide-related smartphone communications.

Additionally, suicidal language may be most useful as a risk marker in tandem with linguistic metrics of psychiatric symptoms, distress, interpersonal stress exposures, broader multimodal digital phenotyping^75–77^, or direct clinical assessments, as crucially, these NLP methods cannot detect STB when youth do not enter any suicide-related text (or when they do not enter any text at all) via their smartphone keyboards. Further, although our preprocessing pipelines used translation, our youth suicide lexicon used only English terms, thus, generalizability to other languages may be limited, particularly for acronyms or slang^28,78,79^. Crucially, future work should examine which types of suicide-related language mark short-term risk for STB (e.g., within days or weeks)^70^.

Additionally, we note that the current findings do not indicate that a youth suicide lexicon should always be used rather than an LLM in detecting suicide risk. Lexicons and LLMs have their own strengths and weaknesses, each of which may be relatively more or less important depending on the domain of application. Compared to LLMs, lexicons are fast, interpretable, privacy-preserving, energy-efficient^80^, and cost-effective^13^. These characteristics are critical for feasibility in adolescent populations, where ethical concerns and implementation barriers around text monitoring are high^47,81^. On the other hand, LLMs are more flexible and capable of providing more nuanced outputs based on text inputs^25,52,82^, which may be critical in evaluating the intent of youths’ suicide-related communications.

One possibility for future work is a hybrid approach to risk detection. Although lexicons are blunt instruments with limited nuance, they may be well-suited for preliminary filtering of suicide-related text in multi-stage approaches. In this case, a high-recall lexicon could be used to flag a subset of potentially relevant entries for more sophisticated review, either by more transformer-based models (e.g., LLMs) or humans. In such settings, initial false positives may be an acceptable tradeoff if followed by a reliable second-stage evaluation. The current results indicate some support for such methods, in that a two-stage lexicon+LLM approach outperformed the youth suicide lexicon alone in differentiating youth with STB history (Supplementary Fig. 19). Yet, current results did not indicate reliable classification of first-person, current, and authentic expressions of STB via few-shot LLM prompting, indicating that more work is needed to develop such a layered strategy within the context of youth smartphone language.

Last, key implementation and ethical questions remain. Monitoring adolescents’ private texts raises concerns around consent, privacy, algorithmic biases, and risk of motivating youth to avoid disclosure^81,83–85^. We must rigorously evaluate whether any language-based monitoring meaningfully improves clinical outcomes, and for whom^86^. At present, our analyses indicated that the youth suicide lexicon flagged more frequent suicide-related language among female, sexual or gender minority, White, and younger adolescent (compared to older adolescent) participants (Supplementary Fig. 6). It is yet unclear without further validation analyses whether these differences reflect true differences in rates of suicide-related communication versus biased identification via the youth suicide lexicon. However, work in both the NLP and passive smartphone sensing literatures has demonstrated that model performance varies across sociodemographic subgroups, with predictions frequently exhibiting gender or race-related biases and performing more poorly in minoritized populations often underrepresented in research datasets^87–90^. This is a particular concern for opaque architectures such as LLMs, where limited interpretability makes bias difficult to detect and attribute. Before any deployment in youth monitoring contexts, these tools should be systematically evaluated for such biases and subjected to appropriate mitigation strategies.

A next step will be to identify which types of suicide-related language best predict near-term suicidal behavior, and whether monitoring tools (including LLMs) can support appropriate care^91^. One key issue is that youth are active participants in such systems, often acutely aware of monitoring, and dynamically adjust language to avoid detection (e.g., using coded terms like “kms” or “sewerslide”)^92^. Thus, both lexicons and LLM approaches would need to be updated regularly if deployed in clinical settings to match changes in youths’ language over time. Further, given monitoring, youth may limit suicidal disclosures or migrate to less-monitored platforms to maintain privacy, limiting the long-term utility of any detection system. Moreover, it is essential to recognize that mere presence of suicidal language is not inherently problematic. In many cases, digital spaces are crucial outlets to express distress, seek validation, or offer support to others^46,93^. Surveilling or intervening on suicide-related language may inadvertently stigmatize or suppress vital pathways for disclosure and support^94,95^. Thus, it is important not to conflate the reduction of suicide-related language with the prevention of suicide itself.

Taken together, the current work demonstrates that an adapted suicide lexicon can detect meaningful suicide-related language in adolescents’ everyday smartphone keyboard input data, and such language is associated with lifetime and current STB history. Although lexicons offer a scalable starting point for identifying suicide-related language, improving precision will require more sophisticated domain-adapted models that account for individual and contextual variation, especially in lower-risk or community-based samples where base rates may be particularly low. These findings lay important groundwork for developing real-time tools to support early detection and intervention in youth suicide risk.

## Methods

### Datasets

Three independent cohorts were analyzed as detailed below (see **Supplement** for full cohort information).

#### Mobile assessment for the prediction of suicide (MAPS) cohort

Adolescents ages 13–18 (*N* = 223) were recruited from the greater New York, NY and Pittsburgh, PA areas through psychiatric outpatient programs, pediatric emergency departments (ED), medical center research registries, and social media. Eligibility included: English fluency, current depressive, anxiety, and/or substance use disorder (as assessed via baseline diagnostic interviews), and ownership of a smartphone. Participants were excluded based on assessments of imminent suicide risk, active mania, lifetime psychosis, or an autism spectrum diagnosis. STB history was oversampled with 64.8% reporting current active suicidal ideation and/or 29.05% reporting a past year suicide attempt. Participants installed the EARS app at a baseline visit to collect data over a 6-month follow-up period^75,96–98^. Data were collected between 2018 and 2023 (see Supplement for Safety protocol).

#### Predicting the relapse and escalation of depression from interpersonal characteristics in teens (PREDICT) cohort

EARS data from a second cohort of adolescents at elevated risk for depression and suicide, compared to the broader population, were also analyzed to replicate findings using our suicide lexicon from the MAPS cohort. Adolescents aged 13–18 (*N* = 265) were recruited from the community and mental health clinics in New York and Chicago, IL, between 2020 and 2024. Inclusion criteria included Tanner stage ≥3, fluency in English, WASI-II IQ > 85, and ownership of a personal smartphone (iOS or Android). Exclusion criteria were current moderate/severe substance use disorder or a lifetime history of oppositional defiant disorder, conduct disorder, bipolar disorder, psychosis, or neurodevelopmental disorders. Participants were recruited across three groups, all of which were included in current analyses: current major depressive disorder (MDD, 15.6%), remitted MDD (47.6%), and healthy controls (36.8%). There were no suicide-related eligibility criteria. At baseline, 36.0% of participants reported a lifetime history of either active suicidal ideation or suicidal behavior. Participants installed the EARS app at a baseline visit to collect data over a 12-month follow-up period^15,99^.

#### Pediatric suicidality study (PEDSI) cohort

To test our youth suicide lexicon in younger youth, we analyzed data drawn from the Pediatric Suicidality Study (PEDSI), a longitudinal follow-up of a sample of children who participated in a randomized-control trial (RCT) for Preschool-Onset Major Depressive Disorder (PO-MDD) at ages 3–7 years (clinicaltrials.gov identifier: NCT02076425)^100^. In the original RCT (which ran from 2014 to 2018), children ages 3.0–6.99 years were recruited from community sources and mental health facilities in the St. Louis area, screened for depression symptoms, and those who met criteria for PO-MDD were randomized to receive Parent-Child Interaction Therapy–Emotion Development. Children who initially participated in the RCT or were assessed as a community sample were eligible to participate in the follow-up study, the PEDSI study, which began in 2019. Data from 30 youth ages 8–16 years (*M* = 12.6) were included in the current study (see “Supplement” for details). Participants installed the EARS app to collect data over a 38-day follow-up period. Of note, 47% (*n* = 14) had a lifetime history of STB (active suicidal ideation or suicide attempts) as reported by either the parent or the child on the Kiddie Schedule for Affective Disorders and Schizophrenia Present and Lifetime Version (K-SADS-PL). At the visit prior to EARS installation, 13% (*N* = 4) reported current active suicide ideation. The PEDSI sample is referred to as a “lower acuity” cohort in the current work in comparison to the high prevalence of lifetime and current active suicidal thoughts or behaviors in the MAPS cohort.

Study procedures for the current analyses of the MAPS and PREDICT cohorts were approved by the Columbia University Ethics Review Board (IRB00008612, Protocol #ACYY0850). Study procedures for the PEDSI cohort were approved by the Washington University St. Louis Ethical Review Board (FWA00002284, Protocol #201306070). In all cohorts, adolescent assent and caregiver permission were obtained for participants under the age of 18 years, and informed consent was provided by 18-year-old participants in MAPS and PREDICT.

### EARS keyboard input preprocessing

Passive smartphone keyboard inputs in all studies were collected by the EARS app, which was developed and maintained by Ksana Health, Inc^47^. Keyboard input data (1 or more text entries) was available for *N* = 208 (93.3%) MAPS participants, and *N* = 257 (98.8%) PREDICT participants. Each keyboard input (all characters typed in any app), was timestamped and labeled with the app used, then inputs were transformed into text entries (e.g., a message or search entry) using an algorithm identifying logical divisions (e.g., pauses, changes in app). Any blank entries, system-generated text, entries with only a single character (other than emojis), URLs, and duplicate entries within the same app with timestamps separated by less than 1 s were discarded. Text data underwent a multi-layered de-identification process to protect participant privacy. First, custom in-house algorithms by Ksana Health, Inc. removed sensitive personal information such as passwords, credit card numbers, and other structured sensitive data formats. Following this initial pass, we used Microsoft Presidio, an open-source, automated de-identification tool, to perform an additional layer of entity recognition and removal^101^. Presidio’s algorithms were configured to detect and redact personally identifiable information, including names, geographic locations, phone numbers, email addresses, and other common identifiers. Names of public and historical figures who died by suicide were retained in the text. In the MAPS and PREDICT cohorts, spell-correction and language detection were performed using a HIPAA-compliant instance of GPT3.5 for the MAPS entries used for model validation (GPT3.5 was used to conserve computational resources for this step relative to newer models). Messages longer than 3 words determined by GPT3.5 to be non-English were translated using a HIPAA-compliant instance of GPT-4. Primary analyses were also conducted on MAPS and PREDICT data without spell-correction and translation (see “Supplement”). Current analyses were conducted after all data were collected, therefore EARS data were not monitored in real-time for suicide-related language, and no clinical interventions (e.g., for study safety protocols) were made based on keyboard input data.

### Human labeling of suicide-related text

To evaluate lexicon and LLM approaches in identifying the presence of suicide-related language, trained staff labeled all EARS keyboard entries (algorithmically stripped of identifiers) for 5% of the MAPS participants (*N* = 11 participants, 121,468 entries) and from *N* = 11 PREDICT participants with the highest self-reported baseline suicidal ideation (50,000 entries). Thus, models were evaluated on a combined dataset of 171,468 entries typed by *N* = 22 participants.

Staff also labeled all keyboard entries from PEDSI participants with EARS keyboard input data (*N* = 30, 54,221 entries) for the binary presence versus absence of suicide-related language. Validation data did not include the 12,000 MAPS entries used for lexicon development. All types of suicide-related language, regardless of the exact linguistic content (e.g., including jokes, hyperbole), were flagged via this step (see coding manual in “Supplement”). Coders used the context of the app and surrounding messages to resolve ambiguities as to whether an entry was suicide-related or not, although no entries were coded as suicide-related purely based on context (e.g., an entry needed to have some plausible suicide-related content to be flagged). All MAPS and PREDICT entries were double-coded (PAB, IN, EW, JG, NP, SM, EP, ZP, AR), and reliability was excellent (Cohen’s kappa = 0.82). A third coder resolved all discrepancies to create a single binary consensus label for each entry. The base rate of suicide-related text in the labeled MAPS data was higher (0.54%) than in the PREDICT data (0.18%). PEDSI entries, which were fewer in total number than those coded for MAPS, were coded by a single trained coder (HQ).

### Youth suicide lexicon development

A youth suicide lexicon was adapted via combining terms from several prior studies reporting suicide lexicons or key suicide-related terms used in natural language processing^13,39,46,102,103^. Initial lexicon refinement was conducted via iterative adaptation using a training set of 12,000 entries from the MAPS dataset, as well as a set of posts from the r/SuicideWatch and r/Depression subreddits^104^. Several key modifications were made using custom Python code:

1. Slang or coded terms (to avoid detection by social media algorithms) used among young people to refer to suicide were added (e.g., “sewer slide”, “kms”, “unalive”, “yeet myself off a cliff”), and leet speak (e.g., “k1ll my3lf”)^105^.
2. Multi-word tokens were formed to reduce false positives. For example, instead of using “hang”, which returned many false positives (e.g., references to “hang out”), tokens were created for “hang mys*” and “hang yours*”. Such tokens often contained several grammatical variants.
3. Word boundaries were defined for certain tokens (e.g., “OD” for overdose) to avoid flagging other non-suicidal words containing such tokens (e.g., “good”), while other tokens (“sui”) were defined without word boundaries to be as flexible as possible.
4. Certain tokens were used only in pairs to flag text. For example, “pill” + any of (e.g., “die”, “OD”, “dead”, or “kill m”).
5. Additional lists of emojis potentially related to suicide (e.g., , , ) and names of public and historical figures that died by suicide (e.g., “Kurt Cobain”) were included as an optional variant of the lexicon.

Although the lexicon developed in this work is not designed to be universally applicable and will likely require adaptation for specific contexts, we have made both the lexicon and accompanying Python code for data extraction available at https://github.com/pab2163/youth_suicide_lexicon.

### A large language model to identify youth suicide language presence

Recent work has shown that transformer-based models, including large language models (LLMs) may be well-suited for identifying nuanced or abstract constructs in naturalistic text^52,55,82^. Accordingly, we tested a few-shot prompting strategy using GPT-4o mini (gpt-4o-mini-2024-07-18), a large language model trained on data up to October 2023 and designed for efficiency and batched tasks^106^. A HIPAA-compliant institutional version of the model was used such that input data would not be used for subsequent model training. Few-shot prompts represented a condensed version of the coding manual provided to human coders for labeling the presence versus absence of suicidal language in each algorithmically deidentified text entry. The prompt contained 8 labeled examples and asked the model to return a JSON object with a binary variable representing whether the model detected the presence of suicide-related language or not (see “Supplement” for full prompts). Zero-shot prompts (e.g., without labeled examples) were tested on several examples but were not applied to the MAPS and PREDICT validation data, as these prompts failed to strongly differentiate text indicating broader mental health concerns (e.g., depression, anxiety) from suicide-related language. Model temperature was set to 0 to ensure deterministic outputs. GPT-4o mini was not tested on the PEDSI data, as the members of the study team with access to the text data for this cohort did not have the same institutional access to the HIPAA-compliant GPT model. To maximize reproducibility, no fine-tuning was conducted in the current study (i.e., fine-tuned models based on the current data could not be shared without risk of exposing participants’ text data)^107^.

### Evaluation of models to detect youth suicidal language

To benchmark the performance of models, we compared the performance of the youth suicide lexicon, a large language model (GPT-4o mini), and two prior suicide lexicons validated in adult or unspecified populations (e.g., anonymous users on public social media platforms) via messages through tele-mental health and crisis text line systems^13,39^. Labeling of text via the Low et al.^13^ and Swaminathan et al.^39^ lexicons was done using explicitly suicide-related sub-constructs within these lexicons (see “Supplement” for labeling using all sub-constructs). We also used the construct-tracker Python package to identify text with high semantic similarity to tokens in the Low Lexicon using a sentence embedding model (see “Supplement”)^13^. Classification of suicidal language using transformer-based embeddings was also tested through the BERTopic topic modeling package (see “Supplement”). All model outputs were coded as a binary variable indicating if the model had identified any suicide-relevant text in each entry or not.

To validate models, we computed the precision (e.g., positive predictive value, true positives/true positives + false positives), recall (e.g., sensitivity; true positives/true positives + false negatives), and F1 (harmonic mean of precision and recall) statistics for each model in predicting binary human codings for whether each message was suicide-related. The 95% confidence intervals for model performance estimates within this validation set were computed via bootstrapping (iterative resampling of the full sample size with replacement) with 100 iterations.

### Analyses of flagged suicidal language in two youth datasets

#### Flagging suicidal language

As the youth suicide lexicon performed better than other approaches tested in identifying suicide-related language (Fig. 1), we used our youth suicide lexicon to label entries in the entire MAPS (2.14 million entries, 8247 (0.39%) flagged) and PREDICT (4.62 million entries, 14,650 (0.31%) flagged) datasets. We note that the large total number of entries (>6 million) is a key strength of the current work, as statistical power would be a major barrier to identifying patterns of rarely occurring suicide-related text in smaller datasets. As flagging based on emojis did not help model performance (Supplementary Fig. 1), emojis were not flagged in this step. After flagging the full MAPS dataset, human coders rated all flagged messages for the presence versus absence of suicidal content. Among the flagged entries, we calculated the precision/false positive rates. Because the PEDSI cohort was substantially smaller (*N* = 30, 54,211 total entries) with very few suicide-related entries, this cohort was not included in further analyses of flagged entries.

#### Statistical analysis

Analysis code is available at https://github.com/pab2163/identifying_suicide_related_language. Statistical modeling and data visualization were conducted in R version 4.5.2^108^. For between-person analyses in MAPS and PREDICT, participants with <1000 total text entries were excluded (MAPS *N* = 68, PREDICT *N* = 65), leaving for analysis MAPS *N* = 140, PREDICT *N* = 192. However, sensitivity analyses of all available data, including these participants, yielded virtually identical results (Supplementary Fig. 9). Logistic multilevel regression models were fit using the glmmTMB R package on the text entry level to model the probability of a given entry being flagged as suicide-related via our youth suicide lexicon^109^. All models were fit separately for each cohort, and with random intercepts for each participant. This modeling approach was conducted on the text entry level to avoid confounding based on the overall frequency of text entries. Results were also robust to negative binomial regression models that incorporated the log of daily text entry count as an offset term (see Supplementary Results). Further, there were no significant differences in the average daily number of total text entries as a function of STB history in either the MAPS (*β*_STB > Control_ = 19.9, 95% CI = [−13.7, 53.5]) or PREDICT cohorts (*β*_STB > Control_ = 14.2, 95% CI = [−8.4, 47.5]). As the frequency of flagged entries was very low, to ease interpretability, estimated marginal probabilities were multiplied by 1000 to show predicted rates of suicide-related text per 1000 entries.

#### Suicidal language as a function of STB history

Separate multilevel logistic regression models estimated the probability of suicide-related text as a function of STB history (e.g., group differences; Lifetime STB > No STB) in MAPS and PREDICT cohorts, covarying for participant age. In the MAPS cohort, lifetime STB history was determined via either any reported suicidal behaviors via the SITBI or active suicidal ideation via the SITBI or self-reported Beck SSI (*N*_STB History_ = 99, *N*_No STB_ = 41). In the PREDICT cohort, lifetime STB history was determined by any reported suicidal behaviors or active suicidal ideation (>2) via the C-SSRS or Beck SSI (*N*_STB History_ = 61, *N*_No STB_ = 131). Separate multilevel models estimated the probability of suicide-related text as a function of individual differences in baseline suicidal ideation reported via the Beck SSI (19-item score excluding 2 items assessing suicide attempts). For these models, odds ratios reflect the change in odds associated with a one standard deviation increase in SSI score from the cohort mean. Similar models examined differences in the probability of suicide-related text as a function of sex assigned at birth, sexual or gender minority status, race, ethnicity, and age (Supplementary Fig. 6).

#### Suicidal language as a function of time of day and app category

Multilevel logistic regression estimated the probability that a given text entry contains suicidal language as a function of time of day. Time was binned into discrete 3-h intervals (Midnight–3 AM, 3–6 AM, 6–9 AM, 9 AM–12 PM, 12–3 PM, 3–6 PM, 6–9 PM, 9 PM–Midnight). 27.8% of all MAPS entries and 26.4% of all PREDICT entries occurred between Midnight and 6 AM (Supplementary Fig. 7). Sensitivity analyses using mixed-effects negative binomial regression at a daily level yielded similar results (Supplementary Fig. 17). Separate models also estimated the probability of suicidal language in social versus non-social apps. A subset of entries (8.8% for MAPS, 22% for PREDICT) for which no app label was recorded via EARS due to technological challenges were excluded for this analysis. Apps were designated as social if one of their primary purposes involved social communication, including social media (e.g., Instagram, TikTok), messaging (e.g., iMessage, Facebook Messenger, Discord), or dating (e.g., Tinder, Grindr). For apps in which the vast majority of entries were entered, we used each app’s classification and description in the Apple App Store or Google Play Store to determine a social designation^15,16,110^ GPT3 was used to determine app classifications for the remaining apps, which together encompassed ~1% of all text entries. Non-social apps included platforms for video or music streaming (YouTube, Apple Music), notes and work (Excel, Notes), or browsing and searching (Chrome, Safari). 83.0% of MAPS entries and 82.5% of PREDICT entries were entered into social apps overall.

### Content of suicide-related entries

Trained research staff (P.A.B., I.N., E.W., J.G., N.P., K.D., S.S., and A.S.) coded the content of all entries in the MAPS dataset flagged by our youth suicide lexicon (*N* = 8247 text entries). Entries were algorithmically stripped of identifiers, and staff were masked to the clinical status (including STB history) of participants when coding text entries. Non-mutually-exclusive labels (31 total) for content were iteratively developed through consultation with clinically licensed members of the research team (Table 1). Labels focused on determining the referent of suicidal language (First person [the participant], second person [the person the participant is communicating with], or third person [a third party]), the timing referenced (e.g., current versus past), and whether suicidal language was serious in intent (versus jokes/hyperbole). We used separate intercept-only multilevel logistic regression models to estimate the average proportion of flagged entries with each label (e.g., via the group intercept), and critically, the proportion of entries labeled as communicating serious first-person active current suicidal thoughts or behaviors. This definition included entries that were a) labeled as first-person active ideation, help-seeking, or suicidal behavior and b) labeled with the current (within 24 h) or future time windows. Our coding of “authentic” (i.e., “serious”) was not a clinical coding referring to suicide risk, but rather indicated that the text was not judged to be jokes/hyperbole. Multilevel models (rather than raw proportions across all entries) were used to account for between-participant differences in total numbers of entries. Of note, 5.12% of entries were labeled as unclear, indicating a minority remained difficult to interpret even for human coders.

Equivalent multilevel logistic regression models (see *Suicidal Language as a Function of STB History)* to those used for flagged data estimated the frequency of these labels, as well as an omnibus label for all lexicon-flagged messages coded as suicide-related (e.g., true positives), as a function of STB history (Fig. 5 and Supplementary Figs. 13–16, 18). To test whether effects were larger for the omnibus human label compared to lexicon-flagged messages alone, we bootstrapped MAPS participants and compared odds ratios within each of 1000 bootstrap iterations. Such analyses were regarded as exploratory, thus no corrections for multiple comparisons were applied.

### LLM labeling of first-person, current, and authentic suicidal language

We evaluated GPT-4o mini (gpt-4o-mini-2024-07-18) in classifying first-person, current, and authentic suicidal language among the text entries flagged by the lexicon in the MAPS cohort (*N* = 8247 text entries). A few-shot approach was taken, with the prompt mirroring the human coding manual for determining when entries referred to first-person, current, active suicidal ideation or behaviors, and including 3 labeled examples (see “Supplement” for full prompt). To provide the model with potentially helpful context, the prompts also included the timestamp, contents, and app used for up to 100 text entries sent between 1 h before and 1 h after the focal flagged entry. Entries closest in time to the flagged entry were retained in cases of >100 entries sent during this window. As output, the model was prompted to provide a JSON with two fields: (1) binary label indicating whether the focal flagged entry indicated first-person, current, and authentic suicidal language or not, and (2) 1–3 sentences of reasoning. The model’s temperature parameter was set to 0 to ensure deterministic output. The F1, precision, and recall of this model were evaluated against the human labels of first-person, current, and authentic suicide-related language among these text entries in the MAPS cohort. The same model was applied to make inferences on lexicon-flagged entries in the PREDICT cohort, however no performance metrics were evaluated due to the absence of human content labels in the PREDICT datasets.

### Comparing a lexicon-only versus lexicon + LLM approach in differentiating STB youth

We tested whether combining the youth suicide lexicon with GPT-4o mini would more strongly differentiate youth with and without STB history compared to the lexicon alone (lexicon alone results shown by themselves in Fig. 2). In the lexicon + LLM approach, GPT-4o mini evaluated each lexicon-flagged entry and retained only those judged to be first-person, current, and authentic. We then fit the same multilevel logistic regression models described above (see “Suicidal language as a function of STB history”) to text flagged by each approach separately. To test whether the two approaches yielded different effect sizes in differentiating youth with STB history from psychiatric control youth, we bootstrapped participants (100 iterations) and compared odds ratios within each iteration (Supplementary Fig. 19). This procedure was applied to both the STB history models and the baseline suicidal ideation models for the MAPS and PREDICT cohorts, respectively.

### Inclusion and ethics statement

All authors meet authorship criteria and contributed meaningfully. Study procedures for the current analyses of the MAPS and PREDICT cohorts were approved by the Columbia University Ethics Review Board (IRB00008612, Protocol #ACYY0850). Study procedures for the PEDSI cohort were approved by the Washington University St. Louis Ethical Review Board (FWA00002284, Protocol #201306070).

## Supplementary information

Supplementary Information

## Data Availability

Data from the Mobile Assessment for the Prediction of Suicide (MAPS) cohort are shared via the NIMH Data Archive (NDA ID #3502) at https://nda.nih.gov/edit_collection.html?id=3052. Data from the Predicting the Relapse and Escalation of Depression from Interpersonal Characteristics in Teens (PREDICT) cohort are shared via the NIMH Data Archive (NDA ID #3401) at https://nda.nih.gov/edit_collection.html?id=3401. Given the sensitive nature of text data and the possibility that they may not be fully deidentified even after removing names, phone numbers, and other identifiers, raw text cannot be shared publicly to ensure compliance with participants’ consent.

## Code availability

Analysis code is available online at https://github.com/pab2163/identifying_suicide_related_language. Further, lower-level keyboard input preprocessing code is available at https://github.com/pab2163/auerbach_nlp.
