# Supplementary material for: Identifying suicide-related language in smartphone keyboard entries among high-risk adolescents
Source: NPJ Digit Med. 2026 Jun 25;9:797. doi: 10.1038/s41746-026-02921-x (PMC13669378; doi:10.1038/s41746-026-02921-x)
Supplement: Supplementary file 1 [file 41746_2026_2921_MOESM1_ESM.pdf]

Supplemental Information

Table of Contents

MAPS Cohort Information 2
PREDICT Cohort Information 4
PEDSI Cohort Information 5
Participants Excluded for Insufficient Total Keyboard Text Entries: 6
Alternative Approaches for Detection of Suicide-Related Language 7
Text Translation 7
Coding Manual for Presence vs. Absence of Suicide-Related Content 8
Few-Shot Prompts for Large Language Models 12
Additional Model Comparisons in Detecting Suicide-Related Language 16
Exemplars of Youth Suicide Lexicon Prediction Errors 22
Frequency of Lexicon-Flagged Suicidal Language as a Function of Demographic Factors 23
Time-of-Day Patterns in Text Flagged by the Youth Suicide Lexicon 25
Keyboard Text Entry Frequency by Time of Day & STB History 27
Flagged Text as a Function of STB History: Sensitivity Analyses 29
Flagged Text as a Function of Suicidal Ideation: Sensitivity Analyses 35
STB History and Human Coding of Flagged Entries 39
Comparison of Lexicon Flagging Along versus Human Labels of Lexicon-Flagged Text 42
Supplemental References 46

MAPS Cohort Information

Supplemental Table 1. MAPS Cohort Characteristics (N=208)

**Variable**	**N (%) or M (SD)**
Site (PITT)	108 (51.92%)
Assigned Sex at Birth (M)	42 (20.19%)
Race (Single-Choice)	
American Indian / Alaska Native	1 (0.48%)
Asian	22 (10.58%)
Black	29 (13.94%)
Multiracial	22 (10.58%)
Unknown or Not Reported	19 (9.13%)
White	115 (55.29%)
Age at Baseline (Years)	16.47 (1.66)
Hispanic Ethnicity	42 (20.19%)
Income	-
<25k	17 (8.17%)
>100k	80 (38.46%)
25-100k	67 (32.21%)
Unknown or Not Reported	44 (21.15%)
Parent With College Education	-
None	42 (20.19%)
1 or more	153 (73.56%)
Missing	13 (6.25%)
Heterosexual	-
No	54 (25.96%)
Yes	53 (25.48%)
Not Assessed	101 (48.56%)
Cisgender	199 (95.67%)
iPhone (versus Android)	171 (82.21%)
SSI Score (19 items)	6.11 (7.42)
MFQ Score	12.43 (6.48)
Lifetime NSSI Behavior	138 (66.99%)
Baseline Active Suicidal Ideation (SSI)	128 (62.44%)
Lifetime Suicide Attempt History	62 (30.1%)
Lifetime Active Suicidal Ideation (SITBI)	144 (69.9%)
SCARED Score	4.18 (2.35)

Note: a baseline self-report sexual orientation item was added to the study protocol after the study was ongoing and therefore only some participants were administered this item. 3 participants were missing baseline self-report measures (SSI, MFQ, SCARED). STB=suicidal

thoughts and behaviors, MFQ=Mood and Feelings Questionnaire (13-item), SSI = Beck Scale for Suicidal Ideation scores were calculated as the sum of the items 1-19, each of which was scored on a 0-2 scale (min=0, max=38), SCARED=Screen for Anxiety Related Emotional Disorders Child-Report (5-item).

MAPS Safety Protocol: If a participant reported a score of ≥4 (scale of 1-5) on the weekly suicidal ideation prompt and/or "Yes" to reporting a past week suicidal plan or attempt, this triggered a textbox on an adolescent's smartphone directing the adolescent to clinical resources, *"Based on your response, you indicated that you may feel unsafe. We encourage you to talk with your parent or guardian as soon as possible. If you cannot keep yourself safe, please call 911 or visit the closest emergency department. There also are hotlines that may be of help to you: (a) 1-800-SUICIDE, (b) 1-888-SUICIDE, (c) 1-800-273-TALK, (d) 1-800-252-TEEN (Hotline for Teens), (e) 1-800-850- 8078 (Hotline for LGBT Teens)."* Additionally, these responses also initiated a text message and email to all clinical staff. Upon receipt of the alert, the designated on-call clinical staff then contacted the adolescent participant. Once reached by telephone, clinical staff administered the Columbia Suicide Severity Rating Scale to assess imminent suicide risk. If necessary, clinical staff developed safety plans and bridged to emergency clinical services. In cases of all minors and when permitted by participants 18 years or older, clinical staff then discussed the assessment and associated action plan with parents. As EARS data were not monitored in real-time, no safety protocols were triggered via participant keyboard input data.

PREDICT Cohort Information

Supplemental Table 2. PREDICT Cohort Characteristics (N=257 with any baseline interview and keyboard input data)

Variable	N (%) or M (SD)
Lifetime STB History (C-SSRS)	90 (31.28%)
MDD History (Baseline)	-
Current MDD	37 (14.57%)
No Lifetime MDD	99 (38.98%)
Remitted MDD	118 (46.46%)
NYC Site (vs. Chicago)	145 (56.42%)
Assigned Sex at Birth (Male)	65 (26.75%)
Race (Single-Choice)	-
American Indian / Alaska Native	2 (0.82%)
Asian	42 (17.28%)
Black	32 (13.17%)
Multiracial	33 (13.58%)
Native Hawaiian / Pacific Islander	1 (0.41%)
Unknown or Not Reported	20 (8.23%)
White	113 (46.50%)
Age at Baseline (Years)	16.42 (1.55)
Hispanic Ethnicity	71 (29.22%)
Heterosexual	-
No	93 (36.19%)
Yes	144 (56.03%)
Missing	20 (7.78%)
Cisgender	226 (93.00%)
Lifetime Suicide Attempt (C-SSRS)	19 (9.00%)
Current Active Ideation (C-SSRS)	13 (6.16%)

Note: N=46 participants were missing C-SSRS data at baseline, N=3 were missing MDD history, N=14 were missing demographic information (assigned sex at birth, age, race, Hispanic ethnicity, and gender). Current Active Ideation was defined as within the last 2 months via the C-SSRS.

PEDSI Cohort Information

Supplemental Table 3. PEDSI Cohort Characteristics (N=30)

Variable	*N* (%)
Assigned Sex	19 M / 11 F
Race (Single-Choice)	
American Indian / Alaska Native	0 (0.0)
Asian	0 (0.0)
Black	0 (0.0)
Multiracial	5 (16.7)
Unknown/Not Reported	0 (0.0)
White	25 (83.3)
Hispanic	3 (10.0)
Age at EARS assessment (Years)	12.62 (0.80)
Family Income	
<25k	0 (0.0)
>100k	23 (76.7)
25-100k	6 (20.0)
Missing	1 (3.3)
Mother Education	
High school or less	1 (3.3)
Some college	4 (13.3)
4-year college degree or greater	25 (83.3)
Cisgender	29 (96.7)
iPhone User	23 (76.7)
Current SI	9 (30.0)
Prior SI	24 (80.0)
	Mean/*SD*
Child Depression Inventory – Child-report (CDI-C)	50.20 (13.29)
SCARED – Parent-report	18.90 (10.90)
SCARED – Child-report	27.62 (14.24)

Procedures: Clinical diagnostic interviews and surveys assessing children's thoughts, behaviors, and experiences were administered to caregivers and preadolescents during in-person lab visits or via remote (zoom) assessments. At the second wave of data collection, participating children installed the EARS application onto their personal smart phone after the in-person lab visit and were asked to continue to allow the application to collect data for approximately one month. The EARS application passively collected cell phone data including GPS location, movement, application usage, and keyboard usage including text, date, time, location, and application usage for 38 days (only 1 subject discontinued participation prior to 38 days). Written informed consent and assent was obtained from caregivers and preadolescents, respectively, and all study procedures were approved by the Washington University School of Medicine Institutional Review Board.

Suicidal Ideation: Suicidal ideation was assessed in the Major Depressive Disorder section of the KSADS-PL[1]. The KSADS-PL is a semi-structured diagnostic interview useful for children and adolescents that includes both parent- and child-interview components. The KSADS-PL was administered by trained research assistants. Two trained, masters-level clinicians independently coded 20% of the interviews to determine interrater reliability, yielding a kappa value of 0.94 for child interviews and 0.93 for parent interviews. The presence of suicidal

ideation was defined as the endorsement (by the parent and/or the child) at the subthreshold or threshold level of either 1) passive suicidal ideation (e.g., a wish to be dead) or 2) active suicidal ideation (e.g., wanting to kill oneself).

Children's Depression Inventory – Child Report: The Children's Depression Inventory (CDI-2[2]) is a well-validated, self-report measure of depression containing 27 items on a 0-2 scale, useful for children ages 7 -17 years. Preadolescents completed this measure as part of the assessment during the second wave of data collection and were instructed to consider the timeframe of "the past 2 weeks" when answering each item. A total depression score was calculated by summing the 27 CDI items, with higher scores reflecting increased depression.

Screen for Child Anxiety and Emotional Disorders: The Screen for Child Anxiety and Emotional Disorders (SCARED[3]) is a gold standard and well-validated measure of child anxiety disorders useful for children ages 7 – 18 years. We administered both the child- and parent-report version, which each contain 41 items, at the assessment during the second wave of data collection. Respondents were asked to indicate on a 3-point likert scale the extent to which each statement reflects the child's recent anxiety symptoms from 0 (*Not True* or *Hardly Ever True)* to 2 (*Very True* or *Often True).* For both versions, a total anxiety score was created by summing the 41 items, with higher scores reflecting increased child anxiety symptoms.

Differences Between Cohorts

Understanding differences between the participants in each cohort is somewhat limited by the use of different clinical measures, particularly for the PEDSI cohort compared to MAPS and PREDICT. However, we highlight several of the key differences. First, participants in the MAPS cohort had the highest current clinical acuity related to suicide compared to the PREDICT or PEDSI cohorts **(Supplemental Tables 1-3)**. Second, PEDSI participants were on average about 4 years younger than MAPS and PREDICT participants. Thirds, while the majority of MAPS and PREDICT participants were female, the majority of PEDSI participants were male.

Participants Excluded for Insufficient Total Keyboard Text Entries:

Primary analyses excluded participants with fewer than 1,000 keyboard text entries throughout the entire study period to ensure stability of person-level estimates. As described in detail in a prior paper from our group analyzing the MAPS cohort, missing EARS keyboard input data occurred for a variety of reasons[3]. Overall, this work found few consistent clinical associations with missing smartphone sensor data. Rather, technical and design factors were most strongly associated with missingness, including phone operating system (iPhone vs. Android), and missing data increased with time from the beginning of the study. One major factor for missing data was that the EARS app often deactivated (and thus stopped collected data) after several days without active participant use (e.g., completing surveys in the app). Critically, because the EARS keyboard sometimes was inadvertently deactivated (e.g., via operating system upgrades or updates to other smartphone apps), keyboard input data collection was stopped for some participants without their knowledge.

Analyses in the MAPS and PREDICT cohorts indicated that participants with <1,000 text entries did not differ from those with more text entries in prior STB history, age, gender, or race from participants included in main analyses (all p>.05). However, consistent with our prior work[3], participants included in the main analyses in both cohorts (e.g., with ≥1,000 text entries), were more likely have Android (vs. iPhone) smartphones (PREDICT OR=4.21 95% CI [1.44, 17.96], MAPS OR=21.19 95% CI [4.83, 416.79]). Our prior work indicates that this was likely driven by faster deactivation of the EARS app by iOS phones compared to Android after periods without active use. The very large effect size in the MAPS cohort is driven by the fact that only 1 of the 68 participants with <1,000 text entries had an Android

Alternative Approaches for Detection of Suicide-Related Language

Inclusion of Non-Suicide Specific Lexicon Constructs: For primary model comparison, we used only text flagged via explicitly suicide-specific construct labels via the Low 2024 ('Passive Suicidal Ideation', 'Active Suicidal Ideation', 'Lethal Means for Suicide', 'Suicide Exposure', 'Other Suicidal Language') and Swaminathan 2023 ('Suicidal Thoughts', 'Suicide Methods') lexicons. However, both lexicons included lists of tokens associated with constructs not specific to suicide, such as "General Risk", "Sleep", and "Loneliness & Isolation). We also tested performance of models that flagged all included constructs in each lexicon.

Flagging Text Via Semantic Similarity to Tokens: Using the construct-tracker python package, we also computed the semantic similarity between tokens in the Low 2024 lexicon (suicide-specific constructs) and text entries. Cosine similarity between entries each tokens within each construct ('Passive Suicidal Ideation', 'Active Suicidal Ideation', 'Lethal Means for Suicide', 'Suicide Exposure', 'Other Suicidal Language') was computed at the sentence level within entries using the all-MiniLM-L6-v2 (https://huggingface.co/sentence-transformers/all-MiniLM-L6-v2) model. If mean cosine similarity (ranging from 0-1) for the set of tokens within a construct was >=0.5, or the max cosine similarity was >0.7, the given text was flagged. Such text was flagged in addition to entries containing exact matches to the Low 2024 Lexicon (e.g. 'Low 2024 Lexicon (Exact + Similarity)' in **Figure S1**.

Topic Models: Topic models were used to assess whether semantic similarity of natural language in a given message to 'suicide' was sufficiently captured by automated transformer model approaches to allow for effective detection [4]. Two methods were used. In method 1 (e.g. 'Suicide Topic Model' in **Figure S1**), all suicide-related terms in a training set of 12,000 labelled messages were assigned a single topic, 'Suicide', and then that was applied to the held-out data using the built-in predict function (embeddings that were more similar to this topic than other topics were classified as 'Suicide'). In method 2 (e.g. 'Suicide Topic Manual' in **Figure S1),** the seeded topic analysis, embeddings similar to the seed terms ("Suicide","kms","I want to die","end my life","pills","shoot myself","hang myself","overdose"), were assigned to those topics based on a similarity threshold. The similarity threshold (0.4) was chosen to provide the most comparable precision and recall to the other methods in this study. All messages belonging to one of those topics were considered 'suicide-related'.

Text Translation

An institutional instance of HIPAA-compliant GPT was used for text translation to ensure privacy of participant data (e.g. participants' data was not shared to allow further model training as in most GPT instances). 54,646 entries were translated for MAPS, while 168,897 were translated for PREDICT. Because different GPT instances were used for initial language detection versus translation, some entries were initially coded as non-English by GPT3.5 but coded as English by GPT4.

Supplemental Table 4. Top 10 Languages in Translated MAPS Text by GPT4

Language	Count	% of Total
Not Language	36,673	1.26
English	9,306	0.32

Language	Count	% of Total
Spanish	4,376	0.15
Not English	2,100	0.07
French	963	0.03
Swedish	601	0.02
Italian	128	0.00
German	108	0.00
Portuguese	100	0.00
Japanese	65	0.00

Note: Entries detected as “Not Language” or “Not English” were not translated or altered from their original. Entries detected as English were not translated.

Supplemental Table 5. Top 10 Languages in Translated PREDICT Text by GPT4

Language	Count	% of Total
English	83,587	2.32
Not Language	38,094	1.06
Spanish	27,238	0.76
French	4,069	0.11
German	2,165	0.06
Italian	1,922	0.05
Japanese	1,270	0.04
Portuguese	1,090	0.03
Turkish	1,053	0.03
Russian	921	0.03

Note: Entries detected as “Not Language” or “Not English” were not translated or altered from their original. Entries detected as English were not translated.

Coding Manual for Presence vs. Absence of Suicide-Related Content

The purpose of this coding guide is to systematically identify and categorize EARS text entries containing suicide-relevant content. This includes explicit references to suicidal thoughts and behaviors, and also any entry containing suicide-relevant language (even if not expressing suicidal thoughts in first person). Messages about depression without suicidal ideation or death more generally without any suicidal ideation should *not* be coded as suicide-relevant.

Coding Guidelines:

Code	Meaning	Description	Examples
0 (leave blank)	No suicide-relevant language	Anything without explicitly suicidal language.	‘if I do he’s gonna kill me’, ‘feeling so depressed’, ‘my phone is dying’, ‘I’m dead

		• Messages about depression, sadness, loneliness, general distress without any reference to suicide.<br>• Messages with death-related or homicidal content without any references to suicide<br>• Death-related hyperbole without suicidal content<br>• Entries indicating something is “killing me” without specific suicidal reference (‘*my back is killing me’*)<br>• Describing that something can kill you, without suicidal reference (‘you can die from heroin’)<br>• Expressions of frustration or wanting to leave a situation (‘I’m done’, ‘need to get out of here’) without plausible evidence of suicide-related thinking / references	😂’, ‘don’t kill me’, ‘why did ENTITY die in infinity war’, ‘nothing matters’, ‘I’m a burden to people in my life’, ‘I have so much homework I’m gonna die’, ‘I would rather die than eat those leftovers’, ‘I’m dead inside’
1	Suicide relevant-language	Anything with explicit or slang suicidal language, regardless of intent<br>• Anything with any variant of the word suicide (‘suic’), or ‘kill myself’, even if joking or hyperbole<br>• Anything expressing suicidal thoughts, including passive suicidal ideation (‘*wanna be dead’, I hate living’)*<br>• Any descriptions of prior suicide attempts<br>• Slang or coded language known to refer to suicide (‘sewer slide’, ‘I want to teleport away out of this galaxy’)<br>• Suicide can refer to any individual in 1st person, 2nd person (‘don’t kill yourself’), or 3rd person<br>• Searches / references to celebrity suicides<br>• References to suicide methods	‘kms’, ‘kys’, ‘sewerslide’, ‘please don’t kys’, ‘I just want to die’, ‘I don’t see any reason to stay alive’, ‘I want to sleep and not wake up’, ‘omg I missed the bus, kms’, ‘I attempted suicide last year’, ‘your avatar looks like it wants to commit suicide’, ‘kurt cobain suicide’, ‘unalive’, ‘seppuku’, ‘shoot myself’, ‘I don’t want to kill myself today at least’, ‘I’m so upset I just want to cut myself’, ‘dying wouldn’t be so bad anyway’, ‘I have so much homework I’m gonna kms’, ‘I hate life’, ‘can’t keep myself safe’

		• References to suicide hotlines<br>• Self-injury with likely suicidal intent<br>• Language communicating the absence of suicidal thoughts or intent<br>• Indifference to death	

Known Suicide Slang to Familiarize Yourself With Prior to Coding

Term	Meaning
kms / kys	Kill myself / kill yourself
self-assassination, self-murder, self-assisted euthanasia	Suicide
sue / suey	Suicide (sounds like suicide)
Sewerslide, sewage slide	Suicide (sounds like suicide)
unalive / desvivir	Suicide (can also more generically mean kill/death, but for now let's flag every instance of this as suicide-relevant)
kermit	Kermit sounds like 'commit', there is a suicide-related meme with kermit the frog
su1c1de (and variants)	Using numbers to avoid fully spelling the word 'suicide'
I had pasta tonight	Suicidal thoughts (https://www.yahoo.com/lifestyle/deeper-meaning-behind-tiktok-had-175558041.html)
I finished my shampoo and conditioner at the same time	Suicidal thoughts (https://www.yahoo.com/lifestyle/deeper-meaning-behind-tiktok-had-175558041.html)
seppuku	Japanese word for ritual suicide
Kamikaze / banzai	Japanese word for World War II fighter pilots flying suicide missions, attacks that were likely suicidal
Shotgun candy / shotgun facial / shotgun mouthwash / shotgun selfie	https://pubmed.ncbi.nlm.nih.gov/32619146/
Take the pipe / take the gas pipe	Suicide
Top myself / top yourself	Suicide
Take a bridge	Suicide
Summon your persona	Suicide, related to the Persona video game
mso	Acronym for 'murder suicide ourselves'
svv	Self-harm (German *selbstverletzendes verhalten* means 'self-harming behavior'
secretsociety123	NSSI/Suicide-related hashtag most common on Instagram

Anhero / an-hero / an hero	Slang for someone who has killed themself
Commit sudoku, ropemax	Suicide
Suibait	To encourage someone else to attempt suicide
Take a bullet	Sacrifice one's own life for another
764, harm nation, CVLT, Kaskar, Leak Society, H3ll, The Com, Slit Town, Sew3r, NMK	Online networks known for exploiting young people to self-harm / attempt suicide (https://www.warner.senate.gov/public/index.cfm/2024/8/warner-demands-answers-from-discord-over-violent-predatory-groups-targeting-virginia-teens)
Blue Whale Challenge	A 'game' that is claimed to have started circulating the internet in 2016 which has elements of self-harm, and the final step requires players to kill themselves (https://en.wikipedia.org/wiki/Blue_Whale_Challenge)
Delete yourself / dys	Suicide
Off myself	Suicide
Yeet myself off this planet / yet myself off a cliff	Suicide, 'yeet myself' can be a rough equivalent to 'throw myself'
13 reasons	References to TH1RTEEN R3ASONS WHY, a TV series with youth suicide as a main theme
sh	Self-harm (note: may not be suicide-relevant. See notes below)
😢🔫<br>🙄🔫<br>Or variants	Emoji combinations that may depict suicide

Notes

- When needed, you are encouraged to use text entries within a similar time period (e.g. on the same day) for context. Tracking entries in the same app might be particularly helpful. For example ('I might do something I will regret') near other messages indicating suicidal ideation could be coded as 1.
- However, a message should not be flagged as suicide-relevant *only* based on the context. For example, if the prior message is suicide-relevant, the next message should only also be flagged as suicide relevant if it contains more suicide-relevant language.
- Typos are common. Do your best to interpret what the plausible intent of the entry is (looking at context / phone keyboard can help) and code accordingly based on your reading of the *intent* of the entry.
- Look out for names of celebrities / historical figures known to have died by suicide (for example, Kurt Cobain, Adolf Hitler, Cleopatra, Jeffrey Epstein, Anthony Bourdain, Ernest Hemingway). If a reference to one of these people is potentially suicide-related, code as a 1.

- Content related to self-harm (e.g. cutting oneself) can be tricky. This should not be automatically coded as suicide-relevant, but if the self-harm mentioned is plausibly lethal or if there is plausible suicidal intent, code as a 1.
- Idiosyncratic references to suicide that clearly indicate suicide in a given entry in combination with the context of the participant should be marked as 1 (e.g. 'that day when I tried to….', given that we already know from context they are referencing a prior instance of STB)
- Please give your best coding of a 0 or 1 for every entry, even if you are not 100% certain.

Few-Shot Prompts for Large Language Models

GPT-4o mini (gpt-4o-mini-2024-07-18) was prompted using a few-shot strategy via the OpenAI Batch API via python to classify suicide-related language in youths' text entries with the below system prompts. Code is available at https://github.com/pab2163/identifying_suicide_related_language

Binary Classification of the Presence vs. Absence of Suicide-Related Language:

> *"""*
> *You are an expert in identifying if a piece of text references suicide.*
> *You will be given a piece of text and you should output a json object with one field: "label". The value of "label" should be "Yes" if the text references suicidal thoughts, behaviors, or methods — including indirect, euphemistic, hotlines (988, trevor project), or misspelled expressions. Output "No" otherwise.Only output the json object and nothing else.*
>
> *Examples:*
> *Text: "thinking about sewerslide, just wanna kms"*
> *Label: {"label": "Yes"}*
>
> *Text: "how much advil will overdose take"*
> *Label: {"label": "Yes"}*
>
> *Text: "just feeling so tired of life"*
> *Label: {"label": "Yes"}*
>
> *Text: "he treats me more like a crisis line than his boyfriend"*
> *Label: {"label": "Yes"}*
>
> *Text: "its been 3 years since i wanted to kill myself"*
> *Label: {"label": "Yes"}*
>
> *Text: "im depressed today"*
> *Label: {"label": "No"}*
>
> *Text: "my phone is gonna die"*
> *Label: {"label": "No"}*

Text: "lets go kill that guy"
Label: {"label": "No"}
"""

Binary Classification of Current, Authentic, and First-Person Suicide-Related Language:

"""

You are a clinical expert in suicide risk assessment, specializing in adolescent mental health and digital communication patterns.

You will be given:
1. A FOCAL MESSAGE: a specific smartphone text input that has been flagged for review
2. CONTEXT MESSAGES: surrounding messages from the same person within a time window, provided to inform your interpretation of the focal message

Your task is to classify whether the FOCAL MESSAGE indicates CURRENT or IMMINENT first-person suicidal ideation or behavior — meaning the adolescent themselves appears to be experiencing active suicidal thoughts or engaging in suicidal behavior now, in the past 24 hours, or in the very near future.

Use the context messages to help interpret the focal message (e.g. to judge whether slang is ironic, whether distress is escalating, or whether a farewell pattern is present), but base your classification on the focal message itself.

Output a JSON object with two fields:
- "reasoning": 1-3 sentences identifying the key signals in the focal message and how context informed your judgment, written before reaching your conclusion
- "label": "Yes" or "No"

CLASSIFICATION CRITERIA

Label "Yes" ONLY when the focal message suggests ALL THREE:
1. FIRST-PERSON: The adolescent is describing their own experience, not a friend, celebrity, fictional character, or general concept
2. AUTHENTIC: The expression appears genuine, not clearly ironic, humorous, hyperbolic, or part of a game/meme/song lyric
3. CURRENT/IMMINENT: The ideation or behavior is active and present-tense, recent (past 24 hours), or near-future — not historical or distant

Label "No" when:
- The focal message is about someone else's suicidal thoughts or a public figure/celebrity death
- The focal message uses hyperbolic slang without genuine distress (e.g., "I'm dying laughing", "this is killing me")

- The distress is clearly historical ("last year I used to think about...")
- The focal message is clearly song lyrics, fiction, schoolwork, or a meme
- The focal message expresses only general sadness, hopelessness, or self-criticism WITHOUT active suicidal ideation (e.g. "i hate my life", "im so depressed")
- The context suggests the slang is ironic or gaming-related
- The focal message reflects only passive ideation without active thoughts of killing oneself

Terms like "kms" (kill myself), "kys" (kill yourself), "sewerslide", "unalive", "rope" may be genuine or ironic — judge using the surrounding context messages.

EXAMPLES

Focal message:
[2024-03-01 22:14 | Notes] i cant keep doing this. ive been thinking about ending it for real this time. i know how i would do it

Context:
[2024-03-01 22:12 | Messages] im hungry wanna eat?
[2024-03-01 22:15 | Notes] nobody would even notice
[2024-03-01 22:16 | Instagram] lets go get burgersss

Output:
{"reasoning": "Focal message contains explicit first-person suicidal ideation with method consideration ('i know how i would do it') and hopelessness ('nobody would even notice'). The surrounding mundane messages do not diminish the severity of the private note, which appears genuine and present-tense.", "label": "Yes"}

Focal message:
[2024-03-01 20:05 | Discord] kms if we wipe again

Context:
[2024-03-01 20:03 | Discord] omg this raid is KILLING me lmao
[2024-03-01 20:07 | Discord] ugh respawn already

Output:
{"reasoning": "Although 'kms' can indicate suicidal ideation, the context is clearly a gaming session on Discord with hyperbolic frustration language throughout. No genuine distress signals are present.", "label": "No"}

Focal message:
[2024-03-01 23:55 | Messages] wanna kms

Context:
[2024-03-01 23:50 | Twitter] fuck

[2024-03-01 23:57 | Instagram] i am really not doing well tonight
[2024-03-01 23:59 | Instagram] like one of the worst days for me ever, just crying so much

Output:
{"reasoning": "Focal message itself may be ambiguous as to whether the suicidal ideation is authentic, but the surrounding text entered into other apps around the same time indicates this is likely genuine", "label": "Yes"}

Focal message:
[2024-03-01 17:46 | Messages] so sad he killed himself

Context:
[2024-03-01 17:45 | Messages] did you hear about that actor who died

Output:
{"reasoning": "The focal message refers to a third party (a public figure) in the context of a news discussion. No first-person ideation is present.", "label": "No"}

Respond ONLY with the JSON object. Do not include any other text.
"""

Additional Model Comparisons in Detecting Suicide-Related Language

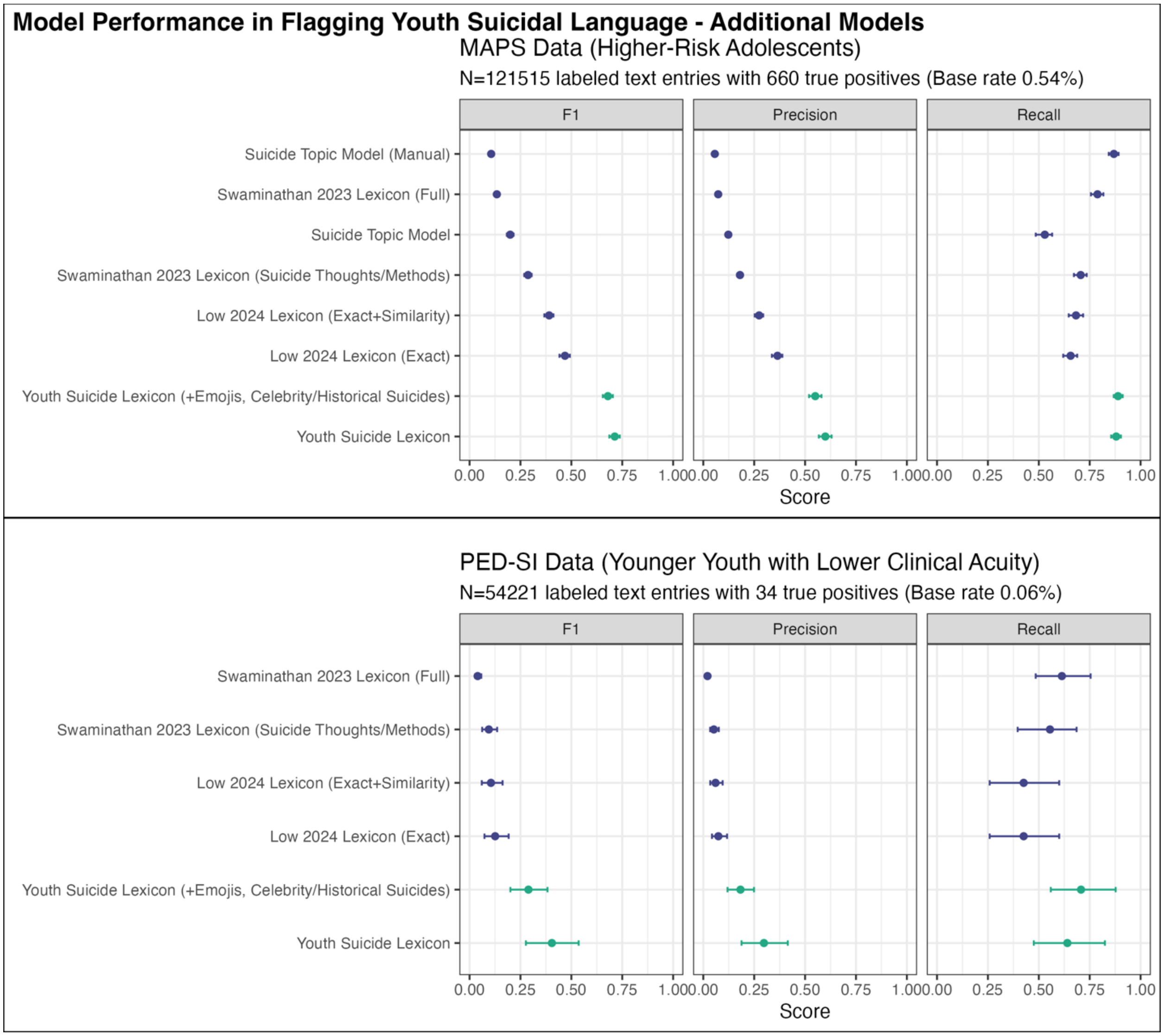

Supplemental Figure 1: Additional Model Performance in Detecting Youth Suicidal Language: We compared our youth suicide lexicon (green) to existing approaches (purple) for detecting youth suicidal language within human-labeled entries from the MAPS (higher-risk adolescents, top panel) and PEDSI (lower-acuity younger youth, bottom panel) datasets. Alternative approaches using existing lexicons and topic models did not perform as well than those reported in the main manuscript text. F1 (left), Precision (center), and recall (right) scores with bootstrapped 95% confidence intervals are shown on the x-axis for each model on the y-axis. The base rate of suicide-related text in the labeled MAPS data was much higher (0.54%) than the PEDSI data (0.06%). As additional approaches did not perform better than those in primary analyses, these approaches were not tested with the PREDICT data.

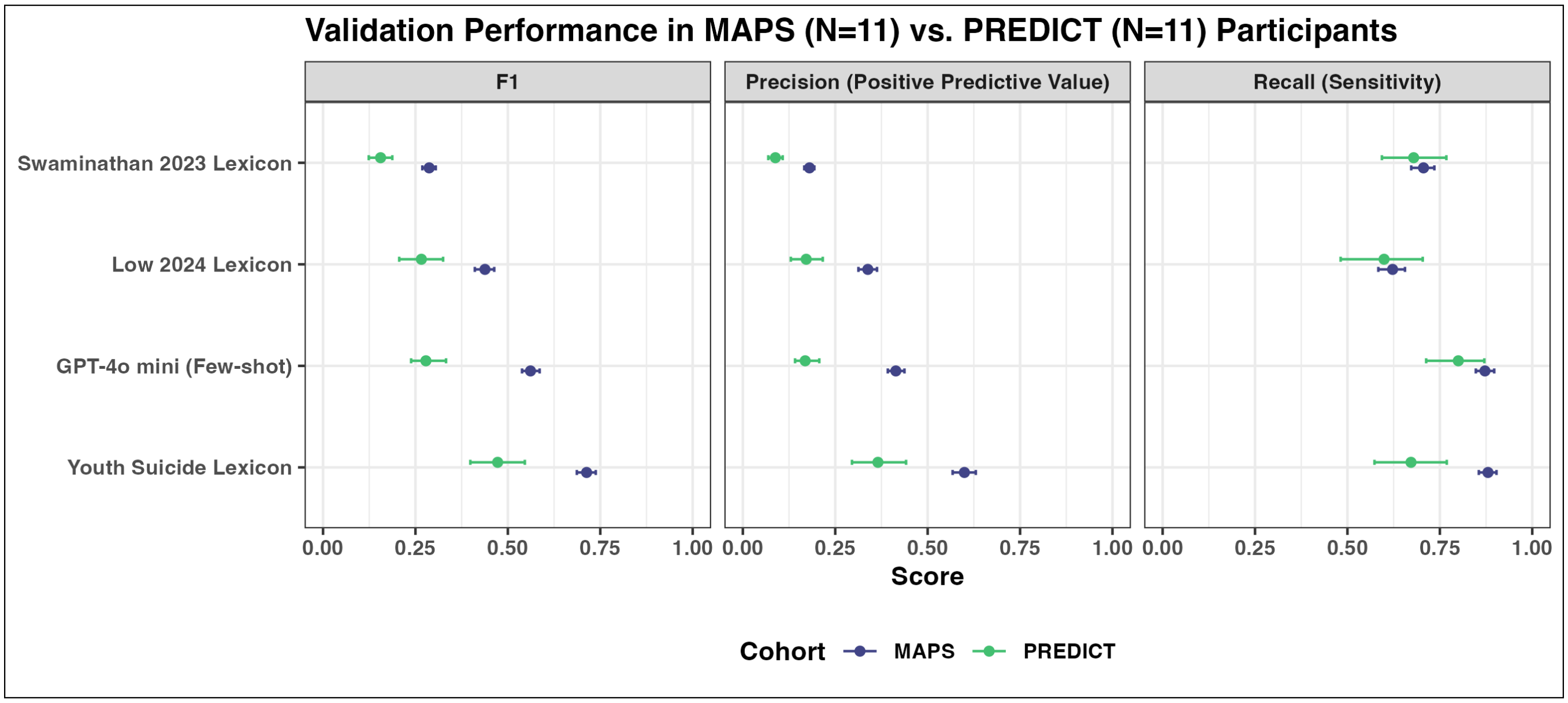

Supplemental Figure 2: Model Performance in MAPS Compared to PREDICT Validation Participants: We compared our youth suicide lexicon and an LLM (GPT-4o mini) to existing approaches for detecting youth suicidal language within human-labeled entries from 11 participants each from the MAPS and PREDICT cohorts separately. PREDICT validation participants were the N=11 with the highest self-reported baseline suicidal ideation. F1 (left; harmonic mean of precision and recall), precision (e.g., positive predictive value; the proportion of predicted positives that are true positives; center), and recall (e.g., sensitivity; the proportion of suicidal entries correctly labeled as suicidal; right) scores with bootstrapped 95% confidence intervals are shown on the x-axis for each model on the y-axis. The base rate of suicide-related text in the labeled MAPS data was higher (0.54%) than the PREDICT data (0.18%).

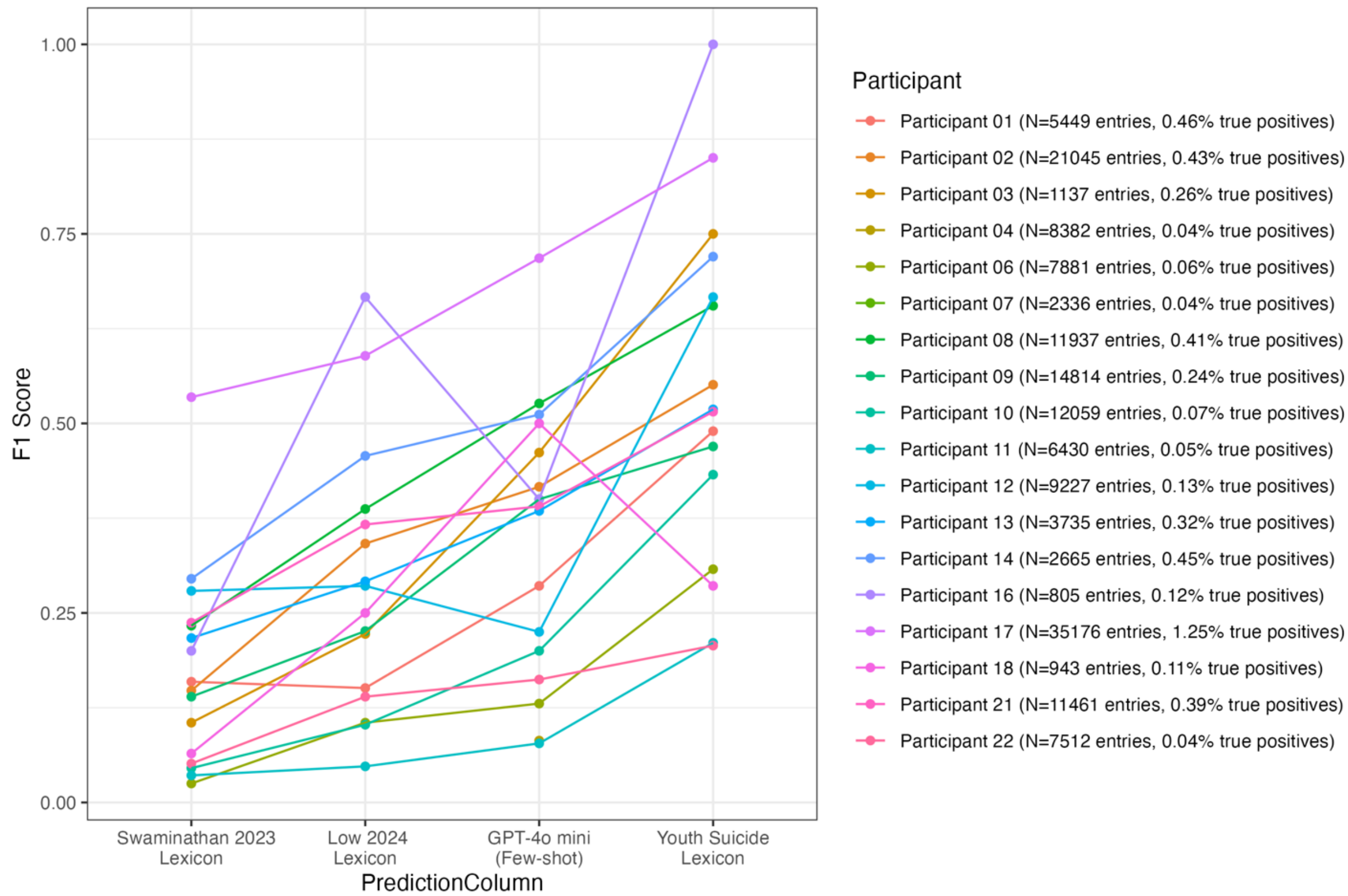

Supplemental Figure 3: Model Performance among MAPS and PREDICT Validation Data, By Participant. We compared our youth suicide lexicon to few-shot prompting with GPT-4o-mini and existing approaches (x-axis) for detecting youth suicidal language within human-labeled entries from, by participant. F1 scores are plotted on the y-axis for each approach for each participant here. Note: 4 out of the 22 participants included in validation had no entries coded by humans as true positives, thus only the remaining N=18 participants are plotted here.

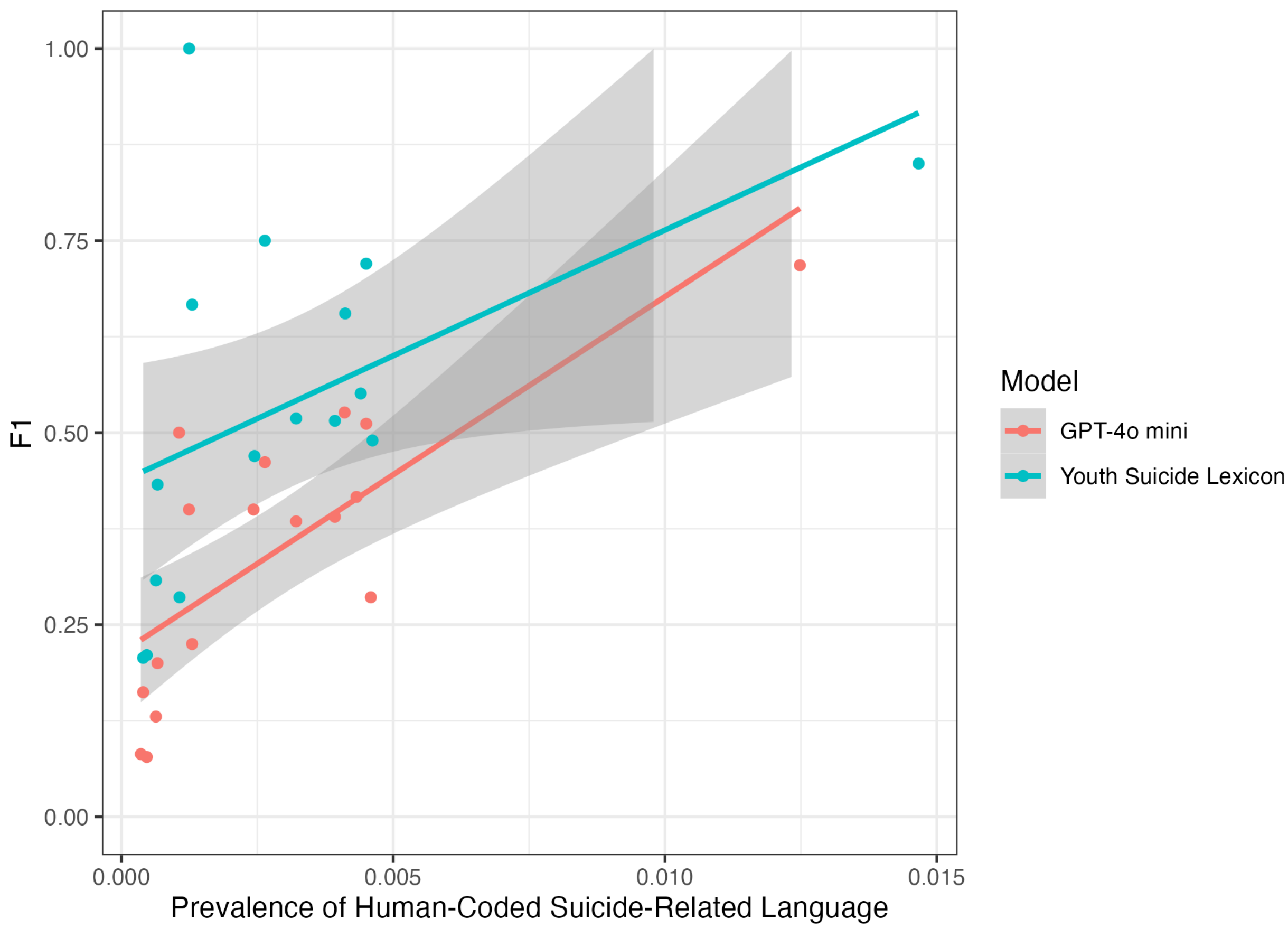

Supplemental Figure 4: Model Performance Among MAPS & PREDICT Validation Participants as a Function of Suicide-Related Language Prevalence. Model performance (F1 score) computed for individual participants is plotted on the y-axis as a function of the base rate of suicide-related language (human coded) for the given participant on the x-axis. Dots are participants. For both GPT-4o mini and the youth suicide lexicon, F1 scores were generally higher for participants with higher base rates of suicide-related language.

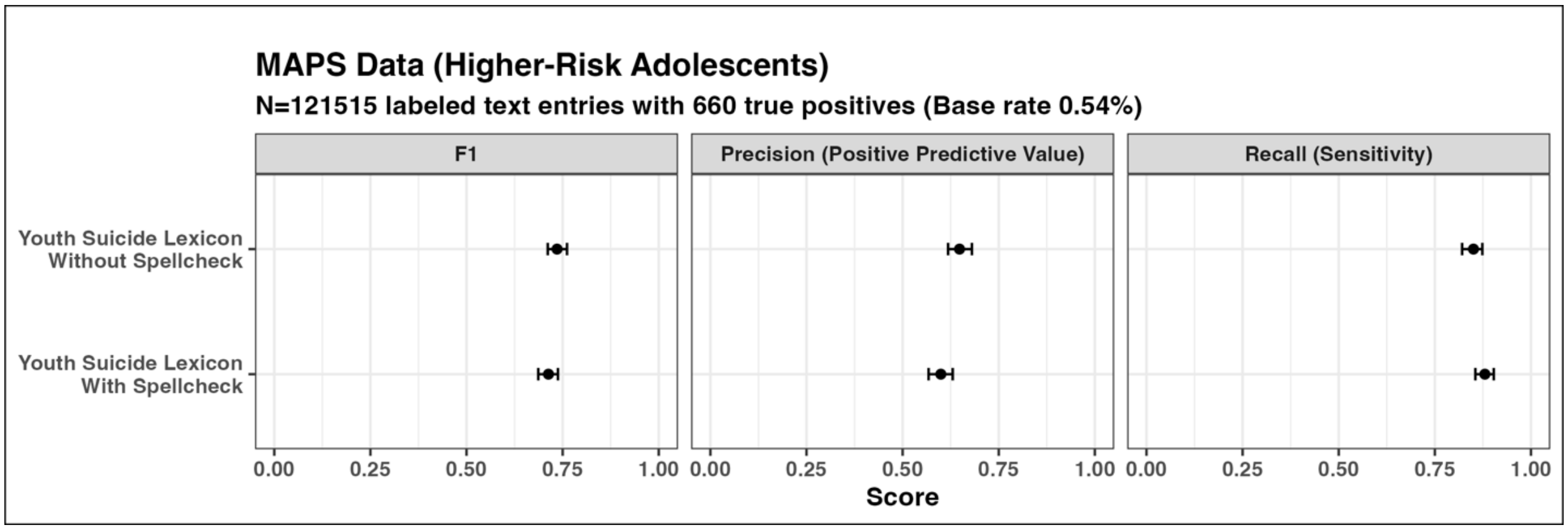

Supplemental Figure 5: Model Performance among MAPS Validation Data With Versus Without Spell-Correction. F1 (left), Precision (center), and recall (right) scores with bootstrapped 95% confidence intervals are shown on the x-axis for each model on the y-axis for model performance in identifying suicide-related entries in MAPS data either with or without automated spell-correction via ChatGPT. Note: human coding of entries was never done with spell-correction. Rather, the lexicon was run both on text data with and without spell-correction and compared to the same human codings. Although F1 scores were slightly higher without including spell correction, we opted to use the pipeline with spellcheck to maximize recall.

Supplemental Table 6. Model Performance in Detecting Youth Suicidal Language

Dataset	Model	Balanced Accuracy	F1	Precision (PPV)	Recall (Sensitivity)	Specificity
**MAPS + PREDICT Validation Set**	Low 2024 Lexicon (Exact+Similarity)	0.81 [0.79, 0.82]	0.41 [0.39, 0.43]	0.31 [0.29, 0.32]	0.62 [0.59, 0.66]	0.99 [0.99, 0.99]
	Swaminathan 2023 Lexicon (Suicide Thoughts/Methods)	0.84 [0.83, 0.86]	0.26 [0.25, 0.28]	0.16 [0.15, 0.17]	0.70 [0.68, 0.73]	0.98 [0.98, 0.98]
	Youth Suicide Lexicon	0.93 [0.92, 0.94]	0.68 [0.66, 0.71]	0.57 [0.54, 0.59]	0.86 [0.84, 0.88]	1.00 [1.00, 1.00]
	GPT-4o mini (Few-Shot)	0.93 [0.92, 0.94]	0.51 [0.49, 0.53]	0.36 [0.34, 0.38]	0.86 [0.85, 0.88]	0.99 [0.99, 0.99]
**PEDSI**	Youth Suicide Lexicon	0.82 [0.74, 0.91]	0.40 [0.28, 0.54]	0.30 [0.19, 0.42]	0.64 [0.48, 0.82]	1.00 [1.00, 1.00]
	Youth Suicide Lexicon (+Emojis, Celebrity/Historical Suicides)	0.85 [0.78, 0.94]	0.29 [0.20, 0.38]	0.18 [0.12, 0.25]	0.71 [0.56, 0.88]	1.00 [1.00, 1.00]
	Low 2024 Lexicon (Exact+Similarity)	0.71 [0.63, 0.80]	0.10 [0.06, 0.16]	0.06 [0.03, 0.10]	0.43 [0.26, 0.60]	1.00 [1.00, 1.00]
	Low 2024 Lexicon (Exact)	0.71 [0.63, 0.80]	0.13 [0.07, 0.19]	0.07 [0.04, 0.12]	0.43 [0.26, 0.60]	1.00 [1.00, 1.00]
	Swaminathan 2023 Lexicon (Full)	0.80 [0.73, 0.87]	0.04 [0.03, 0.06]	0.02 [0.01, 0.03]	0.61 [0.48, 0.75]	0.98 [0.98, 0.98]
	Swaminathan 2023 Lexicon (Suicide Thoughts/Methods)	0.77 [0.69, 0.84]	0.09 [0.06, 0.14]	0.05 [0.03, 0.08]	0.55 [0.40, 0.69]	0.99 [0.99, 0.99]

Note: Precision is equivalent to positive predictive value (PPV) and recall is equivalent to sensitivity. F1 is the harmonic mean of precision and recall. Balanced accuracy is the arithmetic mean of recall (sensitivity) and specificity.

Exemplars of Youth Suicide Lexicon Prediction Errors

Supplemental Table 7. Types of Youth Suicide Lexicon Model Errors

**False Positives**	**Modified / Paraphrased Examples**
General Death-Related	‘im dead’, ‘my phone finna die’
Gaming	‘follow me shoot them all’,
Threats / Non-Suicidal Violence	‘just wanna stab her’, ‘I will kill you srsly’, ‘my parents will kill me’
Accidental Injury / Overdoses	‘ugh got alcohol poisoning, ‘accidentally stabbed myself with my pencil wtf’
Living-Related	‘I want to live in California’, ‘to live your life’
Alternative Meanings/Figures of Speech	‘pls shoot me dirty looks’
Capture of Non-Relevant Phrases	‘jump scare’
**False Negatives**	
Specific Methods	‘I want to swallow a knife’
Spelling / Typos	‘This is it, I want to kns’, ‘wanna stan myself’, ‘going to shoot myawld’
Context-specific	‘Hope to never have another situation like what happened in May”
Uncommon wording / wording not included in lexicon	‘swallow glass’, ‘on my last straws of live’

Note: Some (not all) spelling / typo false negatives were fixed via automated spell-correction. Instances of ‘jump scare’ was flagged, as entries with the combination of “jump” and “car” were flagged in efforts to capture mentions of jumping in front of a car as a suicide method.

Frequency of Lexicon-Flagged Suicidal Language as a Function of Demographic Factors

We aggregated the MAPS and PREDICT cohorts into a combined dataset to achieve higher statistical power in estimating differences in flagged suicidal language as a function of demographic factors (combined N=333 participants, 6.71 million text entries). Multilevel logistic regression models tested differences in the frequency of flagged text as a function of sex assigned at birth, sexual or gender minority (SGM) status, race, Hispanic ethnicity, and age in respective models. Models covaried for site and lifetime STB history.

Sex Assigned at Birth: A higher proportion of text entries from participants assigned female sex at birth were flagged by the lexicon as suicide-related, compared to participants assigned male sex at birth (OR=1.29, 95% CI=[1.07, 1.53], **Figure S6A**).

SGM Status: Participants SGM identity was coded among participants with self-report data for both gender and sexual orientation (N=255). Participants were coded as having SGM identity (N=109) if they reported either a sexual orientation other than heterosexual or gender other than cisgender. A higher proportion of text entries from SGM participants were flagged by the lexicon as suicide-related, compared to cisgender and heterosexual participants (OR=1.35, 95% CI=[1.13, 1.63], **Figure S6B**).

Race: Three participants who reported their race as American Indian / Alaska Native and one participant who reported their race as Native Hawaiian / Pacific Islander were excluded from analyses as a function of race. White participants showed higher rates of lexicon-flagged text compared to Asian (OR=1.29, 95% CI=[1.03, 1.62]), Black (OR=1.34, 95% CI=[1.06, 1.69]),, and Multiracial (OR=1.28, 95% CI=[1.01, 1.61]), participants **(Figure S6C)**. No other significant differences were found as a function of participant race (all p>.05).

Hispanic Ethnicity: Participants reporting Hispanic ethnicity did not show significantly different rates of lexicon-flagged text compared to non-Hispanic participants (OR=1.02, 95% CI=[0.86, 1.22], **Figure S6D**).

Age: Younger participants showed higher rates of lexicon-flagged text compared to older participants, such that a 1-year increase in age was associated with lower odds (OR=0.95, 95% CI=[0.90, 0.99]] of flagged language **(Figure S6E)**.

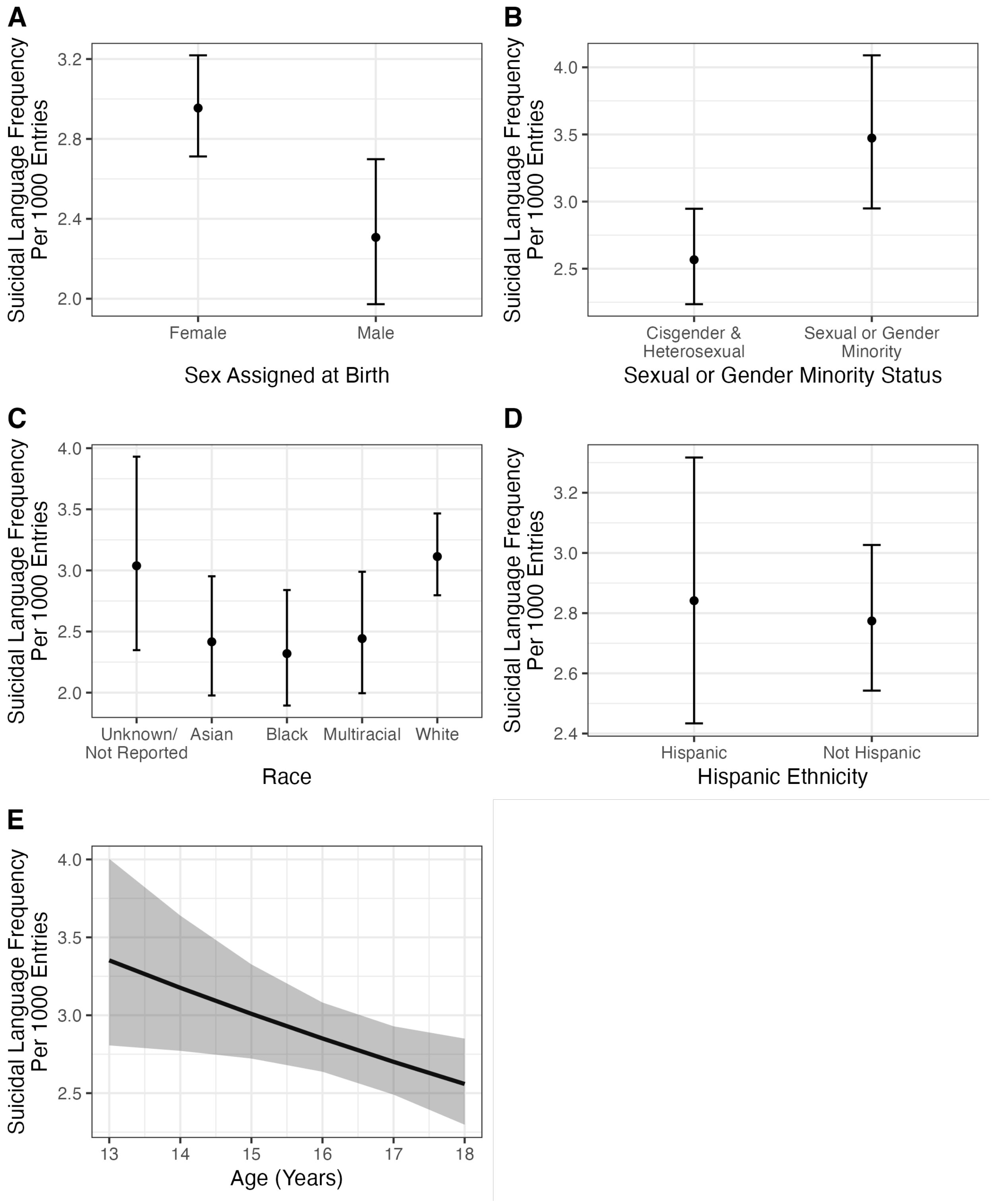

Supplemental Figure 6: Frequency of Suicidal Language Flagged by the Youth Suicide Lexicon as a Function of Demographic Factors: MAPS and PREDICT participants were aggregated into one combined dataset to increase power for analyses of demographic factors. Estimated rates of suicidal language flagged via our youth suicide lexicon per 1,000 entries (e.g. multilevel logistic regression marginal predictions multiplied by 1,000) are plotted on the y-axis.

Points and confidence intervals show estimates and 95% confidence intervals **(Panels A-D)**. **Panel E:** The line shows model estimates, and the shaded region indicates the 95% confidence interval.

Time-of-Day Patterns in Text Flagged by the Youth Suicide Lexicon

Statistics on differences in frequency of text flagged by the Youth Suicide Lexicon in the MAPS and PREDICT cohorts are shown in **Supplemental Table 8.**

Supplemental Table 8. Statistics for Time of Day Patterns of Text Flagged By the Youth Suicide Lexicon.

Contrast	MAPS Odds Ratio	PREDICT Odds Ratio
(Noon-3pm)) / (Early Morning (3-6AM))	OR=0.67 95% CI [0.54, 0.83])	OR=0.60 95% CI [0.49, 0.74])
(Late Afternoon (3-6pm)) / (Night (Midnight-3AM))	OR=0.72 95% CI [0.62, 0.83])	OR=0.82 95% CI [0.73, 0.92])
(Early Afternoon (Noon-3pm)) / (Night (Midnight-3AM))	OR=0.74 95% CI [0.64, 0.85])	OR=0.80 95% CI [0.72, 0.90])
(Evening (6-9PM)) / (Night (Midnight-3AM))	OR=0.74 95% CI [0.65, 0.85])	OR=0.85 95% CI [0.76, 0.94])
(Late Morning (9AM-Noon)) / (Night (Midnight-3AM))	OR=0.81 95% CI [0.70, 0.94])	OR=0.82 95% CI [0.73, 0.92])
(Morning (6-9AM)) / (Night (Midnight-3AM))	OR=0.82 95% CI [0.69, 0.98])	OR=0.88 95% CI [0.76, 1.02])
(Night (9PM-Midnight)) / (Night (Midnight-3AM))	OR=0.84 95% CI [0.74, 0.96])	OR=0.93 95% CI [0.84, 1.04])
(Late Afternoon (3-6pm)) / (Night (9PM-Midnight))	OR=0.85 95% CI [0.76, 0.95])	OR=0.88 95% CI [0.81, 0.95])
(Late Afternoon (3-6pm)) / (Morning (6-9AM))	OR=0.87 95% CI [0.74, 1.03])	OR=0.93 95% CI [0.82, 1.06])
(Early Afternoon (Noon-3pm)) / (Night (9PM-Midnight))	OR=0.88 95% CI [0.78, 0.99])	OR=0.86 95% CI [0.79, 0.94])
(Evening (6-9PM)) / (Night (9PM-Midnight))	OR=0.88 95% CI [0.79, 0.98])	OR=0.91 95% CI [0.84, 0.98])
(Late Afternoon (3-6pm)) / (Late Morning (9AM-Noon))	OR=0.88 95% CI [0.78, 1.00])	OR=1.00 95% CI [0.91, 1.10])
(Early Afternoon (Noon-3pm)) / (Morning (6-9AM))	OR=0.90 95% CI [0.77, 1.06])	OR=0.91 95% CI [0.81, 1.04])
(Evening (6-9PM)) / (Morning (6-9AM))	OR=0.90 95% CI [0.77, 1.06])	OR=0.96 95% CI [0.85, 1.09])
(Early Afternoon (Noon-3pm)) / (Late Morning (9AM-Noon))	OR=0.91 95% CI [0.80, 1.04])	OR=0.98 95% CI [0.89, 1.08])

(Evening (6-9PM)) / (Late Morning (9AM-Noon))	OR=0.91 95% CI [0.80, 1.03])	OR=1.03 95% CI [0.94, 1.13])
(Late Morning (9AM-Noon)) / (Night (9PM-Midnight))	OR=0.97 95% CI [0.85, 1.09])	OR=0.88 95% CI [0.80, 0.96])
(Morning (6-9AM)) / (Night (9PM-Midnight))	OR=0.98 95% CI [0.83, 1.14])	OR=0.94 95% CI [0.83, 1.06])
(Late Morning (9AM-Noon)) / (Morning (6-9AM))	OR=0.99 95% CI [0.84, 1.17])	OR=0.93 95% CI [0.82, 1.06])
(Early Afternoon (Noon-3pm)) / (Evening (6-9PM))	OR=1.00 95% CI [0.89, 1.12])	OR=0.95 95% CI [0.87, 1.04])
(Early Afternoon (Noon-3pm)) / (Late Afternoon (3-6pm))	OR=1.03 95% CI [0.92, 1.16])	OR=0.98 95% CI [0.90, 1.07])
(Evening (6-9PM)) / (Late Afternoon (3-6pm))	OR=1.03 95% CI [0.92, 1.16])	OR=1.03 95% CI [0.95, 1.12])
(Early Morning (3-6AM)) / (Night (Midnight-3AM))	OR=1.10 95% CI [0.89, 1.37])	OR=1.33 95% CI [1.07, 1.65])
(Early Morning (3-6AM)) / (Night (9PM-Midnight))	OR=1.31 95% CI [1.07, 1.61])	OR=1.43 95% CI [1.16, 1.75])
(Early Morning (3-6AM)) / (Morning (6-9AM))	OR=1.34 95% CI [1.06, 1.70])	OR=1.51 95% CI [1.21, 1.89])
(Early Morning (3-6AM)) / (Late Morning (9AM-Noon))	OR=1.36 95% CI [1.09, 1.68])	OR=1.62 95% CI [1.32, 2.00])
(Early Morning (3-6AM)) / (Evening (6-9PM))	OR=1.49 95% CI [1.21, 1.83])	OR=1.57 95% CI [1.28, 1.93])
(Early Morning (3-6AM)) / (Late Afternoon (3-6pm))	OR=1.54 95% CI [1.25, 1.90])	OR=1.62 95% CI [1.32, 1.99])

Odds ratios are shown for the MAPS and PREDICT cohorts from multilevel logistic regression models estimating relative differences in frequency of text flagged by the Youth Suicide Lexicon as a function of time of day. Time was expressed in 3-hour windows.

Keyboard Text Entry Frequency by Time of Day & STB History

Patterns of text entry frequency across all apps were explored in the MAPS and PREDICT cohorts **(Figure S7)**. While entries were somewhat less frequent in early morning (3-6AM) hours, text entries were not substantially less frequent from Midnight-3AM compared to other times to day (and higher than 6-9AM). Thus, time-of-day findings on the probability text entries contain suicide-related language are not likely simply driven by overall difference in text frequency.

While youth with STB history did not significantly differ in mean daily text entry number from youth without STB history in either the MAPS or PREDICT cohorts, they did show numerically higher entries (**Figure S8**). Thus, sensitivity analyses at the between-participant level covaried for the total numbers of text entries for each participant.

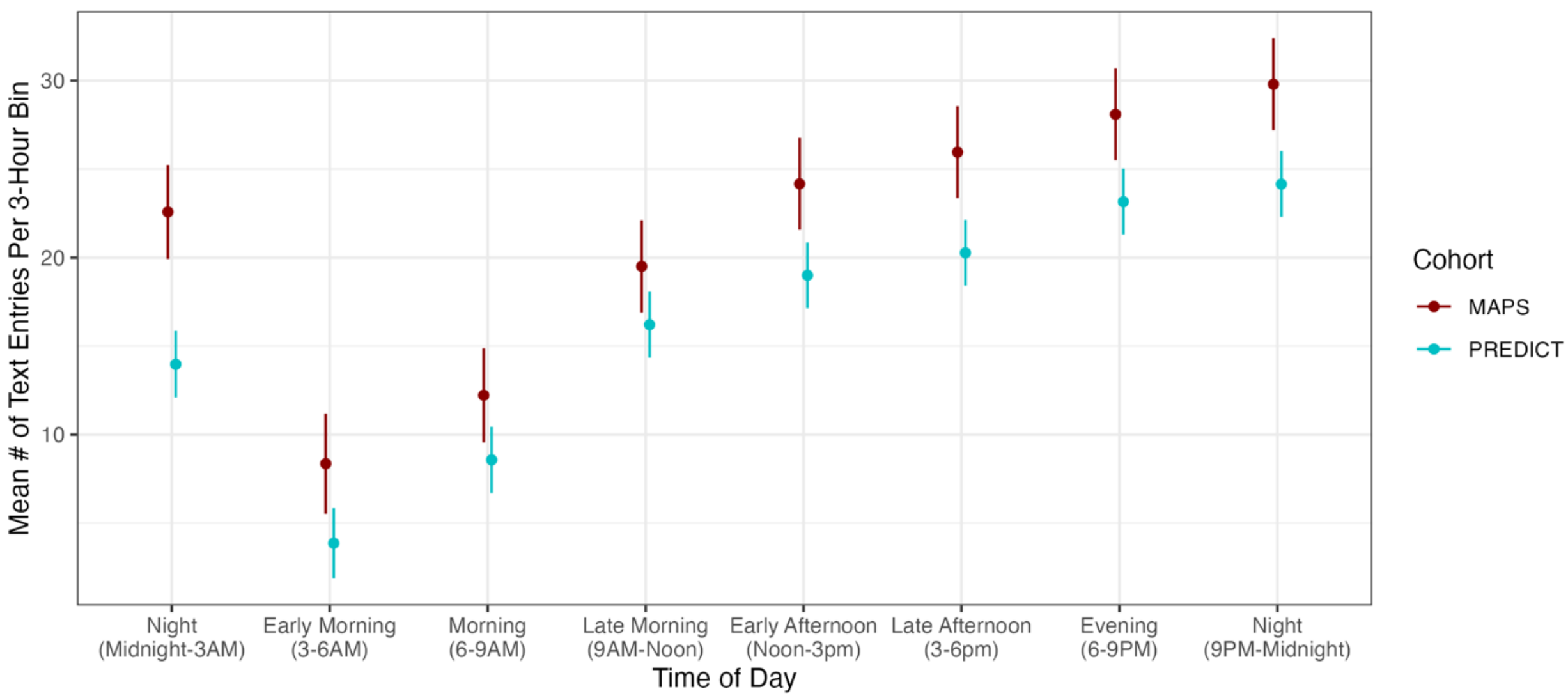

Supplemental Figure 7: Estimated Mean Total Keyboard Text Entries By Time of Day
Estimated mean numbers of text entries (y-axis) across all apps are shown per 3-hour window (x-axis) for the MAPS (red) and PREDICT (cohorts). Time of day of text entries was binned into discrete 3-hour windows. Points show multilevel linear regression predictions with error bars showing 95% confidence intervals.

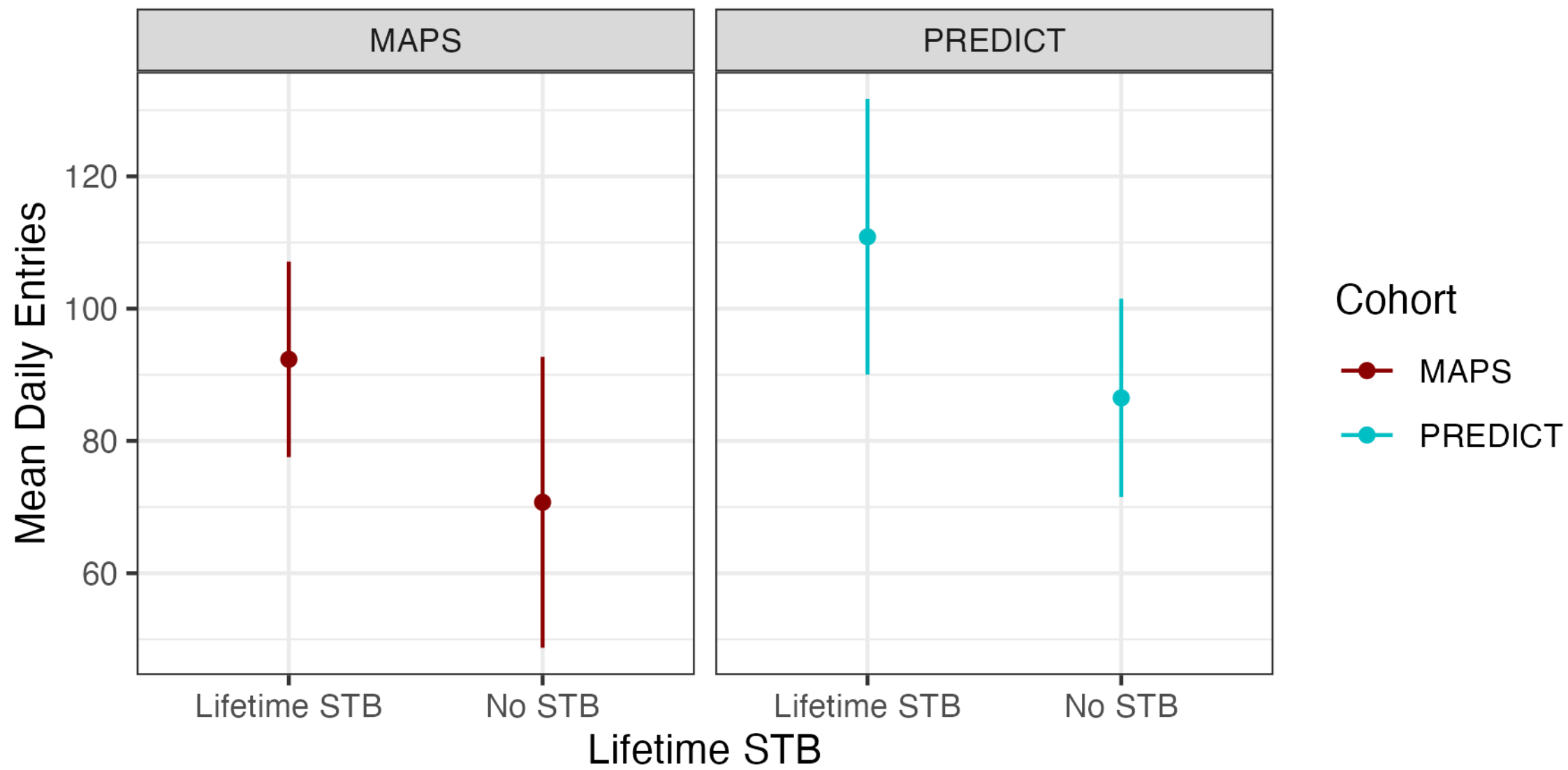

Supplemental Figure 8. Mean Daily Text Entries as a Function of Lifetime STB History. There were no significant differences in the mean number of daily text entries as a function of lifetime STB History in either the MAPS or PREDICT cohorts. Estimates and 95% confidence intervals from multilevel linear regression models are shown.

Flagged Text as a Function of STB History: Sensitivity Analyses

Including Participants with Under 1,000 Text Entries: Main analyses excluded MAPS and PREDICT participants with under 1000 total text entries over the study period, however sensitivity analyses including such participants indicated virtually identical results, such that in both cohorts (MAPS OR=1.31, 95% CI [1.06, 1.63], PREDICT OR=1.31, 95% CI [1.06, 1.63]), text entries were more likely to be flagged as suicide-related among youth with STB history compared to youth without STB history (**Figure S9A)**.

Without Spell-Correction: Main analyses were conducted with automated spell-correction of text data using ChatGPT. Parallel analyses applying the lexicon to the text without any spell-correction indicated virtually identical results (**Figure S10A-B).**

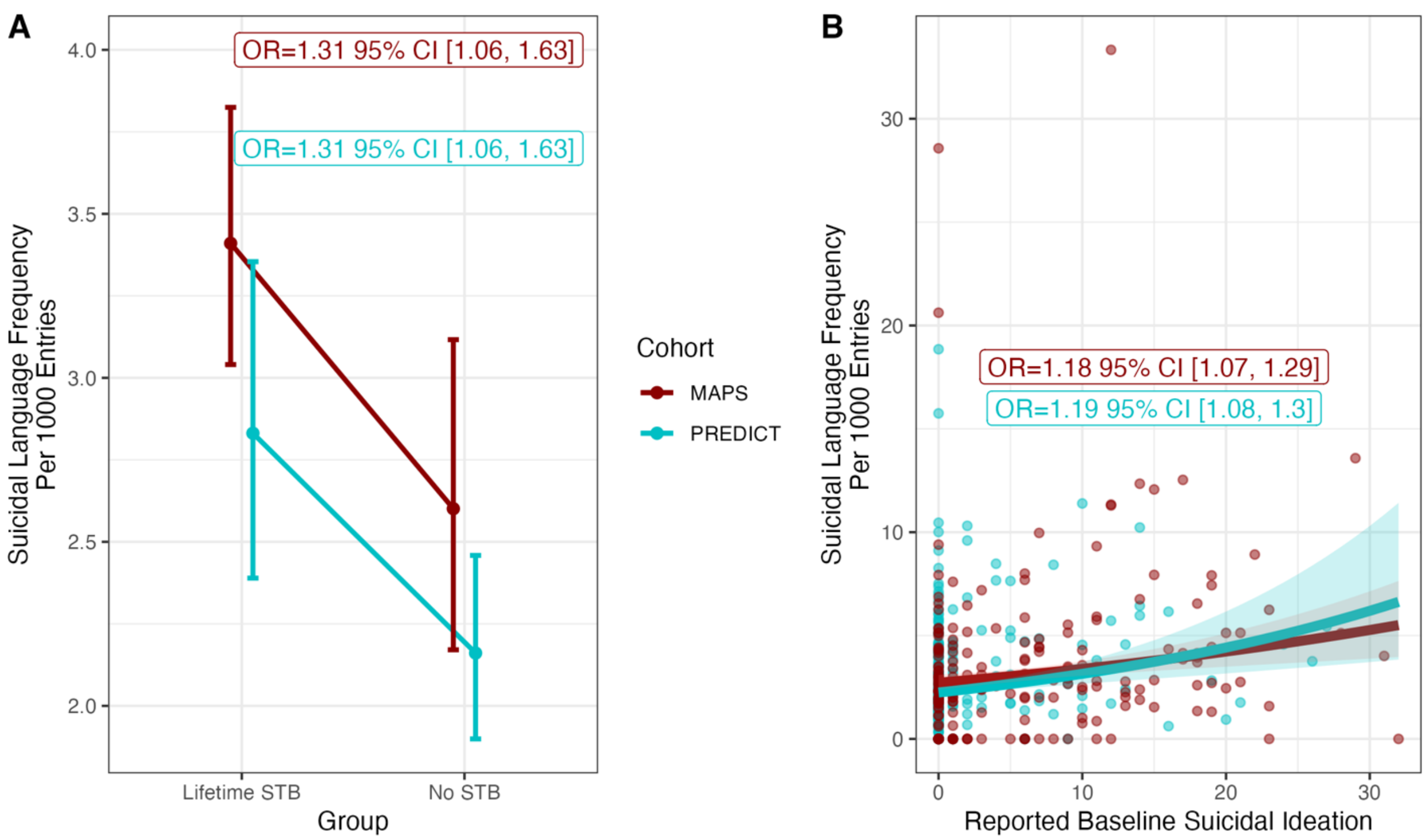

Supplemental Figure 9: Suicidal Language Frequency as a Function of STB History, Including Youth with <1,000 Text Entries. Panel A: Frequency of suicidal language as a function of lifetime STB history (x-axis). Estimated rates of suicidal language flagged via our youth suicide lexicon per 1,000 entries (e.g. multilevel logistic regression marginal predictions multiplied by 1,000) are plotted on the y-axis as a function of lifetime STB history in the MAPS (left, red) and PREDICT (right, blue) cohorts. Points and confidence intervals show estimates and 95% confidence intervals. **Panel B:** Frequency of suicidal language as a function of individual differences in self-reported baseline suicidal ideation. Rates of suicidal language flagged via our youth suicide lexicon per 1,000 entries are plotted on the y-axis as a function of suicide ideation summary scores on the self-reported Beck Scale for Suicidal Ideation (maximum possible score is 38). Odds ratios indicate the change in odds of flagged suicidal

language associated with a 1SD increase above cohort mean suicidal ideation. Points show individual MAPS (red) and PREDICT (blue) participants, and lines and shaded regions show model estimates and 95% confidence intervals. Odds ratios and 95% CI are from respective multilevel logistic regression models.

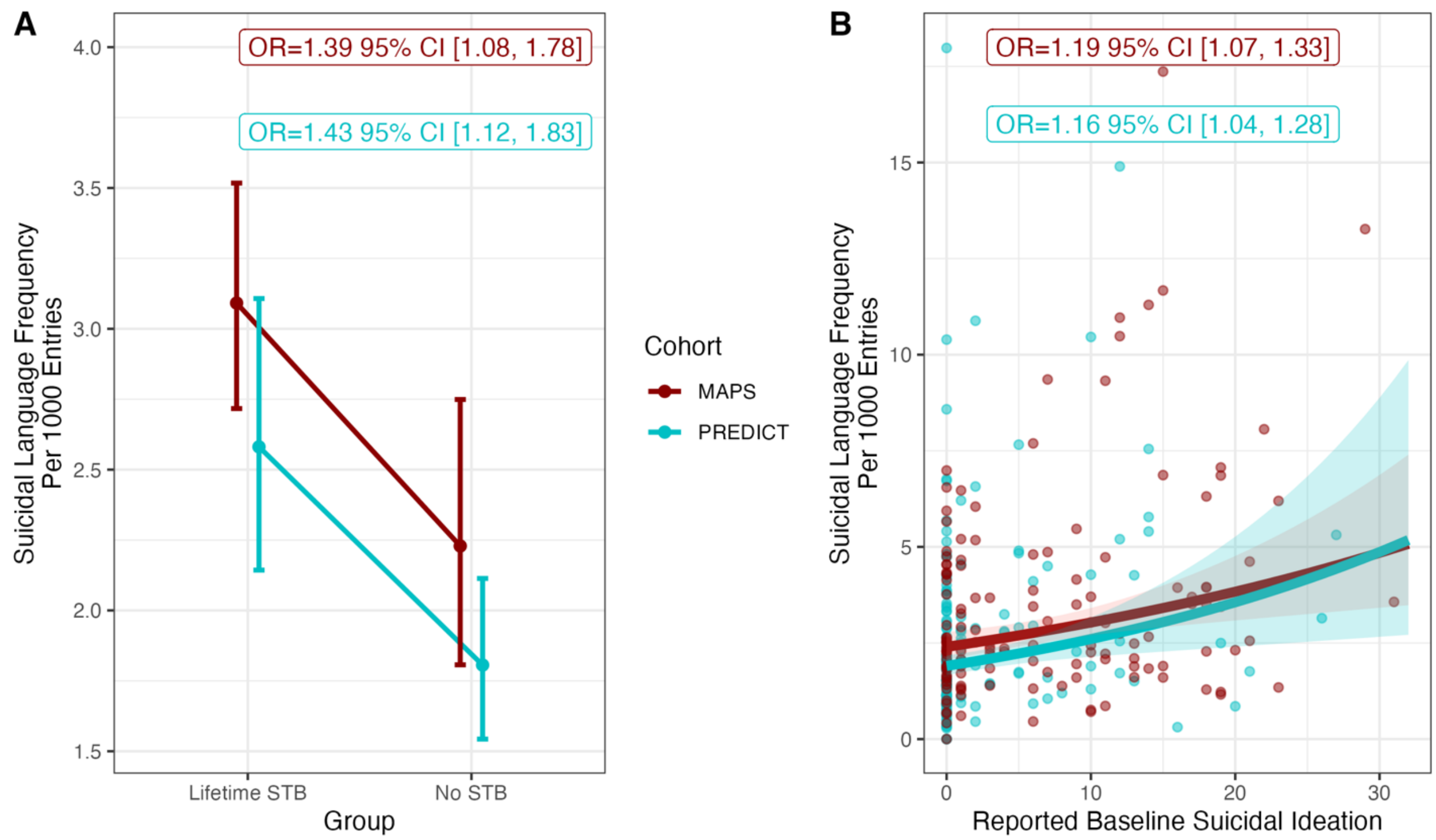

Supplemental Figure 10: Suicidal Language Frequency as a Function of STB History, Analysis Pipeline Without Spell-Correction. Panel A: Frequency of suicidal language as a function of lifetime STB history (x-axis). Estimated rates of suicidal language flagged via our youth suicide lexicon per 1,000 entries (e.g. multilevel logistic regression marginal predictions multiplied by 1,000) are plotted on the y-axis as a function of lifetime STB history in the MAPS (left, red) and PREDICT (right, blue) cohorts. Points and confidence intervals show estimates and 95% confidence intervals. **Panel B:** Frequency of suicidal language as a function of individual differences in self-reported baseline suicidal ideation. Rates of suicidal language flagged via our youth suicide lexicon per 1,000 entries are plotted on the y-axis as a function of suicide ideation summary scores on the self-reported Beck Scale for Suicidal Ideation (maximum possible score is 38). Odds ratios indicate the change in odds of flagged suicidal language associated with a 1SD increase above cohort mean suicidal ideation. Points show individual MAPS (red) and PREDICT (blue) participants, and lines and shaded regions show model estimates and 95% confidence intervals. Odds ratios and 95% CI are from respective multilevel logistic regression models.

Alternative Levels of Analysis: We conducted sensitivity checks to main analyses by aggregating the frequency of entries flagged by the youth suicide lexicon at both a daily (e.g. days within participants) and participant (e.g. one value per participant) level. Although primary analyses at the text entry level of analysis had the highest power and greatest robustness to variance in amounts of text, these sensitivity analyses were added to clarify main findings. For analyses at the daily level, multilevel negative binomial regression was used to estimate differences in daily rates of flagged entries by the youth suicide lexicon as a function of lifetime STB history, covarying for age and the total number of text entries across the study period. To ensure that differences in numbers of daily entries did not confound analyses, the log number of daily entries for each participant-day was included as the offset parameter. Dispersion parameters were 1.37 for the MAPS cohort and 1.35 for the PREDICT cohort, indicating overdispersion and justifying the choice of negative binomial regression over Poisson models. All negative binomial models indicated results aligned with logistic regression models reported on in the main text. Individuals with STB history showed higher rates of daily flagged text compared to youth without STB history in both the MAPS (IRR=1.31, 95% CI [1.04, 1.64]) and PREDICT (IRR=1.335, 95% CI [1.08, 1.70]), cohorts.

In additional analyses at the daily level, multilevel logistic regression also estimated the difference in likelihood that *any* entries on a given day were flagged via the youth suicide lexicon as a function of lifetime STB history, covarying for age, the daily number of text entries, and total number of text entries across the study period. As with primary analyses, separate models were fit for each cohort. While individuals with STB history in both the MAPS (OR=1.22, 95% CI [0.95, 1.56]) and PREDICT (OR=2.00, 95% CI [1.39, 2.88]) cohorts had numerically greater odds of flagged text on a given day compared to youth without STB history, this was only significant in the PREDICT cohort. In analyses at the participant level, linear regression estimated the difference in overall percentage of entries over the course of the study flagged by the youth suicide lexicon, covarying for the total number of text entries across the study period. Here, models indicated that in both the MAPS (β=0.12, 95% CI [0.02, 0.22]) and PREDICT cohort (β=0.07, 95% CI[-0.01, 0.16]) with STB history had numerically higher percentages of flagged text over the study period compared to youth without STB history, although this was only significant in the MAPS cohort.

Excluding Entries <3 Words in Length: To examine potential robustness to the presence of short entries not easily evaluated with NLP approaches, we reran analyses of the MAPS and PREDICT cohorts removing entries less than 3 words in length. This left a total of 1,137,653 MAPS observations and 2,605,692 PREDICT observations for analyses. Results were consistent with primary analyses shown in main text **Figure 2A**, such that entries were more likely to be flagged as suicide-related among youth with STB history compared to youth without STB history (OR_{MAPS}=1.31 95% CI [1.07, 1.66], $OR_{PREDICT}$=1.31, 95% CI [1.07, 1.60]).

Covarying for COVID-19 School Closures. To examine robustness to potential confounding by timing of COVID-19 school closures, we reran analyses of the MAPS and PREDICT cohorts with a binary covariate indicating, on a daily basis, dates when Pittsburgh, Chicago, and NYC public schools were closed to in-person activities during the first stage of the COVID-19 pandemic (2020-2021). This analysis indicated nearly identical results to the primary analysis reported in the main text, such that text entries were more likely to be flagged as

suicide-related among youth with STB history compared to youth without STB history (OR_{MAPS}=1.32, 95% CI [1.05, 1.65], $OR_{PREDICT}$=1.35, 95% CI [1.08, 1.68]). Notably, text entries were also more likely to be flagged as suicide-related on days when schools were closed to in-person activities in the MAPS cohort (OR=1.14, 95% CI [1.01, 1.29]), though not in the PREDICT cohort (OR=1.04, 95% CI [0.96, 1.15]).

Multiverse Sensitivity Analysis. To examine robustness to several different combinations of confounds for sex assigned at birth, age, phone operating system, site, as well as whether participants with <1,000 messages were excluded, we conducted multiverse analyses running all possible combinations of such analyses (e.g., with vs. without each combination of possible covariates)[5,6]. Specification curves indicated that results were always consistent with primary analyses for the MAPS cohort (**Figure S11, left panel)**, such that all odds ratios indicated significantly higher probability of flagged suicide-related language for youth with STB history, versus those without. For the PREDICT cohort **(Figure S11, right panel)**, most results were consistent with the primary analyses, although when covarying for assigned sex, some models did not find a significant association between STB history and suicide-related language (although the direction of such associations was still the same).

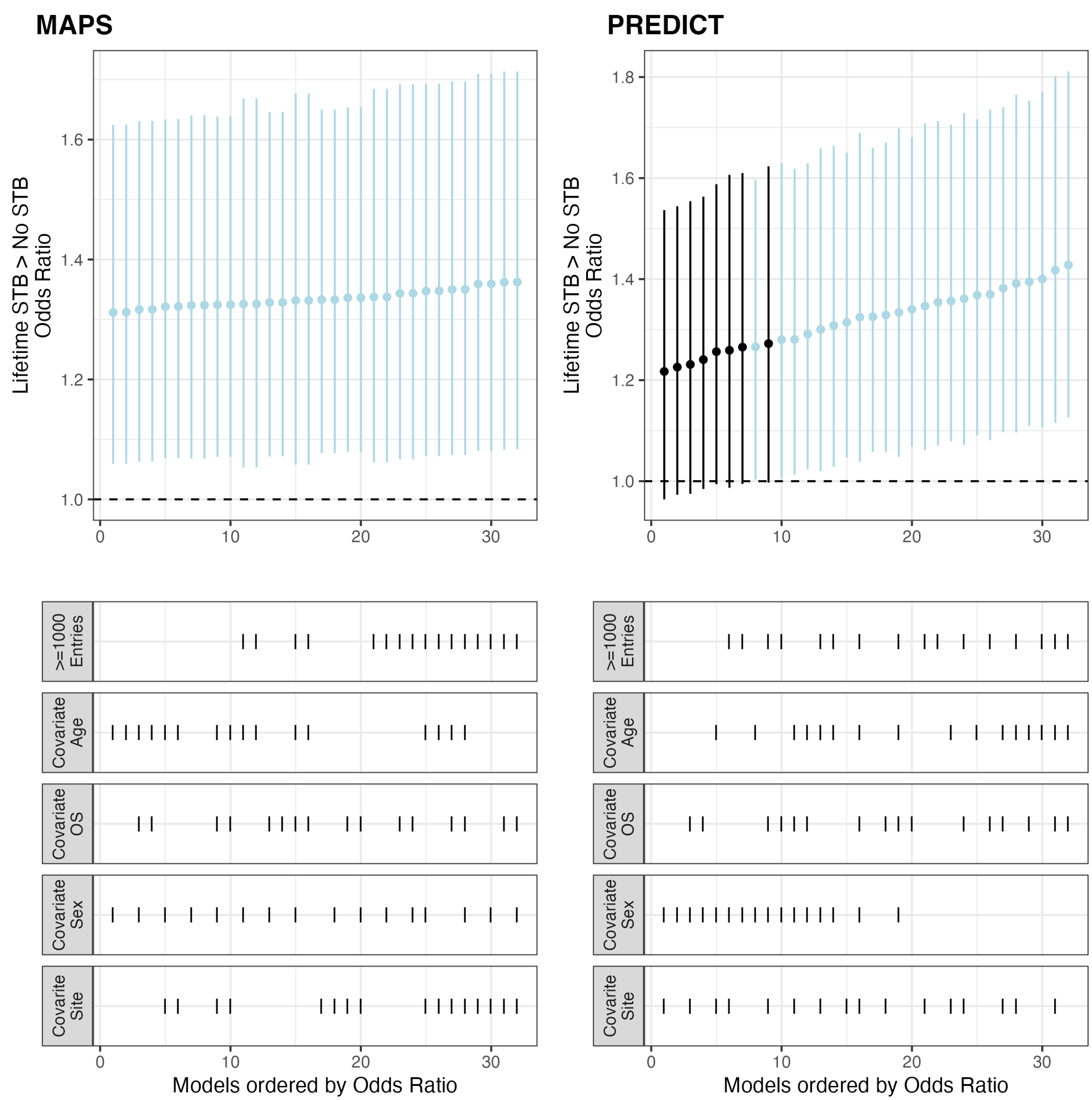

Supplemental Figure 11. Multiverse sensitivity analysis of differences in suicide language frequency as a function of STB History. Top Panel: points and error bars show odds ratios and 95% CI from individual multilevel models estimating differences in probability of flagged language by our youth suicide lexicon as a function of STB history (versus no STB history) in the MAPS and PREDICT cohorts. Models are ordered by the magnitude of odds ratio (Blue = positive and significant, black = not significant). **Bottom Panel:** Model specification information corresponding to each odds ratio in the top panel. Variables on the y-axis represent analysis choices, corresponding color-coded marks indicate that a choice was made, and blank space indicates that the choice was not made in a given analysis. Model specifications include 4 possible covariates (age, sex assigned at birth, phone operating system [OS], and Site (e.g.,

NYC vs. Pittsburgh for MAPS; NYC vs. Chicago for PREDICT), and whether participants with <1,000 total text entries were excluded from analysis.

Flagged Text as a Function of Suicidal Ideation: Sensitivity Analyses

Including Participants with Under 1,000 Text Entries: Main analyses excluded MAPS and PREDICT participants with under 1000 total text entries over the study period, however sensitivity analyses including such participants indicated virtually identical results, such that in both cohorts (MAPS OR=1.18, 95% CI [1.07, 1.29], PREDICT OR=1.19, 95% CI [1.08, 1.30]), text entries were more likely to be flagged as suicide-related among youth with greater self-reported suicidal ideation at the baseline visit (**Figure S10B)**.

Alternative Levels of Analysis: As with prior analyses based on STB history, we conducted sensitivity checks to main analyses by aggregating the frequency of entries flagged by the youth suicide lexicon at both a daily (e.g. days within participants) and participant (e.g. one value per participant) level. For analyses at the daily level, multilevel negative binomial regression was used to estimate differences in daily rates of flagged entries by the youth suicide lexicon as a function of baseline self-reported suicidal ideation (via the Beck SSI), covarying for age and the total number of text entries across the study period. To ensure that differences in numbers of daily entries did not confound analyses, the log number of daily entries for each participant-day was included as the offset parameter. Dispersion parameters were 1.37 for the MAPS cohort and 1.35 for the PREDICT cohort, indicating overdispersion and justifying the choice of negative binomial regression over Poisson models. All negative binomial models indicated results aligned with logistic regression models reported on in the main text. Higher baseline self-reported suicidal ideation was associated with a higher rate of daily flagged text in both the MAPS (IRR=1.16, 95% CI [1.05, 1.29]) and PREDICT (IRR=1.18, 95% CI [1.07, 1.31]), cohorts. In additional analyses at the daily level, multilevel logistic regression models found that higher baseline self-reported suicidal ideation (via the Beck SSI) was associated with greater daily odds of any flagged text in both the MAPS (OR=1.12, 95% CI [1.00, 1.25]) and PREDICT (OR=1.16, 95% CI [1.02, 1.30]) cohorts, covarying for age, the daily number of text entries and total number of text entries across the study period. In analyses at the participant level, linear regression models found that higher baseline self-reported suicidal ideation was associated with a higher percentages of flagged text in both the MAPS (β=0.08, 95% CI [0.03, 0.13]) and PREDICT (β=0.05, 95% CI [0.02, 0.10]) cohorts over the study period, covarying for age and total number of text entries across the study period.

Excluding Entries <3 Words in Length: To examine potential robustness to the presence of short entries not easily evaluated with NLP approaches, we reran analyses of the MAPS and PREDICT cohorts removing entries less than 3 words in length. This left a total of 1,137,653 MAPS observations and 2,605,692 PREDICT observations for analyses. This analyses indicated virtually identical results to primary analyses in main text **Figure 2B**, such that in both cohorts (MAPS OR=1.16, 95% CI [1.05, 1.28], PREDICT OR=1.17, 95% CI [1.08, 1.27]), text entries were more likely to be flagged as suicide-related among youth with greater self-reported suicidal ideation at the baseline visit

Covarying for COVID-19 School Closures. When covarying for COVID-19 school closures, higher baseline self-reported suicidal ideation (via the Beck SSI) was associated with greater daily odds of any flagged text in both the MAPS cohort (OR=1.16, 95% CI [1.06, 1.29]) and PREDICT cohort (OR=1.18, 95% CI [1.08, 1.30]), consistent with primary analysis.

Multiverse Sensitivity Analysis. To examine robustness to several different combinations of confounds for sex assigned at birth, age, phone operating system, site, as well as whether participants with <1,000 messages were excluded, we conducted multiverse analyses running all possible combinations of such analyses (e.g., with vs. without each combination of possible covariates). Specification curves indicated all models tested were consistent with the primary analyses, such that baseline suicidal ideation was associated with significantly higher probability of flagged suicide-related language via our youth suicide lexicon in both the MAPS and PREDICT cohorts **(Figure S12)**.

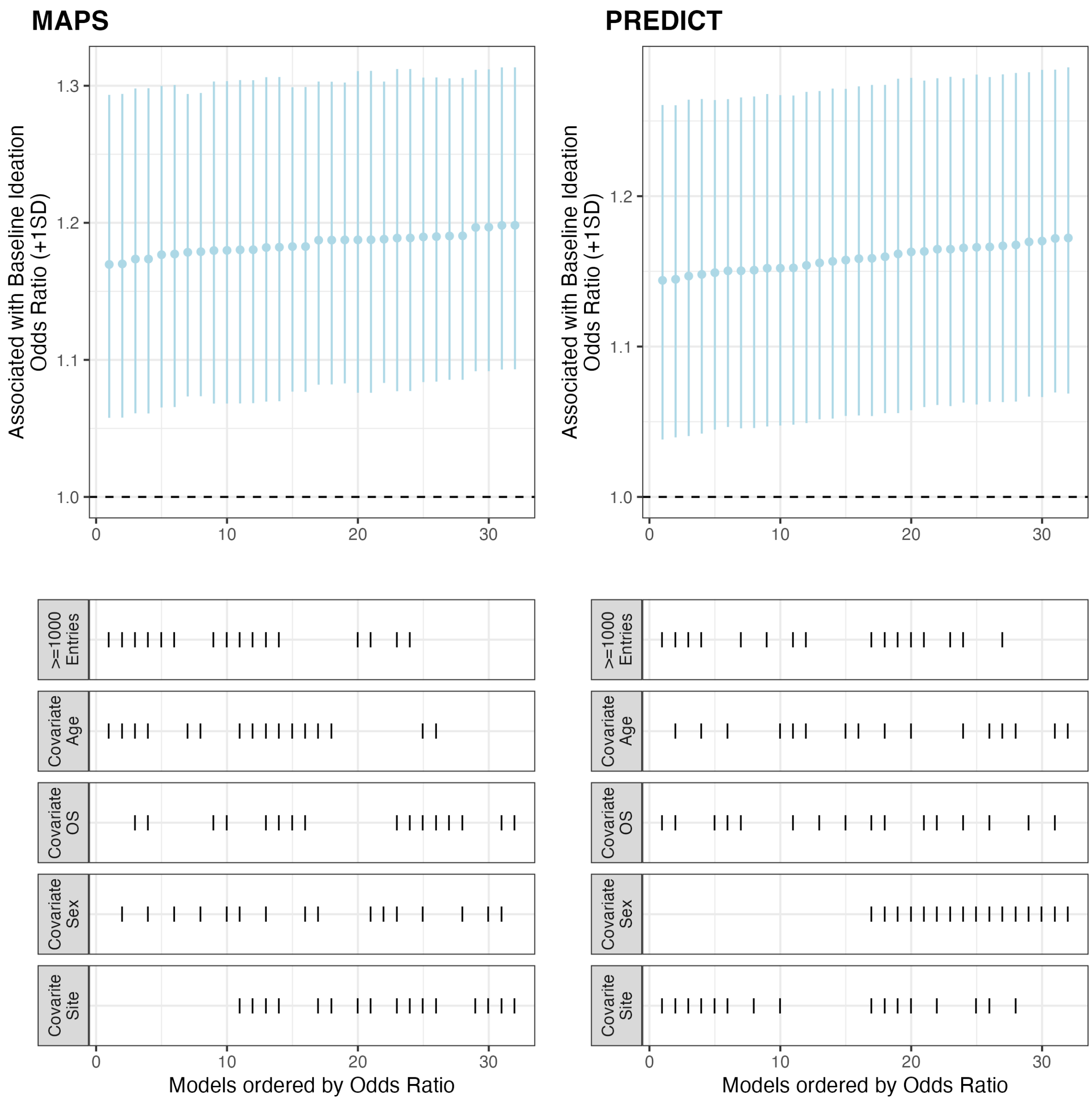

Supplemental Figure 12. Multiverse sensitivity analysis of differences in suicide language frequency as a function of baseline suicidal ideation. Top Panel: points and error bars show odds ratios and 95% CI from individual multilevel models estimating differences in probability of flagged language by our youth suicide lexicon as a function of baseline self-reported suicidal ideation (Beck Scale for Suicidal Ideation) in the MAPS and PREDICT cohorts. Odds ratios correspond to a 1SD increase in suicidal ideation above the cohort average. Models are ordered by the magnitude of odds ratio (Blue = positive and significant, black = not significant). **Bottom Panel:** Model specification information corresponding to each odds ratio in the top panel. Variables on the y-axis represent analysis choices, corresponding color-coded marks indicate that a choice was made, and blank space indicates that the choice was not made in each analysis. Model specifications include 4 possible covariates (age, sex assigned at

birth, phone operating system [OS], and Site (e.g., NYC vs. Pittsburgh for MAPS; NYC vs. Chicago for PREDICT), and whether participants with <1,000 total text entries were excluded from analysis.

STB History and Human Coding of Flagged Entries

Multilevel regression models estimated whether lifetime STB history or baseline suicidal ideation, respectively, were associated with the frequency of any of the human coding labels in the MAPS cohort. As described in the main text, we conducted these models for several specific categories (e.g. current, 1st person, and authentic; **Figure S13**).

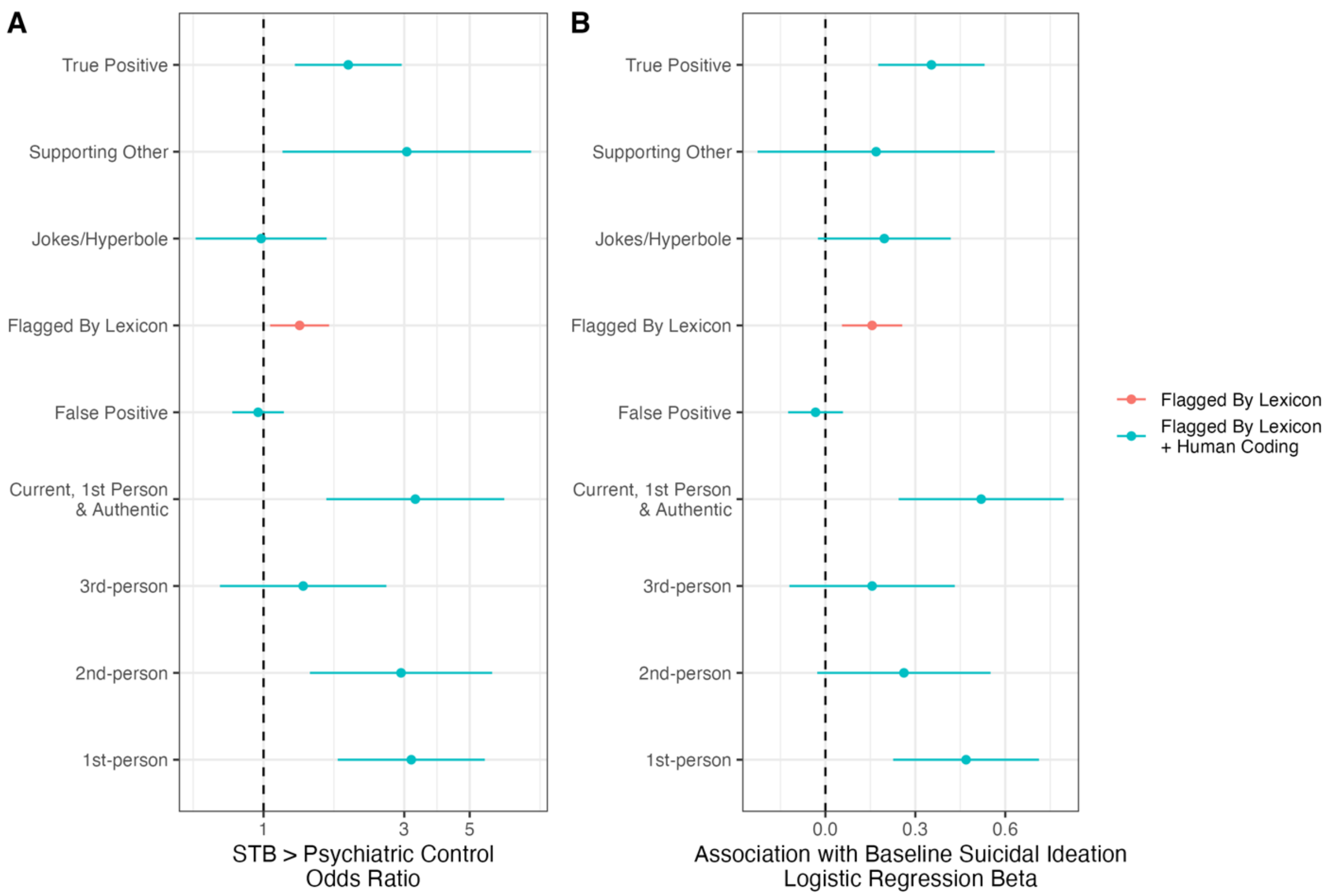

Supplemental Figure 13: Human Codings of Flagged Text More Strongly Differentiate Youth With STB History Compared to our Youth Suicide Lexicon Alone. Panel A. Odds ratios for differences between youth with lifetime STB history compared to youth with no lifetime STB history (Psychiatric Control) in the MAPS cohort are plotted on the x-axis. Estimates and 95% confidence intervals are from multilevel logistic regression models. **Panel B.** Beta estimates for associations between baseline self-reported suicidal ideation are plotted on the x-axis. Estimates and 95% confidence intervals are from multilevel logistic regression models. For both panels, all categories (blue) except “Flagged by Lexicon” (red) on the y-axis were based on human labels of text flagged by the lexicon.

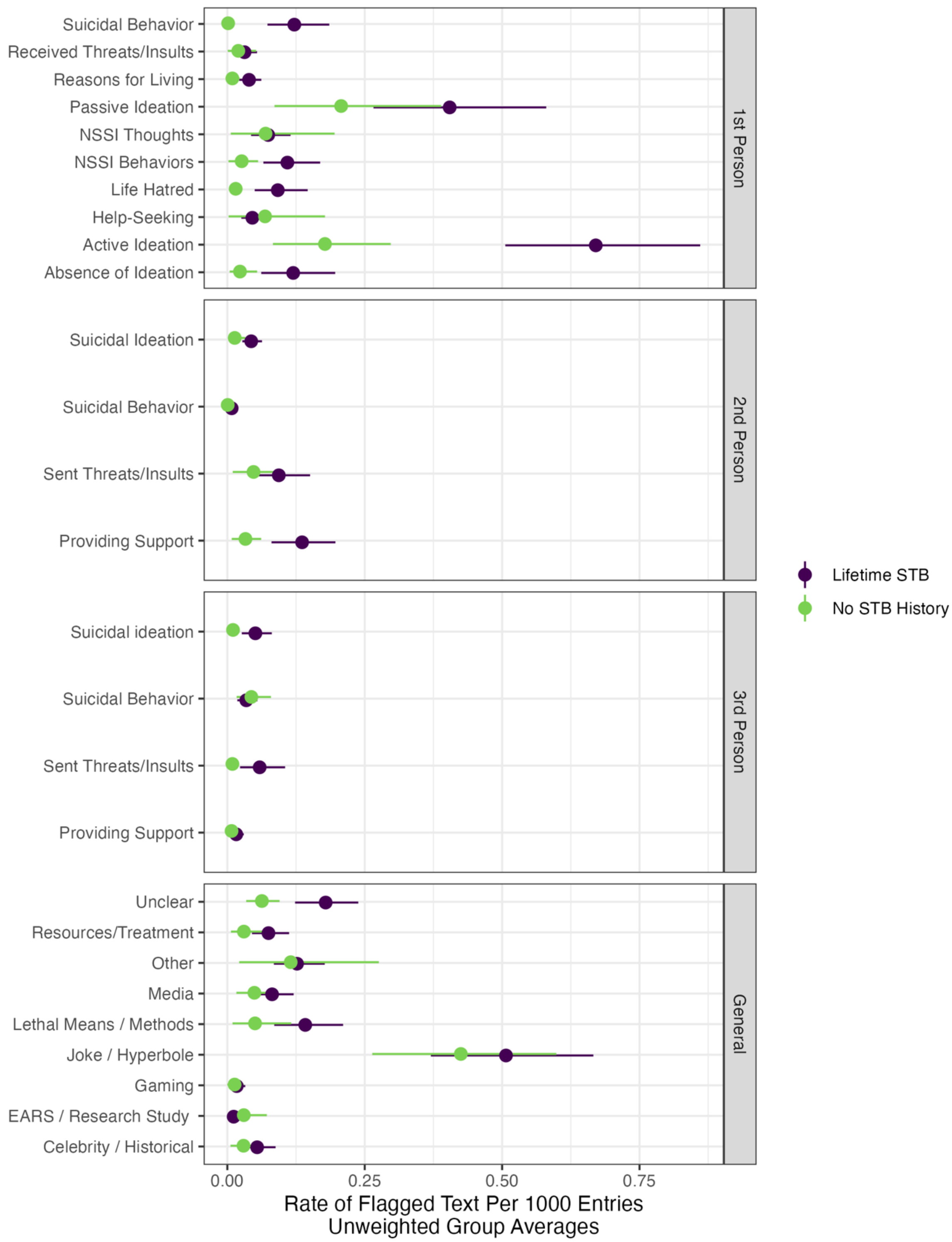

Supplemental Figure 14: Visualization of Average Rates of Flagged Text in Each Human-Labeled Category in the MAPS Cohort as a Function of STB History. Plots show average rates of text flagged and coded in each category per 1,000 text entries in the MAPS cohort for youth with Lifetime STB (purple) and without lifetime STB history (light green). Points and error bars show grand mean unweighted average rates and 95% confidence intervals.

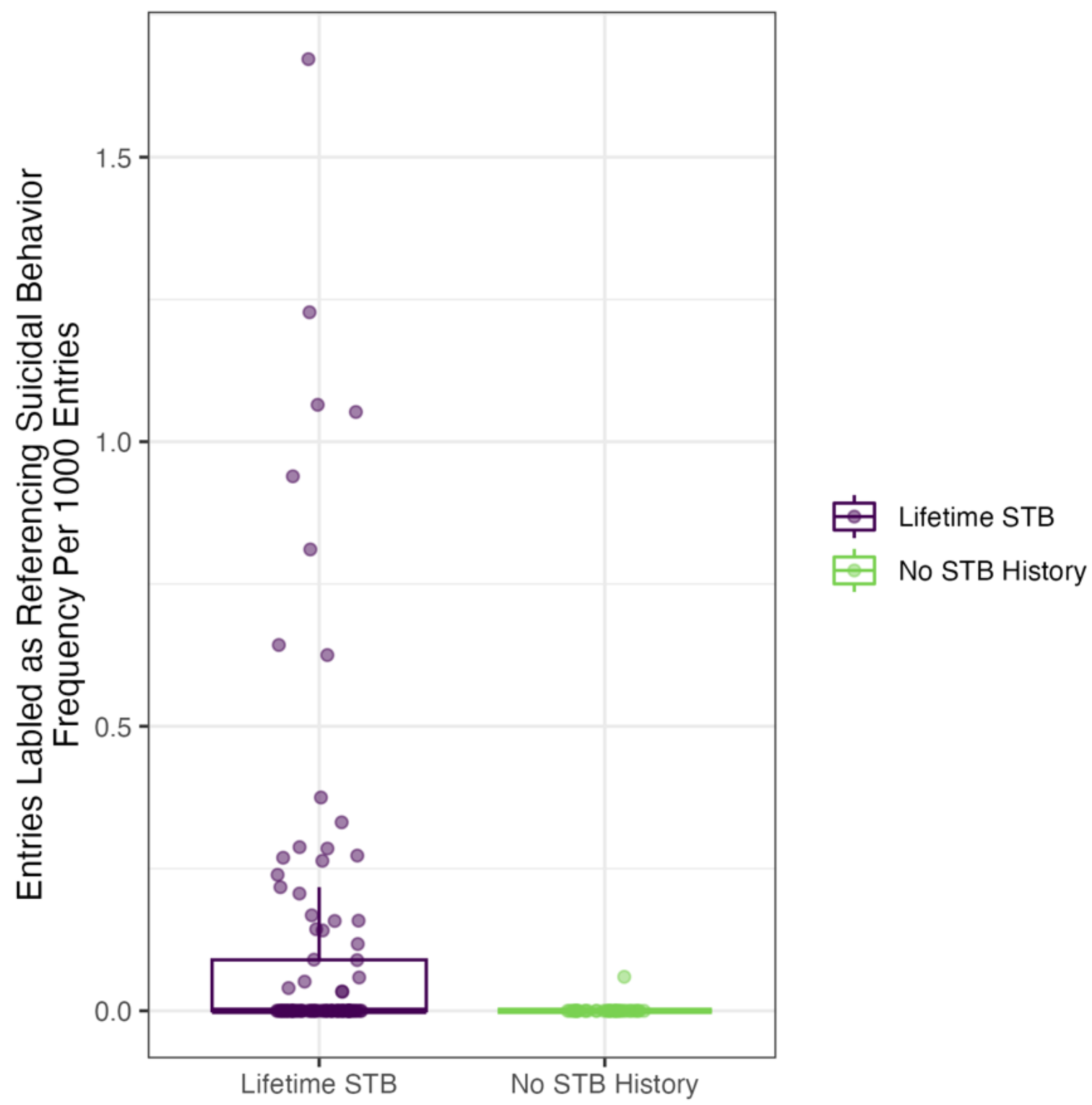

Supplemental Figure 15. Descriptive Plot of Entries Referencing Suicidal Behavior as a Function of Lifetime STB History. Points show the frequency of text referencing suicidal behavior per 1,000 entries for MAPS cohort participants, as a function of Lifetime STB (x-axis). Each point is one participant. Only one participant without STB history had any text coded as referring to suicidal behavior, while slightly under 1/3 of participants of Lifetime STB history had at least some of such entries.

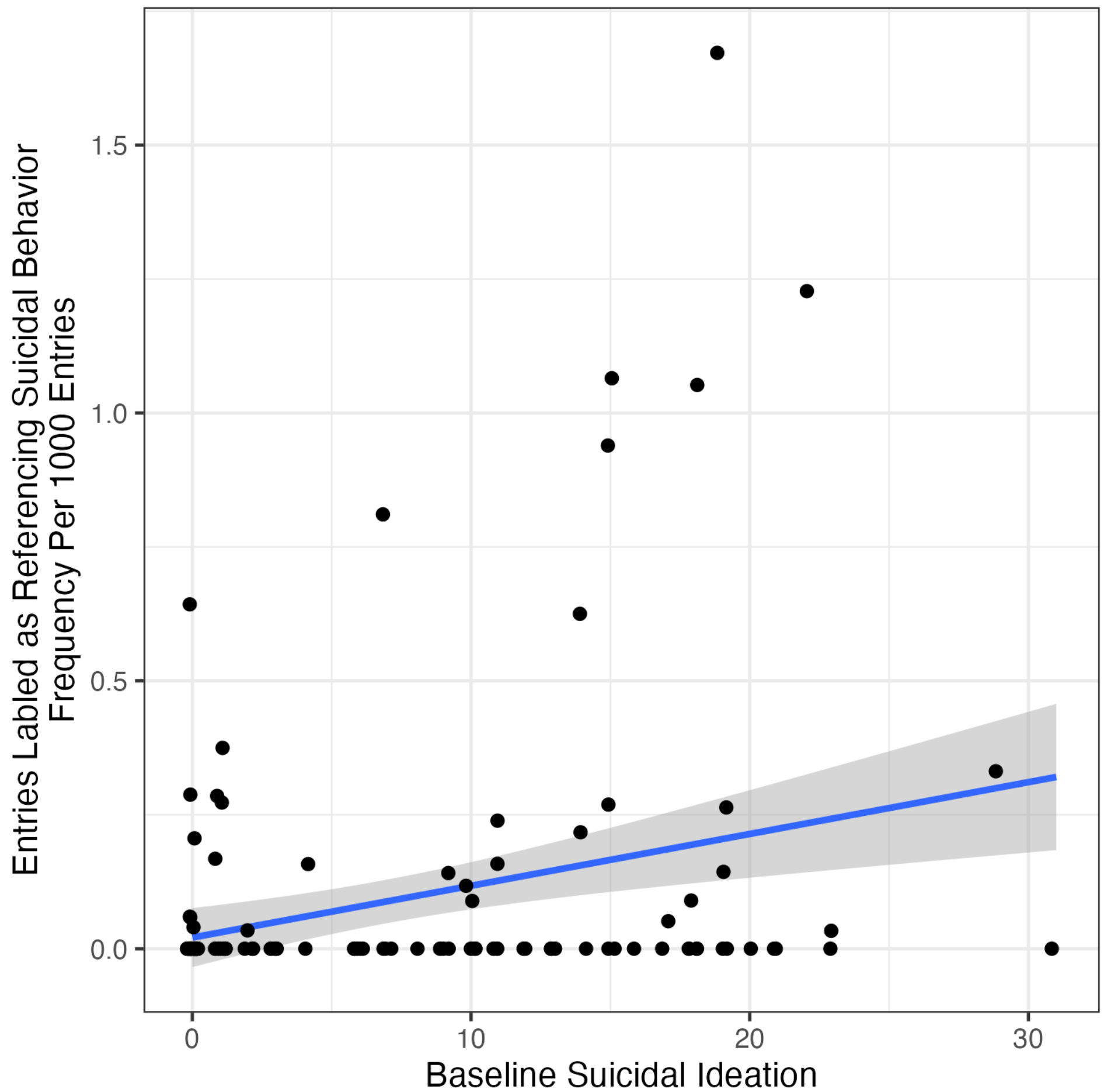

Supplemental Figure 16. Descriptive Plot of Entries Referencing Suicidal Behavior as a Function of Baseline Self-Reported Suicidal Ideation. Points show the frequency of text referencing suicidal behavior per 1,000 entries for MAPS cohort participants, as a function of Baseline Suicidal Ideation (19-Item SSI sum score, x-axis). Each point is one participant. For description, a linear regression and 95% confidence interval is plotted.

Comparison of Lexicon Flagging Along versus Human Labels of Lexicon-Flagged Text

Parallel to the bootstrapping analysis reported in the main text showing that human coding more strongly differentiated MAPS youth with STB history compared to the lexicon alone, we conducted a similar bootstrapping analysis indicating that baseline suicide ideation was more strongly associated with human coding of suicide language compared to lexicon flagging alone ($OR_{Human}/OR_{Lexicon}$=1.21, 95% CI=[1.08, 1.36]).

In addition, we also conducted multilevel logistic regression models where we estimated the interaction term between label type (human, lexicon) and STB history (as well as baseline suicidal ideation) on the probability of a label being present for a given text entry. These models found significant interaction terms for both STB history (β=0.16, 95% CI=[0.12, 0.19]) and baseline suicidal ideation (β=0.30, 95% CI=[0.21, 0.40]) in the MAPS cohort indicating stronger

associations for human labels than lexicon labels alone, providing further evidence of the relative strength of human labels in associations with STB.

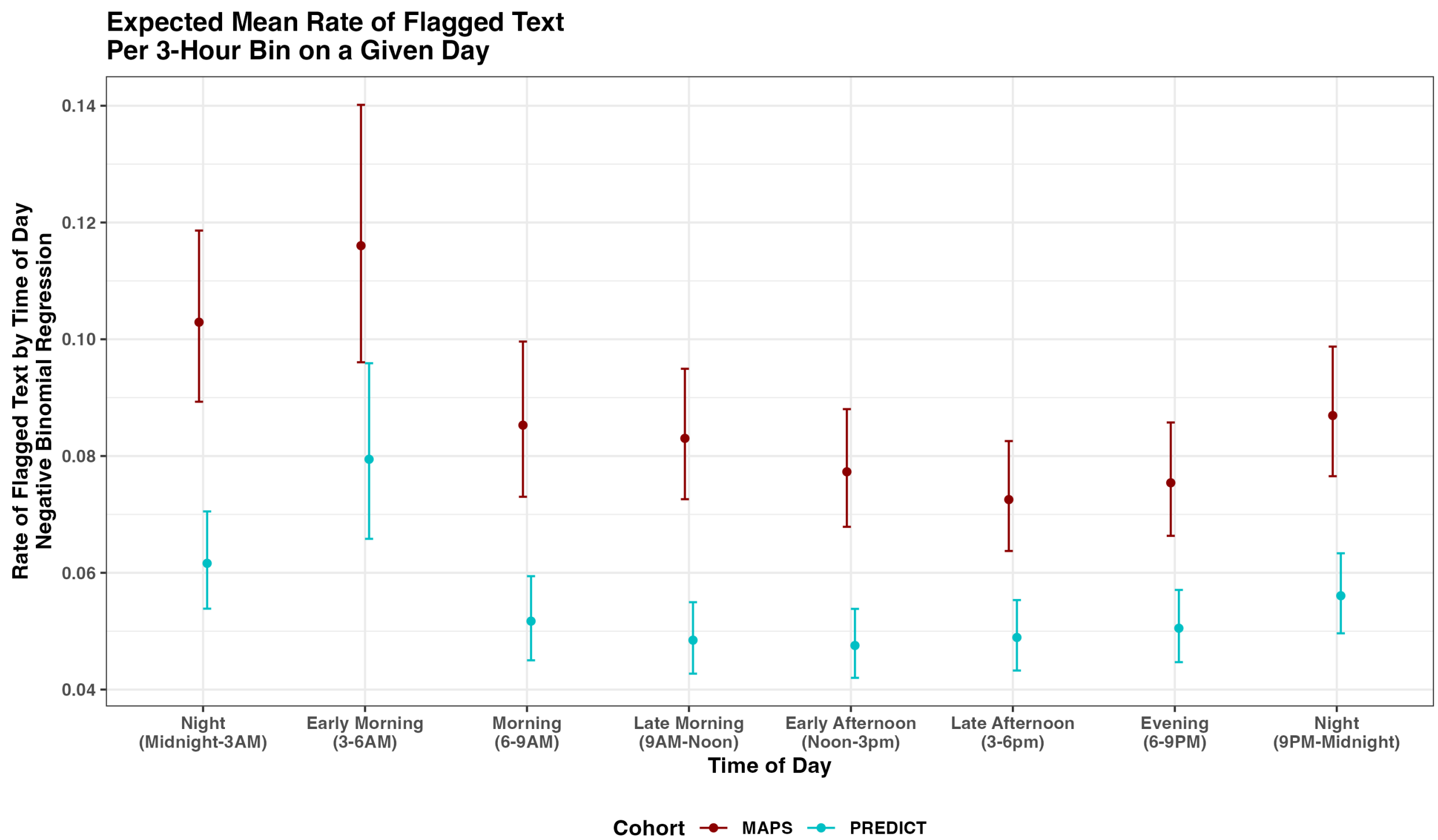

Supplemental Figure 17: Sensitivity Analysis: Suicide Language Frequency as a Function of Time of Day Using Mixed-Effects Negative Binomial Regression: Estimated rates of flagged text are shown per 3-hour window (x-axis) for the MAPS (red) and PREDICT (cohorts). Time of day of text entries was binned into discrete 3-hour windows. Points show mixed-effects negative binomial regression predictions with error bars showing 95% confidence intervals.

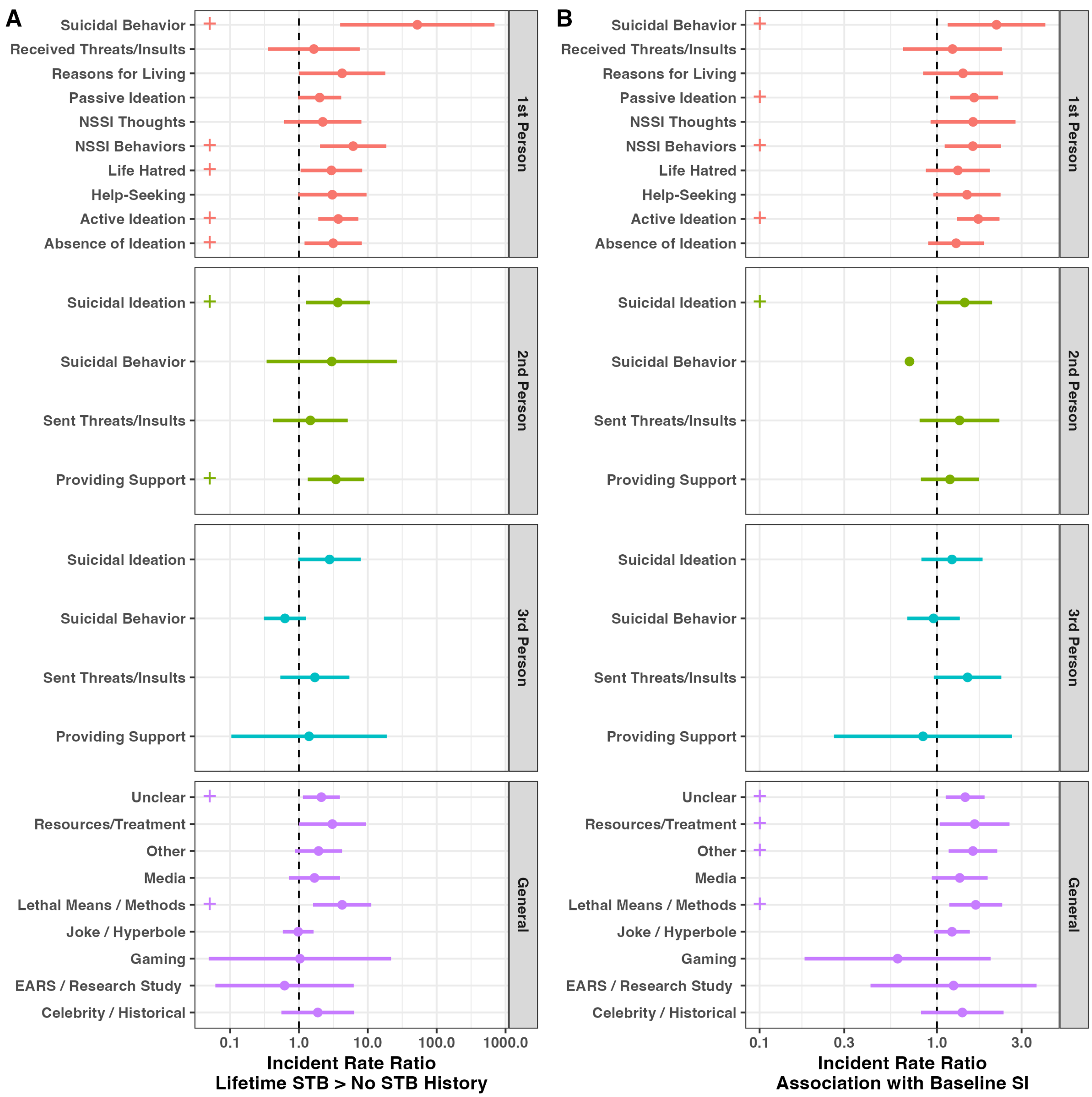

Supplemental Figure 18. Sensitivity Analysis: Associations between STB and Specific Coding Labels for Suicide-Related Text Using Mixed-Effects Negative Binomial Regression. Panel A. Incident rate ratios for differences between youth with lifetime STB history compared to youth with no lifetime STB history (Psychiatric Control) in the MAPS cohort are plotted on the x-axis. The large incident rate ratio for references to suicidal behavior among youth (**Panel A**) was likely driven by the fact that only one participant (out of 41, 2.4%) without STB history had any such entries, compared to 31 participants (out of 99, 31.3%) with lifetime STB history. **Panel B.** Incident rate ratios for associations between baseline self-reported suicidal ideation (standardized) are plotted on the x-axis. In both panels, incident rate ratio estimates and 95% confidence intervals are from mixed-effects regression models where the outcome is the daily rate of language, incorporating the log number of daily entries as the offset

parameter. Note: the model for second-person suicidal behavior did not converge for the association with baseline suicidal ideation, thus only the point estimate is shown (and should be taken with caution).

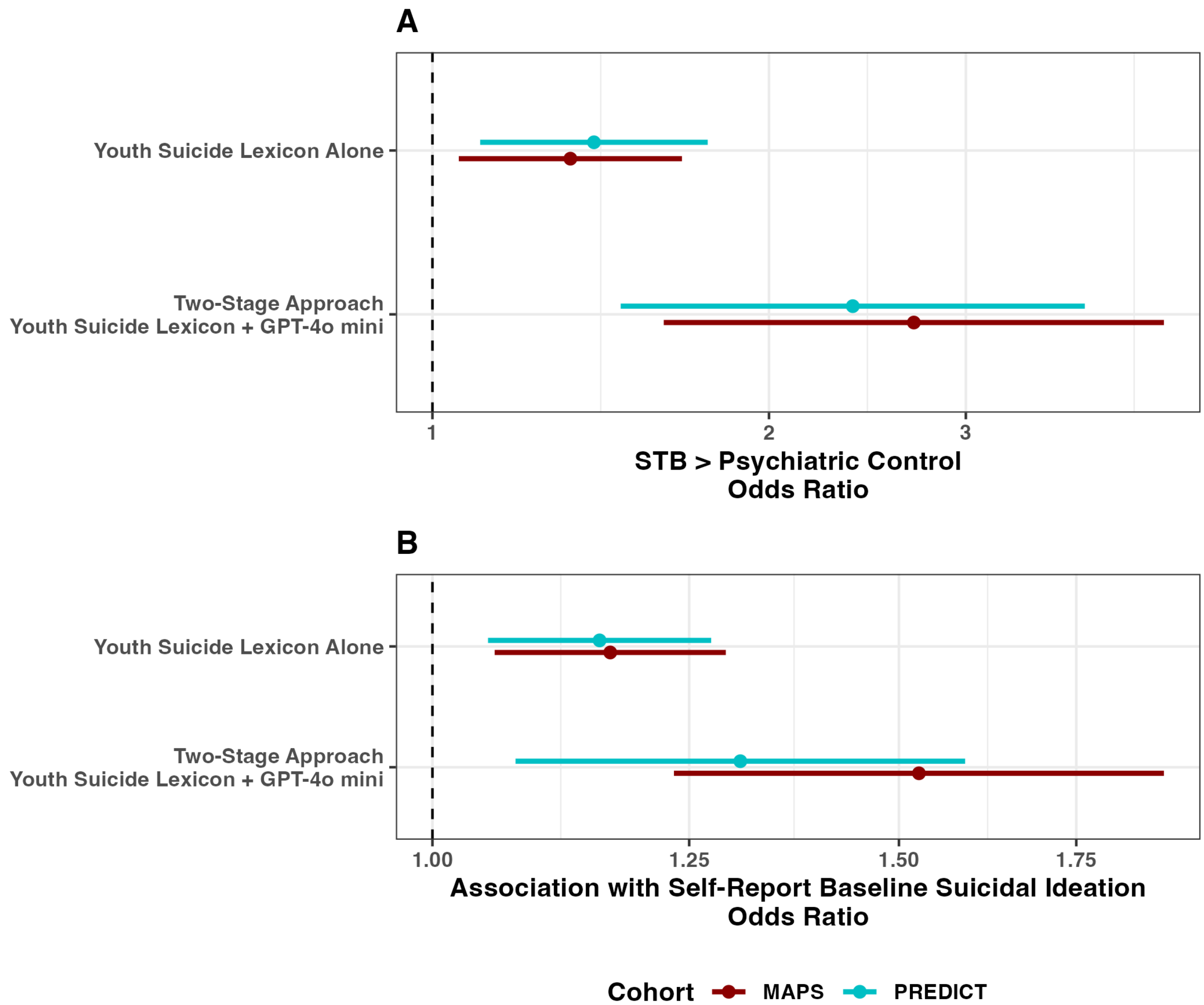

Supplemental Figure 19: Comparison of Lexicon-Only and Two-Stage Lexicon+LLM Approaches in Differentiating Youth with STB History and Baseline Suicidal Ideation. Odds ratios for multilevel logistic regression models are plotted with 95% CI in the MAPS (red) and (PREDICT) cohorts. The outcome of all models was the likelihood of text entries being flagged, either as suicide-related (for the youth suicide lexicon alone), or as suicide-related (by the youth suicide lexicon) and also current, first-person, and authentic (by GPT-4o mini) in a two-stage approach. **Panel A:** Odds ratios show the likelihood of text being flagged for youth with STB history > psychiatric control youth. **Panel B:** Odds ratios show the likelihood of text being flagged as a function of baseline self-reported suicidal ideation (reported via the Beck SSI). As indicated in the main text, bootstrapping analyses indicated that lexicon+LLM odds ratios were significantly stronger than those of the youth suicide lexicon alone, except for in association with self-report baseline suicidal ideation in the PREDICT cohort.

Supplemental References

1. Kaufman, J. *et al.* Schedule for Affective Disorders and Schizophrenia for School-Age Children-Present and Lifetime Version (K-SADS-PL): initial reliability and validity data. *J Am Acad Child Adolesc Psychiatry* **36**, 980–988 (1997).
2. Kovacs, M. *Children's Depression Inventory (CDI2): Technical Manual*. (Multi-Health Systems, Incorporated, 2011).
3. Birmaher, B. *et al.* The Screen for Child Anxiety Related Emotional Disorders (SCARED): Scale Construction and Psychometric Characteristics. *Journal of the American Academy of Child & Adolescent Psychiatry* **36**, 545–553 (1997).
4. Grootendorst, M. BERTopic: Neural topic modeling with a class-based TF-IDF procedure. Preprint at https://doi.org/10.48550/arXiv.2203.05794 (2022).
5. Del Giudice, M. & Gangestad, S. W. A Traveler's Guide to the Multiverse: Promises, Pitfalls, and a Framework for the Evaluation of Analytic Decisions. *Advances in Methods and Practices in Psychological Science* **4**, 2515245920954925 (2021).
6. Steegen, S., Tuerlinckx, F., Gelman, A. & Vanpaemel, W. Increasing Transparency Through a Multiverse Analysis. *Perspect Psychol Sci* **11**, 702–712 (2016).
